# Unraveling LINE‐1 retrotransposition in head and neck squamous cell carcinoma

**DOI:** 10.1002/1878-0261.70063

**Published:** 2025-06-04

**Authors:** Jenifer Brea‐Iglesias, Ana Oitabén, Sonia Zumalave, Bernardo Rodriguez‐Martin, María Gallardo‐Gómez, Martín Santamarina, Ana Pequeño‐Valtierra, Laura Juaneda‐Magdalena, Ramón García‐Escudero, José Luis López‐Cedrún, Máximo Fraga, José M. C. Tubio, Mónica Martínez‐Fernández

**Affiliations:** ^1^ Translational Oncology Research Group, Galicia Sur Health Research Institute (IIS Galicia Sur) SERGAS‐UVIGO, Estrada de Clara Campoamor Vigo Spain; ^2^ Mobile Genomes Lab, Centre for Research in Molecular Medicine and Chronic Diseases (CiMUS) Universidad de Santiago de Compostela Santiago de Compostela Spain; ^3^ Molecular and Translational Oncology Division CIEMAT (ed 70A) Madrid Spain; ^4^ Research Institute Hospital 12 de Octubre (imas12) Madrid Spain; ^5^ Centro de Investigación Biomédica en Red de Cáncer (CIBERONC) Madrid Spain; ^6^ Department of Maxillofacial Surgery University Hospital of A Coruña A Coruña Spain; ^7^ Pathological Anatomy, Faculty of Medicine University Clinical Hospital & Health Research Institute of Santiago de Compostela (IDIS) Santiago de Compostela Spain; ^8^ Present address: Centre for Genomic Regulation (CRG), The Barcelona Institute of Science and Technology Barcelona Spain; ^9^ Present address: Universitat Pompeu Fabra (UPF) Barcelona Spain

**Keywords:** early diagnosis biomarker, field cancerization, head‐and‐neck squamous cell carcinoma, LINE‐1, retrotransposition

## Abstract

The relevant role of LINE‐1 (L1) retrotransposition in cancer has been recurrently demonstrated in recent years. However, the repetitive nature of retrotransposons makes their identification and detection inaccessible for clinical practice. Also, its clinical relevance for cancer patients is still limited. Here, we developed RetroTest, a new efficient method to quantify L1 activation based on targeted sequencing and a sophisticated bioinformatic pipeline, allowing its application in tumor biopsies. Firstly, we performed RetroTest benchmarking to confirm its high specificity and reliability. Then, we unraveled L1 activation in head‐and‐neck squamous cell carcinoma (HNSCC) according to an extensive patient cohort including all tumor stages. L1 retrotransposition estimation revealed a surprisingly early activation in HNSCC progression, contrary to its classical association with advanced stages. In addition, L1 activation together with genomic mutational profiling in normal adjacent tissues supported a field cancerization process, a phenomenon where a tissue develops multiple patches of cells with genetic and/or epigenetic alterations, increasing the risk of cancer development in that area. Overall, our results underline an early L1 activation in HNSCC and field characterization, raising L1 as a promising early diagnostic biomarker and supporting the importance of estimating L1 retrotransposition in clinical practice toward a more efficient diagnosis in HNSCC.

## Graphical abstract

The novel RetroTest method allows the detection of L1 activation in clinical samples with low DNA input, providing global L1 activity and the identification of the L1 source element. We applied RetroTest to a real‐world cohort of HNSCC patients where we reported an early L1 activation, with more than 60% of T1 patients showing L1 activity. Also, the process of field cancerization is supported in HNSCC, as we identified somatic mutations and L1 insertions exclusively in normal adjacent tissue.

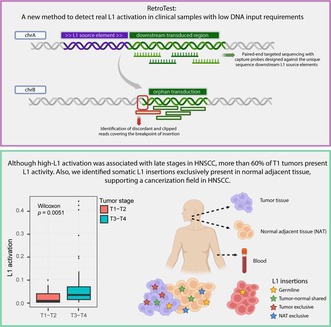

**Abbreviations**
FFPE: formalin‐fixed paraffin‐embedded
HNSCC: head‐and‐neck squamous cell carcinoma
LINE‐1 (L1): long interspersed nuclear element
NAT: normal adjacent tissue
OS: overall survival
PFS: progression‐free survival
PMBCs: peripheral blood mononuclear cells
TD2: orphan L1 transductions
TMB: tumor mutation burden
VAF: variant allele frequency
WGS: whole genome sequencing

## 1 Introduction

Approximately half of the human genome is composed of transposable elements, sequences with the ability to move from one location to another, changing the normal structure of the genome in the places where they are integrated [1, 2]. Among them, long interspersed nuclear element retrotransposons (LINE‐1, L1) represent 17% of the entire DNA content with approximately 500,000 copies, most of them truncated or inactive [3, 4, 5]. Only a small subset of L1 remains active in the human genome, although it stays transcriptionally repressed due to epigenetic mechanisms that prevent the damage that its mobilization would cause [6]. When this repression is lost, L1 activation can cause different diseases, including cancer [7, 8, 9]. Despite the demonstrated impact of L1 activation in cancer genomes, its repetitive nature and dispersion along the genome hinder real activation estimates, preventing its translation into clinical practice.

In the framework of the International Consortium of Pan‐Cancer (PCAWG), our previous analyses showed that somatic L1 insertions represent the major restructuring source of cancer genomes, especially important for head and neck squamous cell carcinoma (HNSCC), which represents the second tumor type with the highest L1 activation [8]. Head and neck cancer is a heterogeneous group of cancers, of which more than 90% are diagnosed as HNSCC, arising in the stratified epithelium of the oral cavity, pharynx, and larynx [9, 10]. The lack of symptoms in the early stages, together with the non‐existent diagnostic biomarkers, leads to most diagnoses at advanced stages, where the 5‐year survival rate is < 50% [10]. Thus, there is an urgent need to find molecular biomarkers that can facilitate an early diagnosis and increase patients' life expectancy.

Here, we aimed to unravel the impact of possible L1 activation along HNSCC progression in a clinical setting by developing a new efficient method based on L1 transductions: RetroTest.

## 2 Materials and methods

### 2.1 Patients and tumor samples

A series of 96 HNSCC patients from 3 different cohorts (*Complexo Hospitalario Universitario de A Coruña* (CHUAC), *Biobanco Vasco*, and *Fundación Pública Galega de Medicina Xenómica* (FPGMX)) was analyzed. All patients understood and signed a written informed consent form. The study methodologies conformed to the standards set by the Declaration of Helsinki. The study methodologies were approved by the local Ethical Committee for Clinical Research of Santiago–Lugo (CEIC 2018/567). Characteristics are specified in Table 1.

**Table 1:** Baseline characteristics of the HNSCC patients and clinicopathological results in the series.

*N* = 96	
Mean age (range)	67.4 (38–90)
Sex	
Female	20
Male	71
NA	5
TNM stage	
T1	16
T2	19
T3	18
T4	34
NA	9
Exitus	
No	50
Yes	38
NA	8
Alcohol	
Drinker	61
Non‐drinker	24
NA	11
Smoking habits	
Smoker	64
Non‐smoker	22
NA	10

### 2.2 DNA isolation

Genomic DNA was extracted from fresh‐frozen tissue and formalin‐fixed paraffin‐embedded (FFPE) samples using AllPrep DNA/RNA and AllPrep FFPE DNA/RNA Mini Kits (Qiagen). DNA quantification and integrity were assessed using the Qubit dsDNA BR Assay Kit in Qubit 4.0 (ThermoFisher) and a 4200 TapeStation system (Agilent).

### 2.3 RetroTest library design and target sequencing

**Fig. 1:**
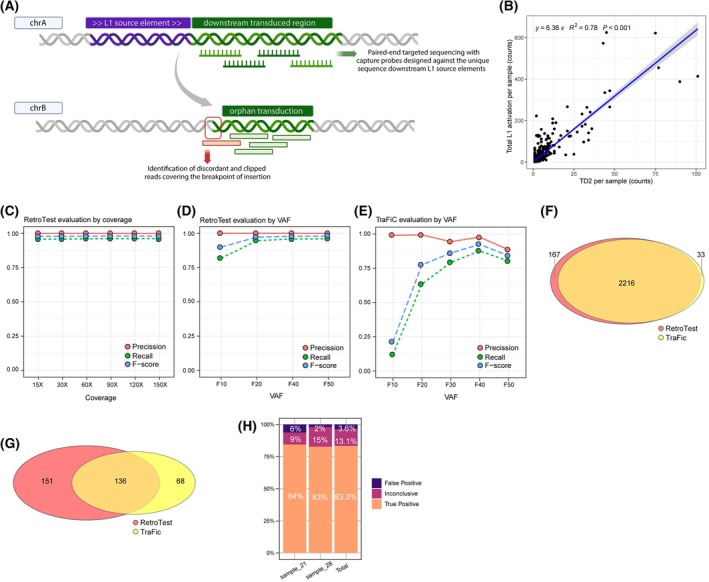
RetroTest design and benchmarking. (A) RetroTest design. (B) Scatterplot and correlation between L1 activation and orphan transductions (TD2) in the International Consortium of PanCancer (PCAWG) data. The number of L1 3′ transductions measured L1 activation in 2,954 cancer genomes from 38 histological cancer subtypes. (C) Performance of RetroTest for different sequencing coverages using an artificially generated hg19 genome with a total of 2,480 randomly distributed L1 transductions at 50% VAF. (D) Performance of RetroTest with respect to the VAF of L1 integrations, using the artificially generated hg19 genome with a total of 2,480 randomly distributed L1 transductions at different VAFs. (E) Performance of TraFiC with respect to the VAF of L1 integrations, using the artificially generated hg19 genome with a total of 2,480 randomly distributed L1 transductions at different VAFs. (F) Venn diagram of the number of L1 insertions detected by RetroTest and TraFiC in the artificial genome with a VAF of 50% for L1 insertions. (G) Venn diagram of the number of L1 insertions detected by RetroTest and TrafiC in the 19 HNSCC samples that underwent both WGS and RetroTest target sequencing. (H) Barchart of the true positive, inconclusive, and false positive retrotransposition events exclusively called by RetroTest after IGV inspection in WGS data of HNSCC sample_21 and sample_28.

There are three different types of retrotransposition: solo‐L1 (TD0), when a partial or complete L1 is retrotransposed; partnered transductions (TD1), in which a L1 and downstream unique sequence are retrotransposed; and orphan transductions (TD2), in which only the unique sequence downstream of the active L1 is mobilized without the associated L1 [6]. During L1 transcription, the transcription machinery sometimes bypasses the L1 polyadenylation signal until a second 3′ downstream polyadenylation site, mobilizing unique sequences downstream of the element in a process called L1 3′ transduction. This occurs in around 10% of the L1 mobilizations [6] and could be used as an indirect measurement of real L1 activation. RetroTest is based on targeted sequencing against the unique sequence downstream of the 124 L1 full‐length competent elements previously described [6]. The main idea behind RetroTest is that discordant read pairs, where one of the mates is mapped to a L1 3′ downstream sequence while the other is mapped to the insertion target sequence, support an insertion. In addition, clipped reads, mapped to the target sequence but containing a discordant extreme blatting to a L1 3′ downstream sequence, are detected to identify the breakpoint of the insertion. Thus, RetroTest identifies L1 insertions through the detection of discordant reads and clipped reads (Fig. 1A).

Sequencing libraries enriched in L1 3′‐transductions were prepared starting from 100 ng of DNA sheared using a Covaris M220 Focused‐Ultrasonicator (Covaris Inc.) in fragments of ~300 bp for FFPE and ~500 bp for frozen tumors. After sonication, fragment size and DNA concentration were assessed with High Sensitivity DNA Assay (Agilent Technologies Inc.). Adaptor‐ligated libraries of HNSCC samples were prepared with the SureSelect Target Enrichment System for Illumina Paired‐End Multiplexed Sequencing using SureSelect XT2 Library Prep Kit (Agilent Technologies Inc.). Briefly, the samples were indexed, amplified, and pooled before hybridization and capture with RNA targeted baits. Captured indexed pools were amplified to obtain final enriched libraries. Quality controls of the library preparation were performed by using D1000 ScreenTape Assay and High Sensitivity D1000 Assay (Agilent Technologies Inc.). The multiplexed samples were sequenced on Illumina MiSeq and NextSeq platforms using 150 bp pair‐end reads with MiSeq Reagent Kit v2 and NextSeq 500/550 Mid Output Kit v2.5 (300 Cycles) (Illumina Inc.), respectively. The mean coverage for tumor samples was 101×, 726× for normal adjacent samples, and 282× for blood samples.

Sequencing reads were mapped to the hg19 reference genome by Burrows‐Wheeler Aligner BWA‐mem [11]. Samtools [12] was used to sort the aligned reads and to index the obtained bam file, applying Bammarkduplicates2 from Picard tools [13] to mark duplicated reads.

### 2.4 RetroTest pipeline

The input of RetroTest is processed BAM files with 150 bp reads, derived from Illumina paired‐end sequencing. The identification of insertion supporting clusters is performed as follows:

- Discordant and clipped read events are searched in the BAM file/s. This step can be performed in a. single sample mode or b. Tumor‐normal matched mode if a germline control is provided. Then, left‐ and right‐clipped read events are realigned to search for supplementary alignments.
- Discordant and clipping reads are organized into genomic bins and then grouped into clusters. Then discordant read pairs are grouped based on mate position while clipped reads are grouped based on the supplementary alignment position. The genomic bins to search for insertions (which correspond to transduced areas) are based on the coordinates of the L1 downstream‐transduced regions.
- For cluster filtering, all clusters without the minimum number of supporting reads, located in unspecific regions, or composed of duplicated reads are discarded. Clusters can also be filtered out based on supplied genomic coordinates and average alignment mapping quality. Additionally, for discordant clusters, those with mates not over the target reference, and those whose mates align over any source element downstream region are discarded. For clipped clusters, those with a supplementary alignment outside the target reference and those whose supplementary alignments map over any source element downstream region are also filtered out.
- Filtered discordant and clipped clusters are grouped into metaclusters, whose precise coordinates are determined by metacluster breakpoints.
- Finally, each metacluster transduction type is determined using the discordant reads around the insertion point, based on the mapping position of the anchor's mate.

Supporting reads can align exclusively to the positive strand and be reported as a PLUS cluster, exclusively to the negative strand and be reported as a MINUS or to both strands and be reported as RECIPROCAL clusters.

The pipeline can be found in the following site: https://gitlab.com/mobilegenomesgroup/RETROTEST.

### 2.5 RetroTest benchmarking

We compared RetroTest performance with Transposon Finder in Cancer (TraFiC), used previously in PCAWG [6], by generating simulated events and conditions.

To simulate orphan transductions, 5Kb of downstream reference genome (hg19) sequence was retrieved for each of the 124 source elements included in the MEIGA‐MEIsimulator database [14], after considering L1 orientation. For each source element, 20 transduction sequences were generated by random 3′ trimming, representing alternative transcription endings. To simulate the insertion events, the reference genome sequence was divided into 10 kb bins, and insertion points were randomly selected among the bins contained by nuclear chromosomes, with the only condition of avoiding GAP regions. DNA sequences from the reference genome and orphan transductions were sequentially merged using custom python commands, while adding MEI characteristic features, including target site duplication, polyA tail, and 5′ truncation.

We generated *in silico* paired‐end reads from this modified genome version with ART v2.5.8 [15] through several commands from MEIGA‐MEIsimulator, an in‐house bioinformatic tool (150 bp length, insert size 350 bp ± 10%), which were subsequently aligned with BWA‐mem v0.7.17 [11, 16] against the hg19 reference genome and further processed with samtools v1.3.1 [12, 16].

We selected read pairs with at least one of the mates mapping on a given set of target regions with Picard FilterSamReads v2.18.14 [13]. We used the resulting bam files to assess sensitivity and specificity under different conditions. To test how our method performs with subclonal events, we simulated transductions at different VAFs (10%, 20%, 40% and 50%). We also studied how coverage affects the detection power of our algorithm. Using Picard DownSampleSam v2.18.14 [13], we subsampled reads from a 150× simulation at 50% VAF under different sequencing depths (15×, 30×, 60×, 90×, 120×, and 150×).

Precision was calculated by dividing the number of true positive calls by the number of total calls. Recall was calculated by dividing the number of true positive calls by the number of total simulated events. True positives, False positives, and False negatives were identified by intersecting the coordinates of simulated events with those of the calls using BEDTools [17].

Besides, Trafic and RetroTest were compared in a subset of HNSCC samples that underwent both WGS (see Methods 2.6) and the RetroTest targeted method. Venn diagrams with the insertions detected by each method were plotted using the *vennDiagram* R package. Finally, for two of the HNSCC samples, all RetroTest‐specific L1 TD2 calls were visually inspected with the Integrative Genomics Viewer (IGV) in the WGS data. To classify a candidate insertion as a true somatic retrotransposition, the following criteria had to be met: (i) at least three supporting reads in the tumor's Illumina WGS data; (ii) no supporting reads in the matched normal Illumina WGS data; and (iii) the presence of at least one retrotransposition hallmark, either target site duplication (TSD) or a poly(A/T) tail, with a precisely defined breakpoint.

### 2.6 Whole genome analyses, mutation profiles, and enrichment analyses

For WGS, Truseq Nano DNA Libraries (350 bp) were constructed and sequenced in a NovaSeq6000 Illumina platform (150 bp paired‐end) in an external service (Macrogen). Sequencing reads from tumor tissue and normal adjacent tissue (NAT) were mapped to the hg19 reference genome by Burrows‐Wheeler Aligner BWA‐mem [11, 16] v0.7.17. samtools [12, 18] v1.9 was used to sort the aligned reads and to index the obtained bam file, applying Picard Bammarkduplicates2 [13] to mark duplicated reads. After that, Mutect2 [19], from the Genome Analysis Tool Kit (GATK) [20] v4.1.1.0, was used to perform SNVs and INDELs calling. Variants were filtered with FilterMutectCalls (GATK) (considering ASCAT [21] normal contamination estimation), following standard thresholds, and annotated using the Ensembl Variant Effect Predictor (VEP) [22] v100.2. We selected those probably pathogenic variants, following SIFT [23, 24], PolyPhen [25], and VEP Impact annotations. Then, we ensured our variants were somatic by filtering those with an allele frequency equal to or higher than 0.01 in the 1KGP, ESP, or genomAD populations, assessing they are not common in the population.

To analyze the mutational profile of NAT, we also sequenced blood for 4 patients. We used again mutect2 v4.1.7.0 to perform joint SNVs and INDELs calling both for tumor and NAT, following best practices [26, 27]. In this case, GATK FilterMutectCalls now considered cross‐sample contamination estimates performed by GATK CalculateContamination. We considered as common between NAT and tumor those variants presenting more than one supporting read in both samples, while those exclusive to NAT presented more than one supporting read in this sample and none in the tumor.

Enrichment analyses were performed with the enrichr [28] R package using the following reference databases: Human MSigDB collections [29, 30] (MSigDB_Hallmark_2020, MSigDB_Oncogenic_Signatures, and MSigDB_Computational) BioPlanet_2019 [31], KEGG_2019_Human [32, 33], WikiPathways_2019_Human [34, 35], GO_Molecular_Function_2018, and GO_Biological_Process_2018 [36, 37]. We used the following transcription factor binding motifs databases: TRANSFAC_and_JASPAR_PWMs [38] and ChEA_2016 [28, 39].

### 2.7 Statistical analyses

The association between L1 transductions (corrected by coverage) and patient clinical features was assessed by multiple linear regressions. Wilcoxon or Fisher tests, depending on sample size, were applied. Overall survival (OS), progression‐free survival (PFS), and survival probability analyses were performed with the *survminer* and *survival* R packages, using the log‐rank test. The association between survival and clinical variables was evaluated by Cox regression.

## 3 Results

### 3.1 RetroTest benchmarking

RetroTest is designed to capture the mobilized and downstream‐transduced unique sequences from orphan L1 transductions (TD2), used as barcodes (Fig. 1A). These barcodes can identify unequivocally the insertions caused by the 124 L1 source elements active in cancer (Table S1) [6, 8]. Thus, we focused RetroTest probe design on the first 5,000 nucleotides adjacent to the L1 3′ regions for each L1 source element, as the regions most frequently transduced [6]. Since TD2 was used as an indirect measure of total L1 activation, we first compared the frequency of both events, finding a strong linear correlation with a 1:6 ratio (*y* = 6.33*x*, *R* ^2^ = 0.78, *P* < 0.001, Fig. 1B). Next, we optimized the laboratory protocol for both FFPE and fresh‐frozen tissues and developed the associated bioinformatic pipeline. We evaluated the performance and accuracy of RetroTest by generating an artificial cancer genome, where we distributed randomly a total of 2,480 L1 transductions. Using simulations for different sequencing depths, RetroTest obtained a precision of around 0.99 in all cases and a recall of around 0.96 (Fig. 1C). We also evaluated the performance depending on the variant allele frequency (VAF): RetroTest obtained a precision of around 1, decreasing for lower VAFs, and a recall ranged from 0.81 to 0.96, augmenting as increasing the VAF (Fig. 1D).

Then, we compared RetroTest and the classical TraFiC, used in PCAWG [6]. As TraFiC is designed to work only with standard WGS 30x data, we compared the analysis, varying exclusively the VAFs. TraFiC precision ranged from 0.99 to 0.88 and recall ranged from 0.11 to 0.87 as VAF increased (although the maximum recall was obtained for VAFs of 40%, being 0.87) (Fig. 1E) (Table S2). We compared the performance of both methods by intersecting both calls in the mock tumor genome. Using a VAF of 50%, most of the variants were called by both methods, concretely 2,216, while RetroTest exclusively called 167, and TraFiC 33 private events, most of them resulting in false positives according to IGV (Fig. 1F).

To further validate our tool, we compared the TD2 events identified by RetroTest and TraFiC in a subset of 19 HNSCC samples that underwent both WGS and targeted sequencing, finding that 38.3% (136/355) of the events were detected by both methods, while 42.5% (151/355) were exclusively identified by RetroTest, and 19.2% (68/355) were exclusively detected by TraFiC (Fig. 1G, Table S3).

IGV inspection of TD2 events that were exclusively called by RetroTest on WGS data of the HNSCC tumors sample_21 (32 RetroTest exclusive events) and sample_28 (52 RetroTest exclusive events) (Table S3) confirmed that 83.3% of RetroTest private retrotranspositions were true positive events (70 out of 84), as they presented at least three supporting reads in the tumor, there were no supporting reads in the matched normal, and presented a retrotransposition hallmark (Fig. S1). The 13.1% corresponded to inconclusive results (11 out of 84), where no reads could confirm a true positive event, but no evidence in the region suggested a false positive (indicating these were likely subclonal L1 events present only in the RetroTest library). Finally, only 3.6% were false positives (3 out of 84 events) (Fig. 1H, Data S1).

### 3.2 L1 activation in HNSCC

**Fig. 2:**
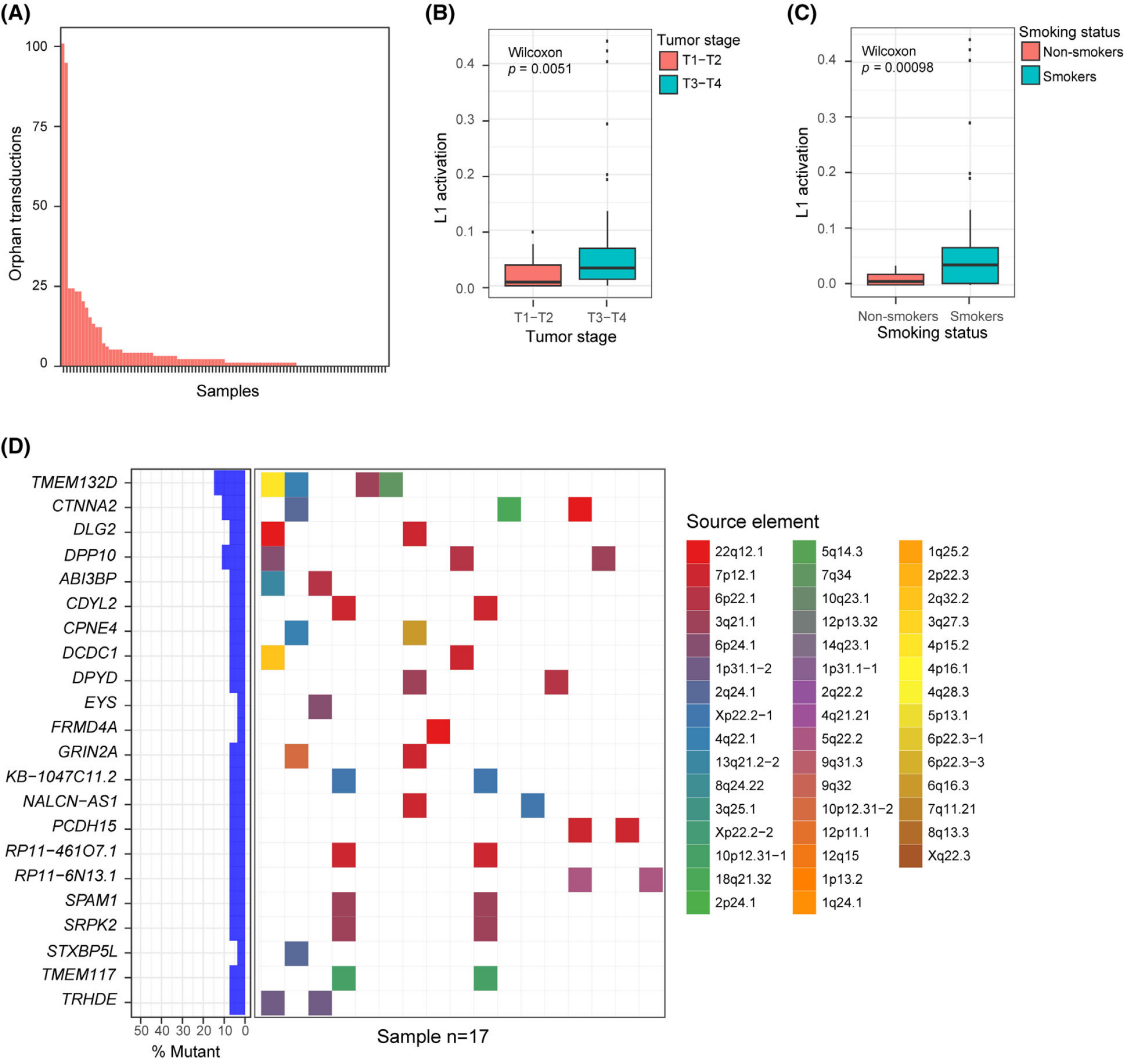
L1 activation measured by RetroTest in the HNSCC cohort (*n* = 96). (A) Quantification of L1 activation in HNSCC tumors as the number of orphan transductions detected by RetroTest. (B) Boxplot of L1 activation with respect to early (T1–T2) and advanced (T3–T4) TNM stages. Differential activation *P*‐value was derived by the Wilcoxon test. Boxplot whiskers extend to the smallest and largest values within 1.5 times the interquartile range of the lower and upper quartiles (Q1–Q3). Outliers outside this range are plotted as individual points. To correct coverage‐related bias, L1 activation was calculated as the number of TD2 divided by its median coverage. Samples T1–T2 (*n* = 35), samples T3–T4 (*n* = 52). (C) Boxplot of L1 activation with respect to smoking status. Differential activation *P*‐value was derived by the Wilcoxon test. Boxplot whiskers extend to the smallest and largest values within 1.5 times the interquartile range of the lower and upper quartiles (Q1–Q3). Outliers outside this range are plotted as individual points. To correct coverage‐related bias, L1 activation was calculated as the number of TD2 divided by its median coverage. Smoker (*n* = 64), non‐smoker (*n* = 22). (D) Oncoplot showing the genes affected by L1 insertions and their original source elements. A total of 17 patients presented more than 2 genes affected by L1 insertions from 46 different source elements.

Once confirmed the accuracy and precision of RetroTest, we decided to apply it to a 96 HNSCC cohort from stages T1 to T4 (Table 1). We detected L1 activation in 71.8% of the patients (Fig. 2A, Table S4), out of which 49.3% showed high activity (beyond the median) (Table 2). When the activation was studied according to tumor stages, advanced disease (T3–T4) showed statistically higher L1 activation compared with early stages (T1–T2) (*P* = 0.0051) (Fig. 2B). Specifically, L1 activation was detected in 56.2% of T1 tumors, 57.9% of T2, 94.4% of T3, and 73.5% of T4 tumors (Table 2). The detection of L1 activation in all the tumor stages, even in the first stages, indicates early L1 activation.

**Table 2:** Number of HNSCC patients showing L1 activation along TNM stages.

	TNM stage			
	T1	T2	T3	T4
L1 active	9	11	17	25
L1 inactive	7	8	1	9

Considering different clinical characteristics, we did not find a statistically significant association between L1 activation and alcohol consumption (*P* = 0.14) or sex (*P* = 0.055). Smoker patients showed statistically significantly higher L1 activity than non‐smokers (*P* = 0.00098) (Fig. 2C). No association between L1 activation and survival probability was detected (*P* = 0.86 active vs. inactive, *P* = 0.28 high vs. low rate) (Fig. S2). When considering only T1 patients, those with L1 activation suggested a potential trend of reduced survival probability although not reaching statistical significance (*P* = 0.77).

Finally, we identified L1 transductions inside genes; concretely, we found 22 genes harboring transductions in at least two patients throughout 17 patients. Since RetroTest can identify the source L1 element, we unravel a few source elements that resulted very active in HNSCC, especially the 22q12.1 (Fig. 2D).

### 3.3 HNSCC mutation profile and L1 activation

**Fig. 3:**
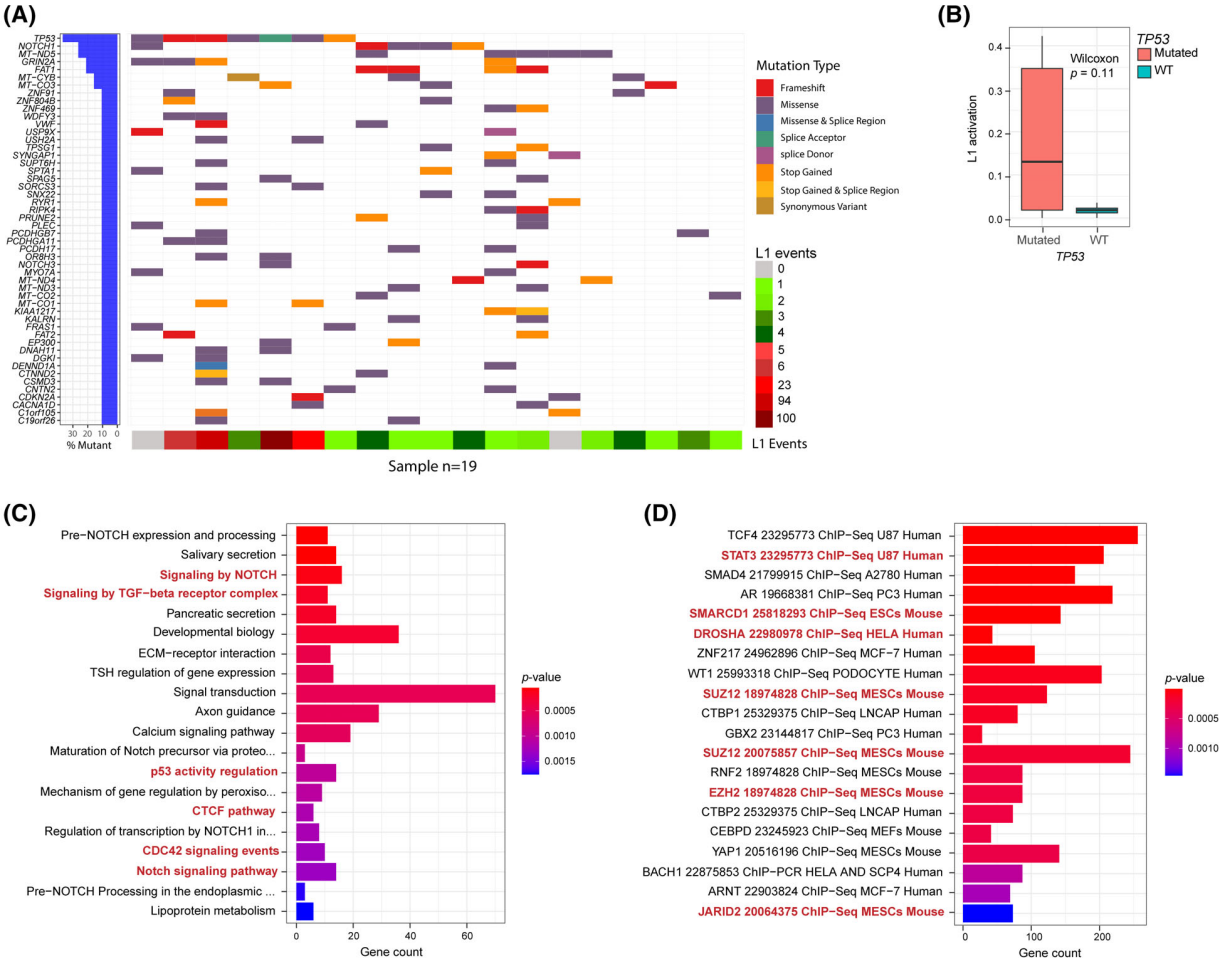
Characterization of the mutational profile of HNSCC patients by WGS (*n* = 19). (A) Oncoplot showing the genes harboring somatic mutations in HNSCC patients. The type of mutation in each gene and the L1 activation (number of L1 insertions in each patient) is shown. (B) Boxplot of L1 activation with respect to TP53 mutation or wild‐type status. Boxplot whiskers extend to the smallest and largest values within 1.5 times the interquartile range of the lower and upper quartiles (Q1–Q3). Outliers outside this range are plotted as individual points. To correct coverage‐related bias, L1 activation was calculated as the number of TD2 divided by its median coverage. Differential activation *P*‐value was derived from the Wilcoxon test. *TP53* mutated (*n* = 7), WT (*n* = 12). (C) Barplot of the Pathway enrichment analysis based on the 918 somatically mutated genes. Enrichment *P*‐values were calculated with the Fisher exact test. (D) Barplot of the transcription factor binding enrichment analysis based on the 918 somatically mutated genes. Enrichment *P*‐values were calculated with the Fisher exact test.

We obtained WGS data from 19 tumor samples measured also by RetroTest. To detect only somatic variation, their corresponding paired‐normal samples were included as germline control. We detected a median of 40 single nucleotide variants (SNVs) and INDELs, identifying a total of 1012 somatic variants, affecting 918 genes (Table S5). We did not detect a correlation between L1 activation and the general tumor mutation burden (TMB) (Fig. 3A). Our results showed that the most frequently mutated gene was *TP53* (36.8%), followed by *NOTCH1* (26.3%), *MT‐ND5* (26.3%), *FAT1*, and *GRIN2A* (21.1%). Interestingly, we found that most of the patients with *TP53* mutations showed also high L1 activity (71.4%). In fact, when we compared the L1 activation with the *TP53* mutation, we found an association tendency (*P* = 0.11) (Fig. 3B). Enrichment analyses with the mutated genes showed involved key processes for cancer progression such as *Notch signaling*, *TGFß signaling*, and again *p53 activity regulation* (Fig. 3C) (Table S6) and the alteration of transcription factors related to epigenetic mechanisms including Polycomb (*EZH2*, *SUZ12*) (Fig. 3D) (Table S7).

### 3.4 Profiling normal adjacent tissues to the HNSCC tumor (NAT)

**Fig. 4:**
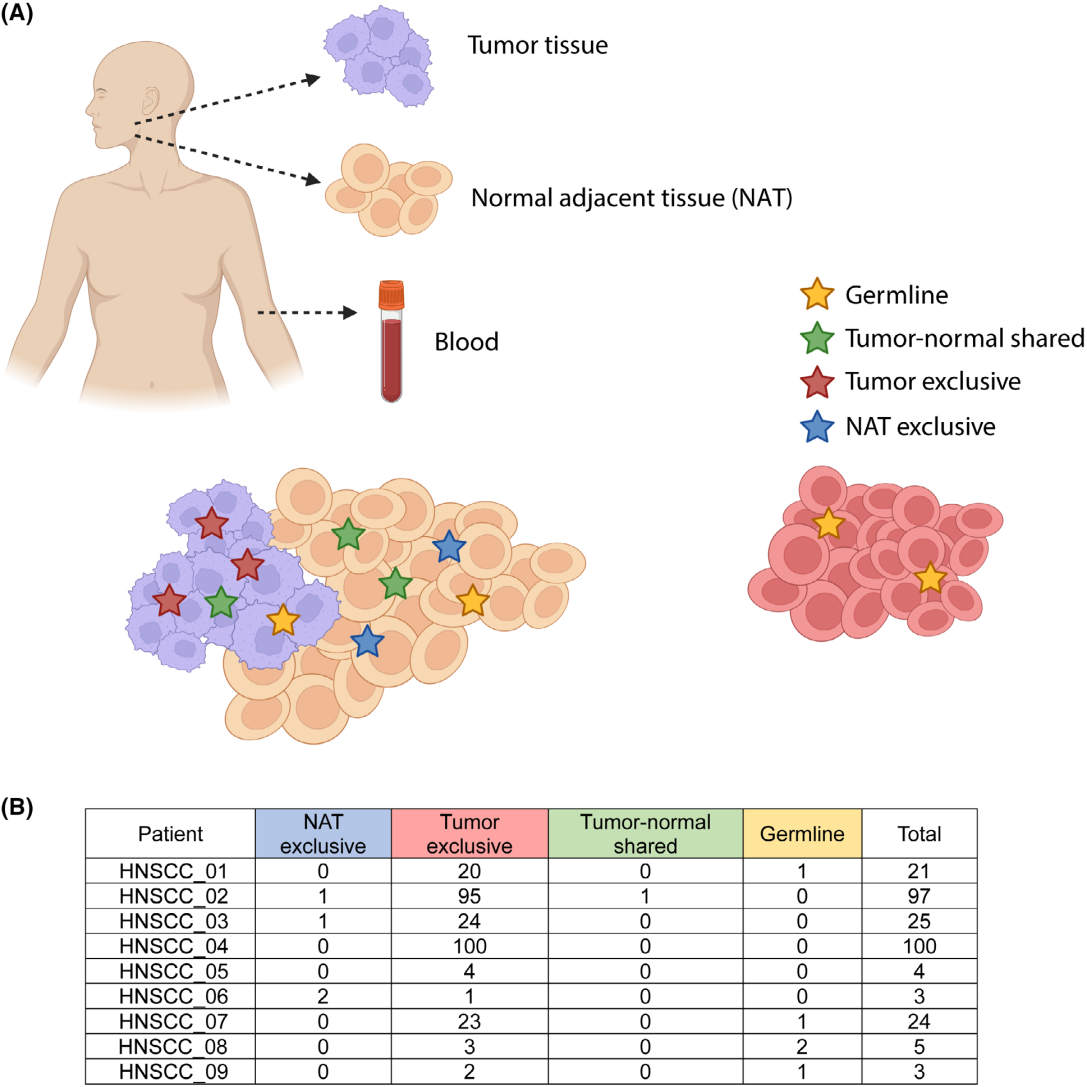
Evaluation of L1 activation in normal adjacent tissue. (A) Schematic representation of the evaluation of the field cancerization process. (B) Number of L1 active elements in normal adjacent tissue of HNSCC patients (*n* = 9), compared to L1 activation in paired tumor tissue and PBMCs as germline control from the same patients.

We decided to evaluate a possible field cancerization process, in which the normal cell population is replaced by cancer‐primed cells, without anatomical or morphological changes, but are already premalignant at the molecular level. We analyzed the available normal samples obtained from NAT, with the peripheral blood mononuclear cells (PBMCs) used as germline control (Fig. 4A). We detected a total of 25 high‐impact and/or possibly pathogenic somatic variants affecting the NAT (Table S8). Most of these specific mutations (*n* = 20; 80%) were exclusive to NAT, including those affecting key genes such as *NOTCH1* (the most mutated gene), *FAT1*, or *PPARD*; while 20% were shared between NAT and tumor tissue, affecting genes such as *CDKN2A*.

To elucidate whether L1 further supports field cancerization, we evaluated 9 NAT by RetroTest and compared the L1 elements active in the tumor, in the NAT, and in their corresponding paired germline. We could confirm that most of the L1 activation was present only in the tumor; 5 insertions resulted in germline, and 1 element was shared by the tumor and NAT. Surprisingly, 4 insertions appeared exclusively in NAT (Fig. 4B). Thus, we could confirm field cancerization and demonstrate that L1 is already active in NAT, supporting once again its early activation in HNSCC.

## 4 Discussion

Since the recently demonstrated high impact of L1 in cancer genomes [6, 8], L1 has been evaluated as a cancer biomarker in different studies assessing its activity by different approaches based on expression levels [40, 41, 42, 43]. However, most L1 sequences are truncated and not functional, so results can present important biases, while its translation into clinical routine is challenging due to RNA/protein instability. Other proposed technologies are based on DNA full‐length L1 capture sequencing [44], but present the same bias associated with the real L1 activation and important input DNA requirements, unaffordable for clinical practice, besides not identifying which L1 elements are active and how much each one contributes to the activation. The most recent approaches are based on the identification of L1 insertions as real L1 activation measures (TraFiC [6], xTea [45], MELT [46] and Mobster [47]) using Whole Genome or Whole Exome Sequencing, not affordable for most of the hospitals. Moreover, they are based on short reads, which hamper the detection of insertions in highly repetitive or complex rearrangement regions. More recently, long reads sequencing has arisen (xTea [45], PALMER [48]), but again their input DNA requirements remain unaffordable for most clinical biopsies. Thus, incorporating L1 activation into clinical practice requires new standardized methods.

We present RetroTest: a new method to detect real L1 activation in clinical samples with low DNA input requirements, both from fresh/frozen or FFPE biopsies. Its novelty and power lie in that it can identify unequivocally the insertions caused by 124 L1 source L1 elements active in cancer in a more cost‐effective manner than previously proposed approaches. Our method not only offers global L1 estimates but also identifies the active L1 source element. Our benchmarking supported a high precision and recall for RetroTest, even detecting subclonal insertions. The high coincidence with TraFic, in both simulations and real‐world data, supports the high potential for this new methodology.

L1 somatic retrotransposition is the second most frequent type of structural variant in HNSCC genomes [8]. In agreement, we detected that most HNSCC patients (75%) present L1 activation, nearly half of them with high levels. This activation was higher in late stages, as described in Barret's esophagus, where lower L1 activity appeared in early stages, increasing with cancer progression [49]. We found a surprisingly early activation in the first stages of HNSCC, with activation of L1 in 62–63% of the T1 tumors. These data pointed toward L1 activation as an early event in the configuration of HNSCC genomes and, thus, in the development of the disease. In fact, T1 patients with L1 activation tended to present a worse prognosis.

We found an association between high L1 activity and smoking habits. Previous studies have reported higher hypomethylation rates of L1 in smokers [50]. Since 75% of HNSCC are associated with tobacco [51], this mechanism could be responsible for the high L1 activity levels, since it represents the best‐demonstrated mechanism preventing L1 reactivation [6, 52, 53]. In fact, L1 hypomethylation has been associated with a worse prognosis in HNSCC [54, 55] and found to be an early event in colorectal, gastric, and oral cancers [56, 57, 58].

The source element L1 most active in our cohort was at 22q12.1, coincident with previous results [10, 59, 60]. This L1 has been identified as the intact L1 mRNA most highly expressed in breast, ovarian, and CRC [61, 62], and the L1 element accounting for most transductions in CRC [52], indicating the same hottest activity in HNSCC.

We found no correlation between L1 activity and TMB, but an association between *TP53* mutation and L1 activation, in line with previous studies suggesting that *TP53* can repress L1 mobilization [8, 61, 63, 64]. We also identified an epigenetic regulation, especially related to the repressive complex Polycomb. Intriguingly, Mangoni et al. have deciphered that L1 RNAs can act as long non‐coding RNAs and directly interact with Polycomb during brain development and evolution [65]. We previously reported that Polycomb could regulate lncRNA *HOTAIR* in bladder cancer [66] and Ishak et al. demonstrated an EZH2‐dependent silencing of genomic repeat sequences, including L1 elements [67]. Therefore, additional analyses are required to further address whether this epigenetic network plays a role in cancer genome reorganization.

We evaluated the presence of field cancerization, finding exclusive somatic mutations in the NAT and a small proportion shared with the tumor. Several studies have described patchworks of different clones in normal tissues, even bearing driver mutations [68, 69]. We found *NOTCH1* and *FAT1* as the most mutated genes, as Martincorena's results in normal skin and esophagus [68, 70], suggesting the presence of a precancerous or cancer invasion field. Finally, several L1 mobilizations were exclusively found in NAT, and only one appeared shared with the tumor, again supporting a cancerization field. Somatic L1 retrotranspositions were also described in normal urothelium and colorectal epithelium, although with much lower rates than in cancer [71, 72, 73], and in pre‐tumor samples including Barrett's esophagus and colorectal adenomas [49, 59, 74, 75].

## 5 Conclusions

In conclusion, L1 activation arises as a reinforced early event in the natural history of HNSCC, even in pre‐tumor stages, with high potential as a field cancerization and early diagnosis HNSCC biomarker.

## Conflict of interest

The authors declare no competing interests.

## Author contributions

JBI and AO contributed equally: Conceptualization, Methodology, Software, Data curation, Investigation, Validation, Visualization, Writing – review and editing. SZ: Conceptualization, Investigation, Methodology, Software, Validation, Writing – review and editing. BRM: Conceptualization, Methodology, Software. MGG: Conceptualization, Visualization, Writing – review and editing. MS: Methodology, Validation. APV: Conceptualization, Investigation. LJM: Methodology, Conceptualization, Writing – review and editing. RGE: Methodology, Writing – review and editing. JLLC: Methodology. MF: Funding acquisition. JMCT: Conceptualization, Funding acquisition. MMF: Writing –review & editing, Writing – original draft, Supervision, Resources, Investigation, Project administration, Funding acquisition, Conceptualization. All authors approved the final version. All authors give consent for the publication of this manuscript.

## Supporting information


**Data S1.** Compressed file containing the IGV screenshots for all the RetroTest exclusive insertions inspected in sample_21 and sample_28 WGS data, classified as true positives (TPs), false positives (FPs), and unconclusive. Both the tumor and normal BAM files were included in each screenshot.

**Fig. S1.** Integrative Genomics Viewer inspection of WGS data of RetroTest private retrotranspositions.
**Fig. S2.** Kaplan–Meier curves for overall survival in HNSCC cohort with respect to (A) L1 activation status (active vs inactive) and (B) L1 activation rate (with respect to the median, being high above vs low as below the median). Log‐rank test was used to calculate the *P*‐value.

**Table S1.** Coordinates of the 124 source elements targeted by RetroTest.
**Table S2.** RetroTest benchmarking.
**Table S3.** TraFiC vs RetroTest performance in a real‐world HNSCC cohort.
**Table S4.** L1 orphan transductions detected by RetroTest in the whole HNSCC cohort.
**Table S5.** HNSCC somatic variants.
**Table S6.** Gene pathways affected by HNSCC somatic variants.
**Table S7.** Transcription factors binding to genes affected by HNSCC somatic variants.
**Table S8.** HNSCC NAT somatic variants.

## Data Availability

Whole Genome Sequencing data from the HNSCC cohort is deposited into the Sequence Read Archive (SRA) repository under the following BioProject ID: PRJNA1053897. RetroTest pipeline is publicly available at https://gitlab.com/mobilegenomesgroup/RETROTEST.
