# Supplementary figures and images for: Unraveling LINE‐1 retrotransposition in head and neck squamous cell carcinoma
Source: Mol Oncol. 2025 Jun 4;19(12):3769–83. doi: 10.1002/1878-0261.70063 (PMC12688165; doi:10.1002/1878-0261.70063)

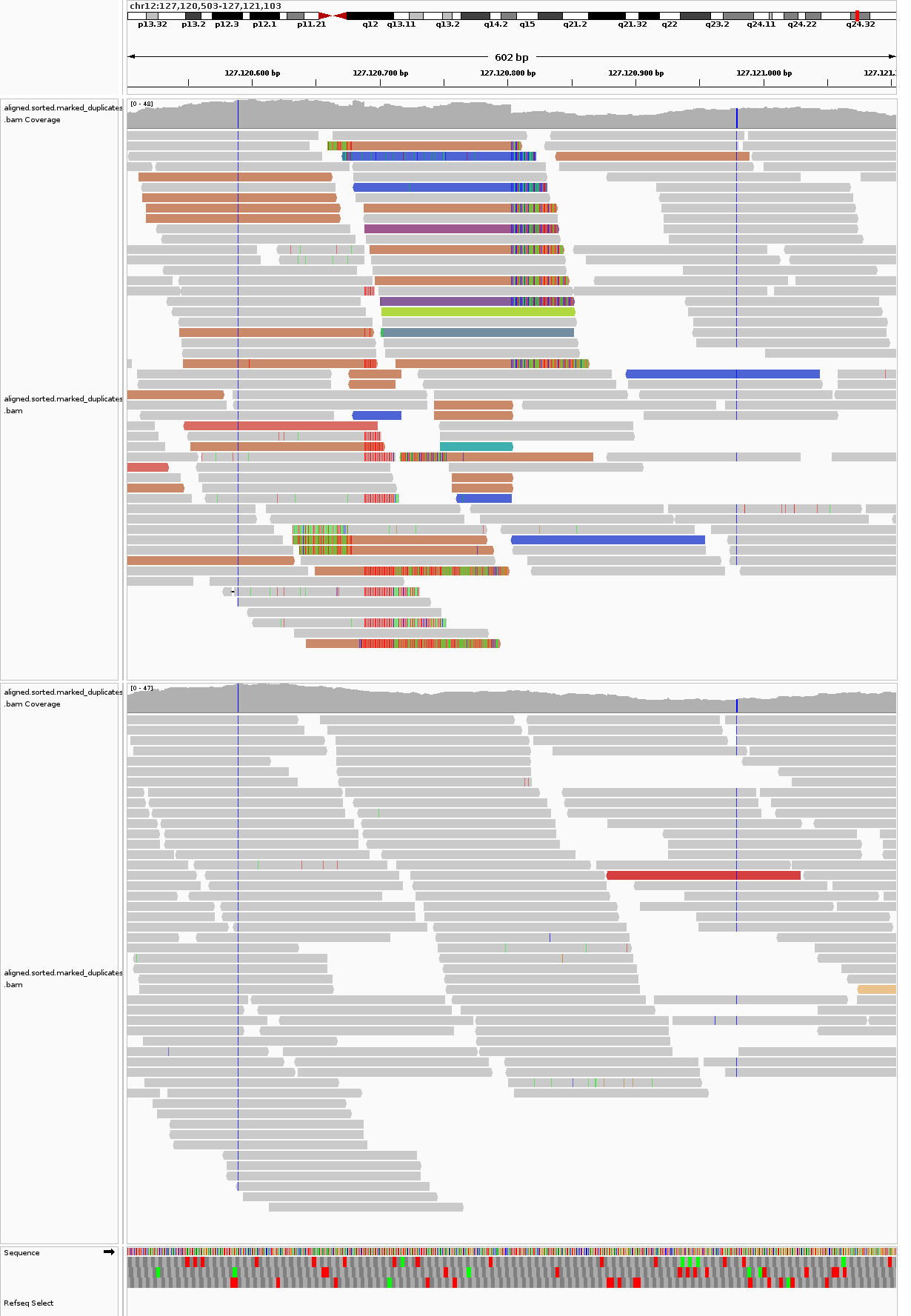

Supplement: Supplementary file 1 — Data S1. Compressed file containing the IGV screenshots for all the RetroTest exclusive insertions inspected in sample_21 and sample_28 WGS data, classified as true positives (TPs), false positives (FPs), and unconclusive. Both the tumor and normal BAM files were included in each screenshot. [file MOL2-19-3769-s003.zip › IGV_screenshots_illuminaWGS_TD2-RetroTest-exclusive_classified/PD0270a_retrotest_exclusive_IlluminaWGS/FPs/chr12_127120503-127121103.png]

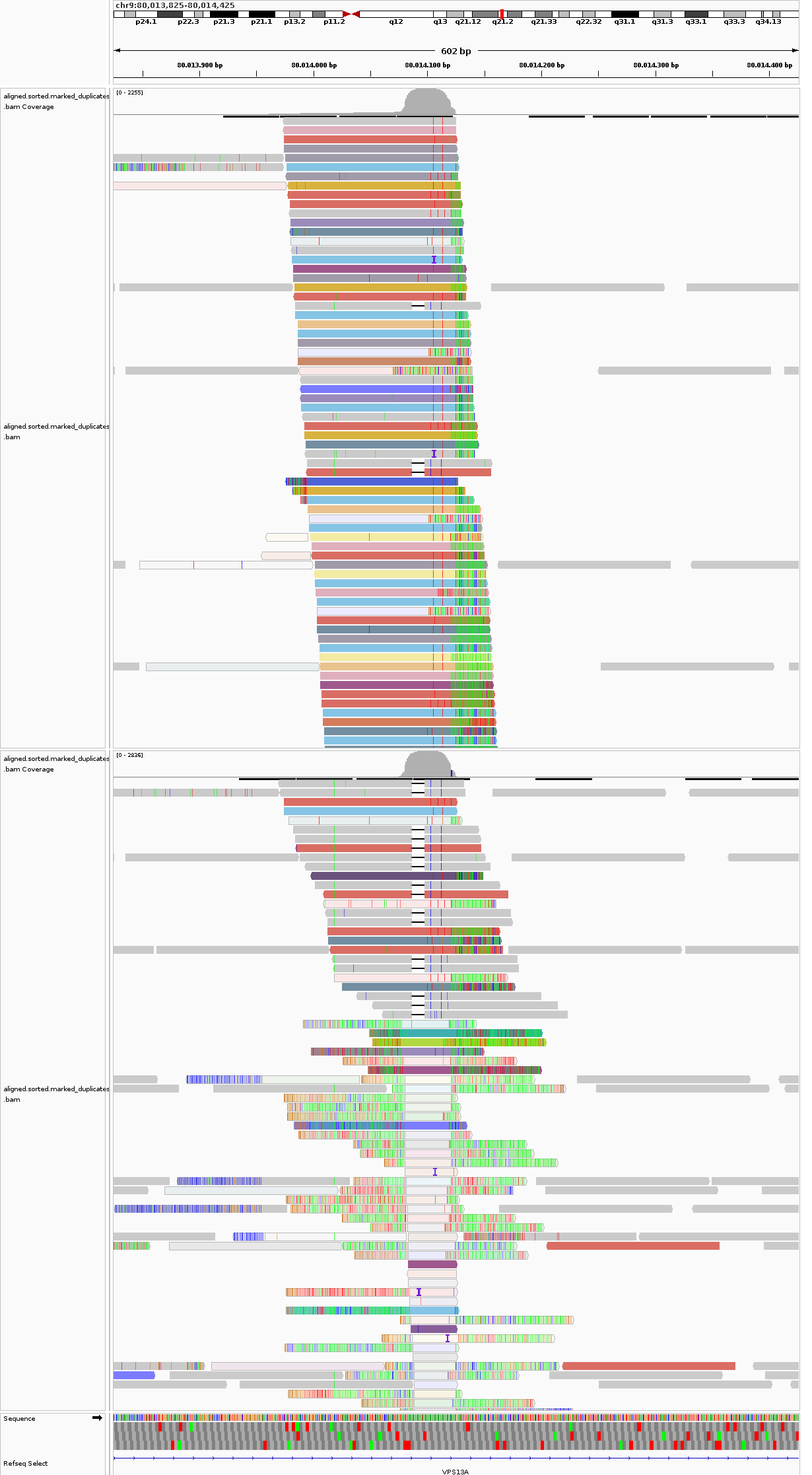

Supplement: Supplementary file 1 — Data S1. Compressed file containing the IGV screenshots for all the RetroTest exclusive insertions inspected in sample_21 and sample_28 WGS data, classified as true positives (TPs), false positives (FPs), and unconclusive. Both the tumor and normal BAM files were included in each screenshot. [file MOL2-19-3769-s003.zip › IGV_screenshots_illuminaWGS_TD2-RetroTest-exclusive_classified/PD0270a_retrotest_exclusive_IlluminaWGS/FPs/chr9_80013825-80014425.png]

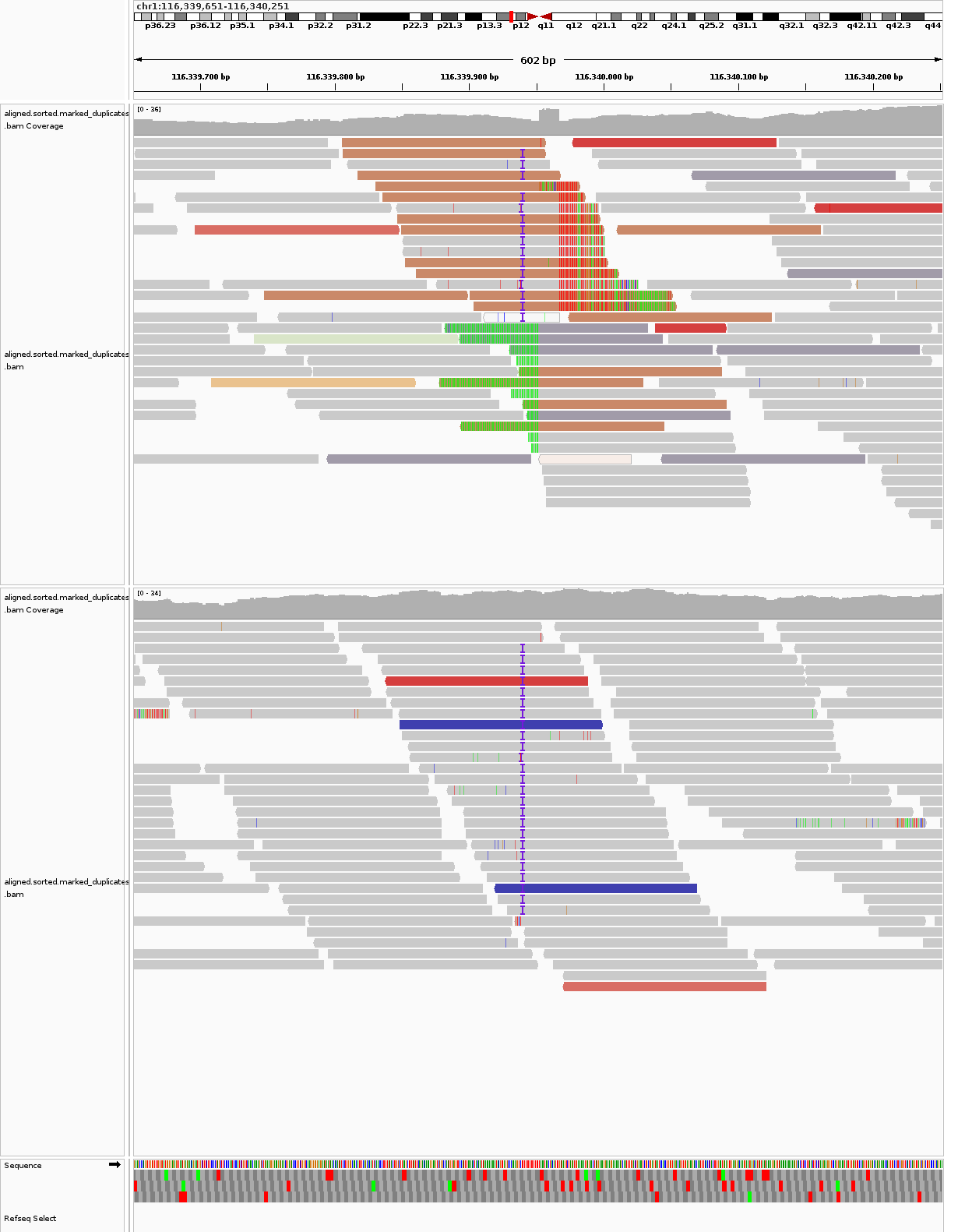

Supplement: Supplementary file 1 — Data S1. Compressed file containing the IGV screenshots for all the RetroTest exclusive insertions inspected in sample_21 and sample_28 WGS data, classified as true positives (TPs), false positives (FPs), and unconclusive. Both the tumor and normal BAM files were included in each screenshot. [file MOL2-19-3769-s003.zip › IGV_screenshots_illuminaWGS_TD2-RetroTest-exclusive_classified/PD0270a_retrotest_exclusive_IlluminaWGS/TPs/chr1_116339651-116340251.png]

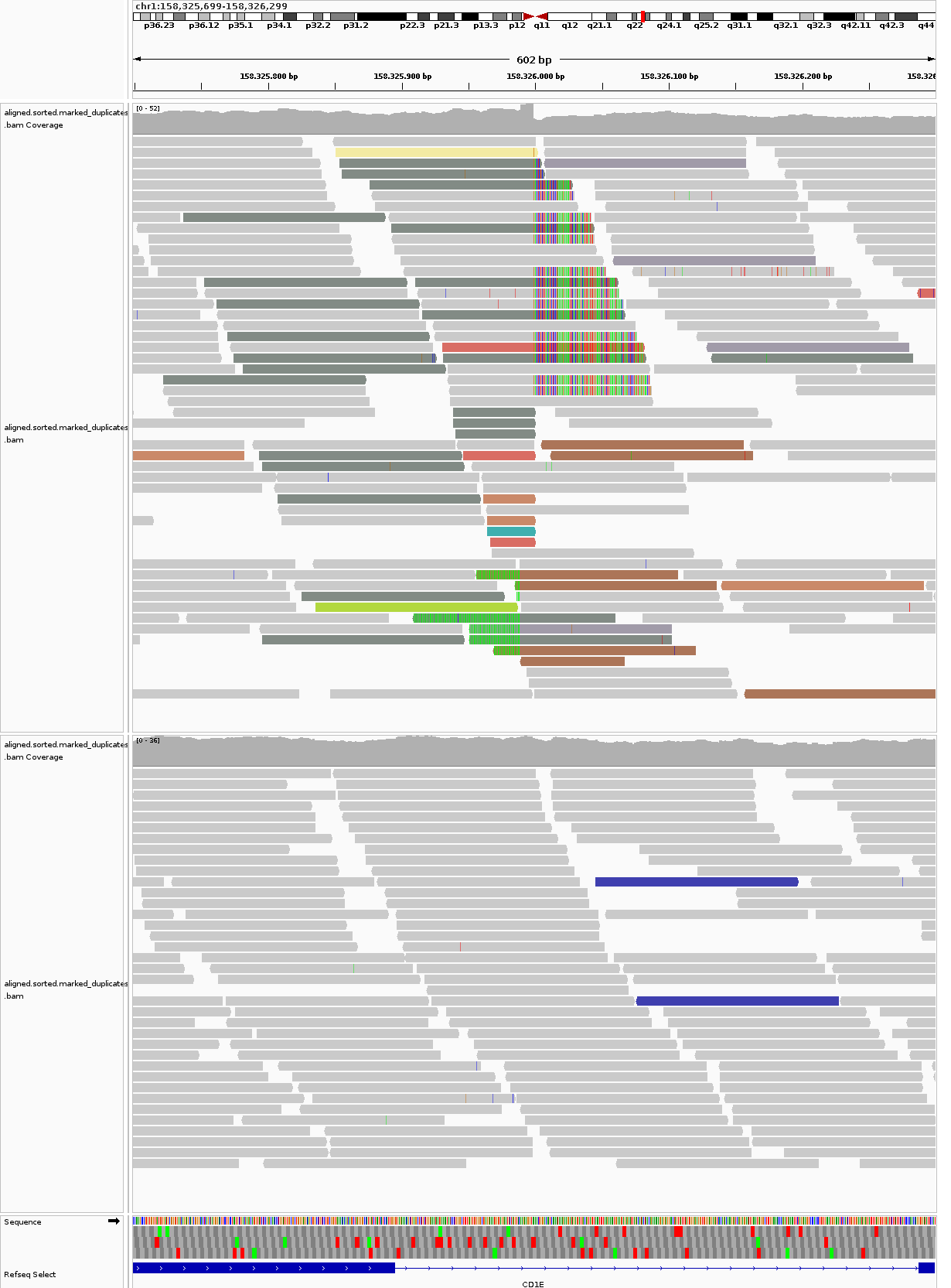

Supplement: Supplementary file 1 — Data S1. Compressed file containing the IGV screenshots for all the RetroTest exclusive insertions inspected in sample_21 and sample_28 WGS data, classified as true positives (TPs), false positives (FPs), and unconclusive. Both the tumor and normal BAM files were included in each screenshot. [file MOL2-19-3769-s003.zip › IGV_screenshots_illuminaWGS_TD2-RetroTest-exclusive_classified/PD0270a_retrotest_exclusive_IlluminaWGS/TPs/chr1_158325699-158326299.png]

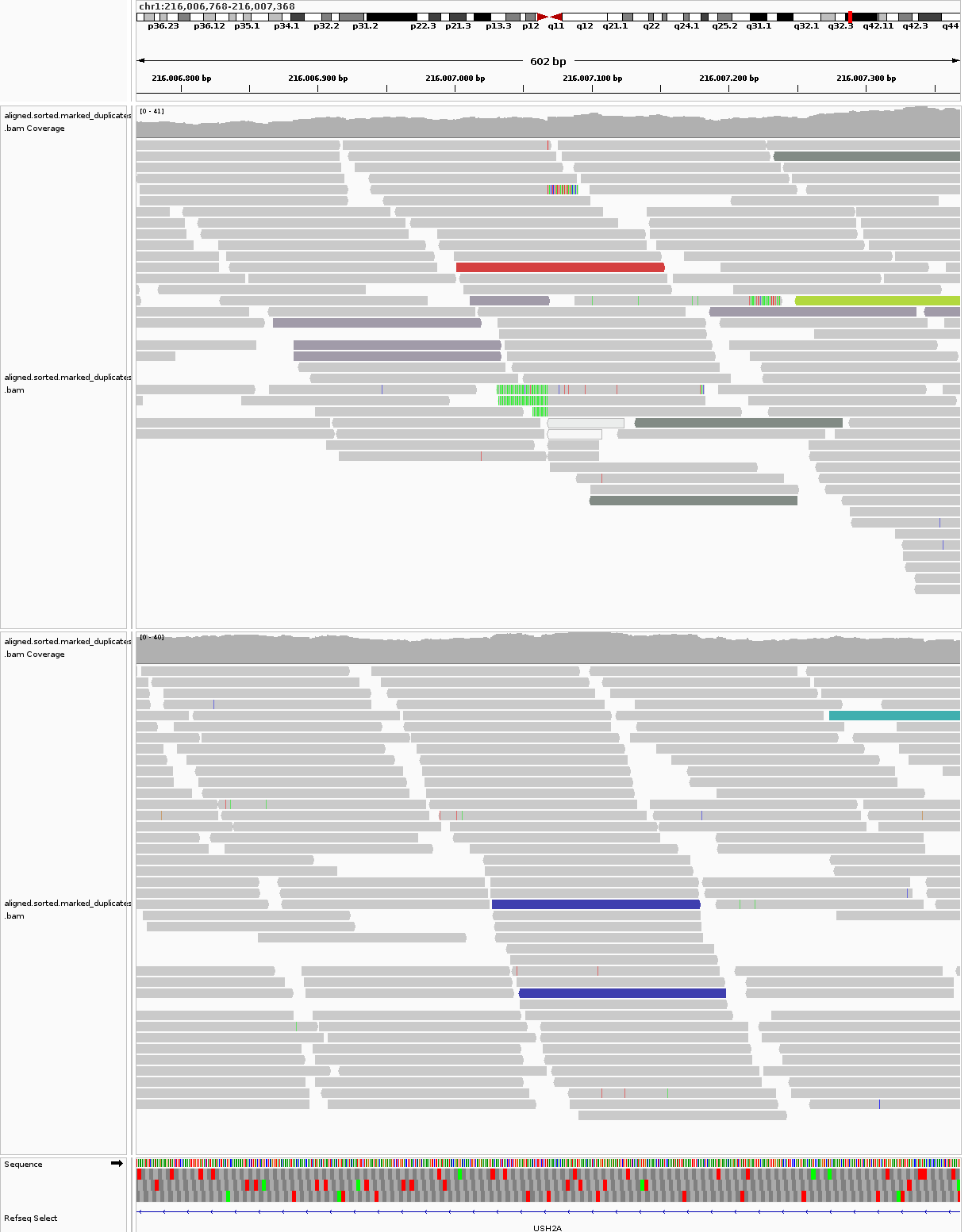

Supplement: Supplementary file 1 — Data S1. Compressed file containing the IGV screenshots for all the RetroTest exclusive insertions inspected in sample_21 and sample_28 WGS data, classified as true positives (TPs), false positives (FPs), and unconclusive. Both the tumor and normal BAM files were included in each screenshot. [file MOL2-19-3769-s003.zip › IGV_screenshots_illuminaWGS_TD2-RetroTest-exclusive_classified/PD0270a_retrotest_exclusive_IlluminaWGS/TPs/chr1_216006768-216007368.png]

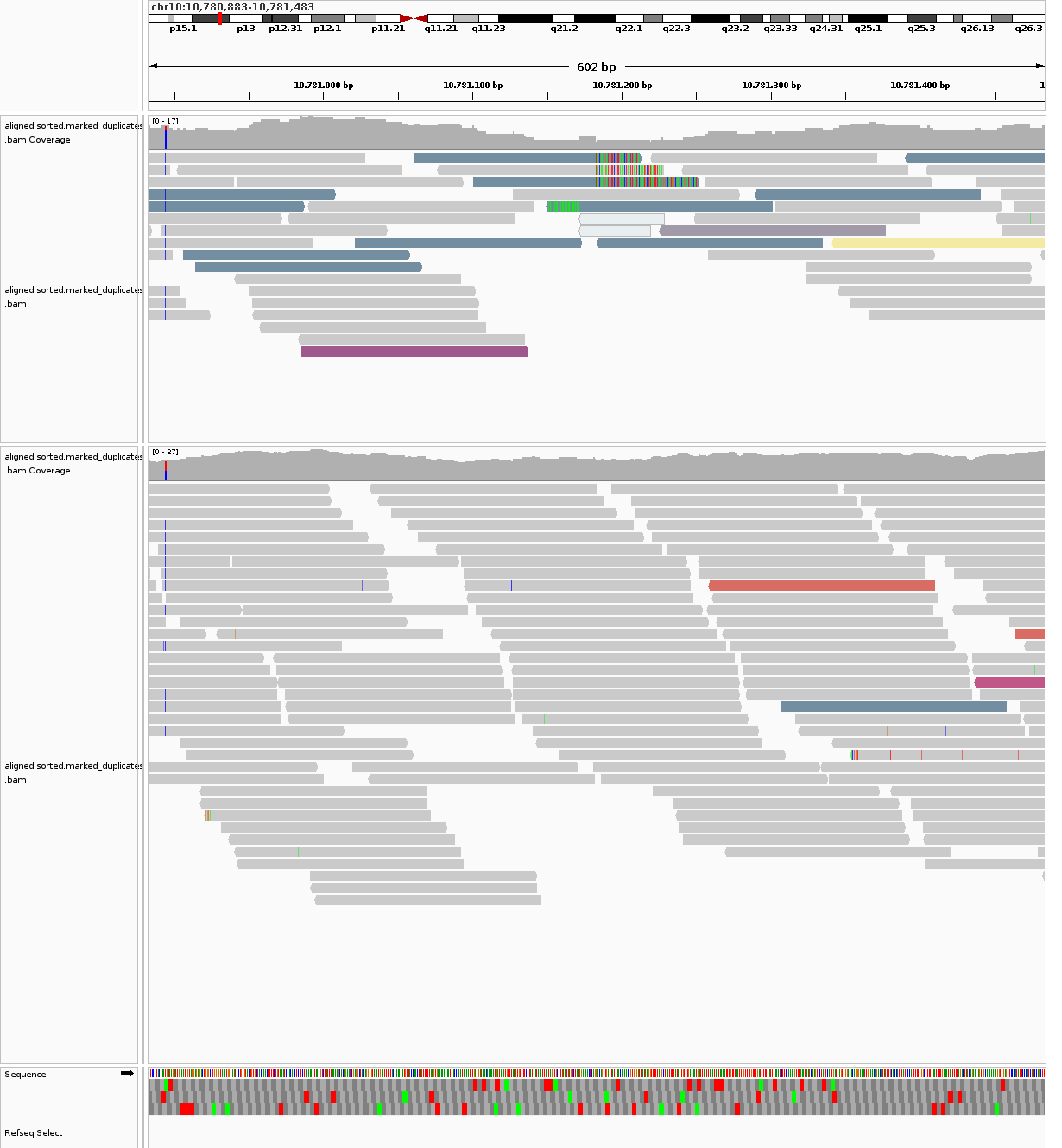

Supplement: Supplementary file 1 — Data S1. Compressed file containing the IGV screenshots for all the RetroTest exclusive insertions inspected in sample_21 and sample_28 WGS data, classified as true positives (TPs), false positives (FPs), and unconclusive. Both the tumor and normal BAM files were included in each screenshot. [file MOL2-19-3769-s003.zip › IGV_screenshots_illuminaWGS_TD2-RetroTest-exclusive_classified/PD0270a_retrotest_exclusive_IlluminaWGS/TPs/chr10_10780883-10781483.png]

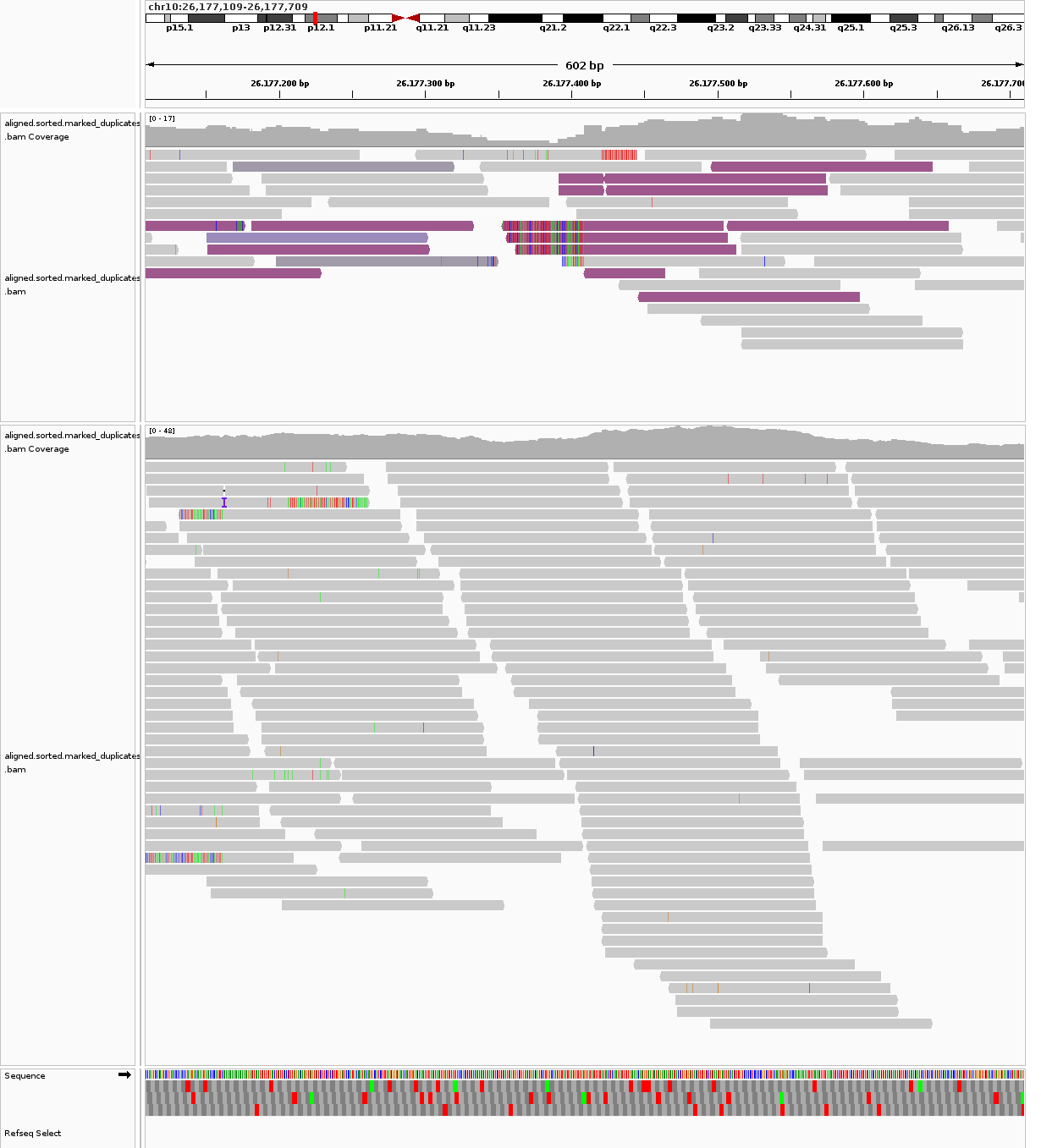

Supplement: Supplementary file 1 — Data S1. Compressed file containing the IGV screenshots for all the RetroTest exclusive insertions inspected in sample_21 and sample_28 WGS data, classified as true positives (TPs), false positives (FPs), and unconclusive. Both the tumor and normal BAM files were included in each screenshot. [file MOL2-19-3769-s003.zip › IGV_screenshots_illuminaWGS_TD2-RetroTest-exclusive_classified/PD0270a_retrotest_exclusive_IlluminaWGS/TPs/chr10_26177109-26177709.png]

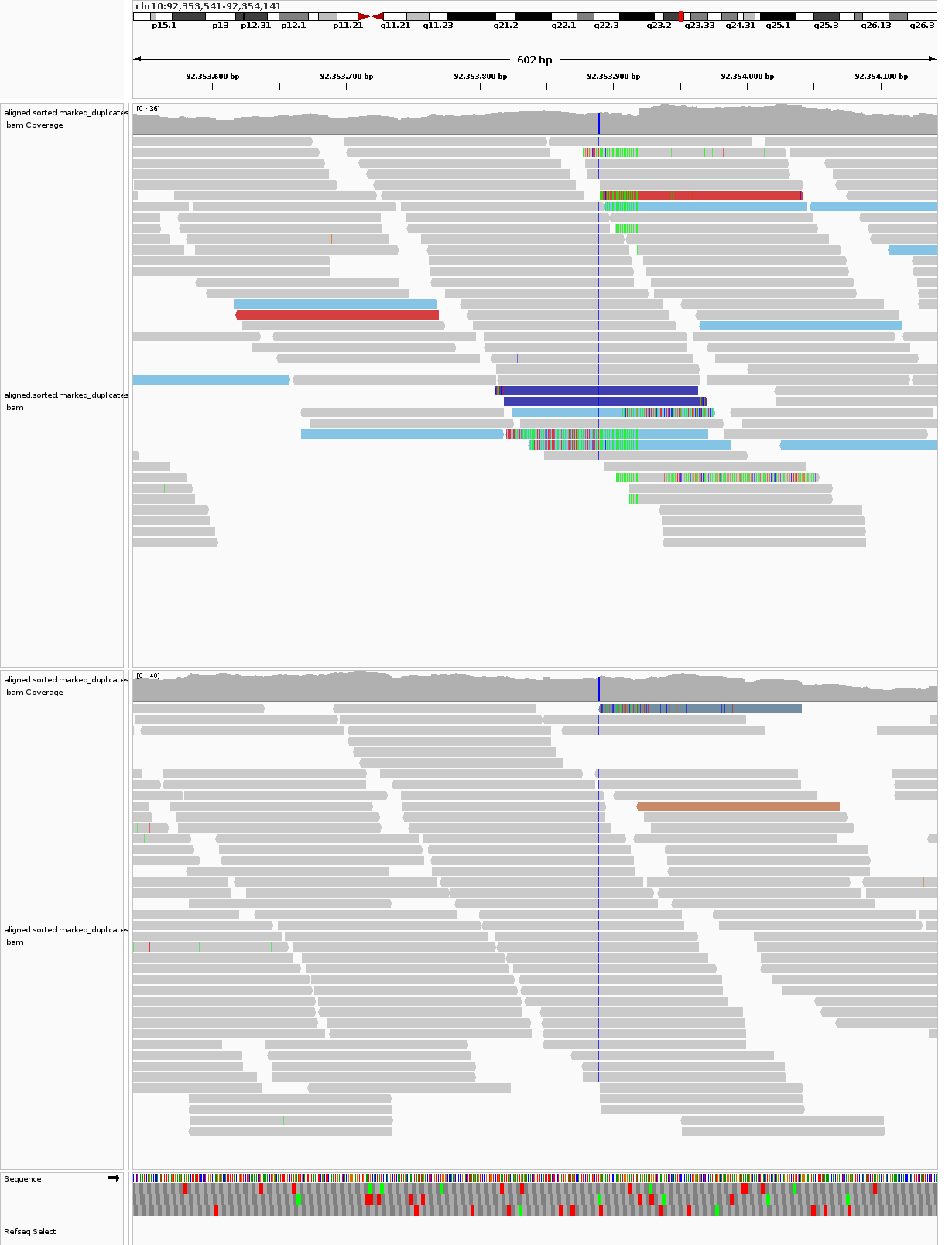

Supplement: Supplementary file 1 — Data S1. Compressed file containing the IGV screenshots for all the RetroTest exclusive insertions inspected in sample_21 and sample_28 WGS data, classified as true positives (TPs), false positives (FPs), and unconclusive. Both the tumor and normal BAM files were included in each screenshot. [file MOL2-19-3769-s003.zip › IGV_screenshots_illuminaWGS_TD2-RetroTest-exclusive_classified/PD0270a_retrotest_exclusive_IlluminaWGS/TPs/chr10_92353541-92354141.png]

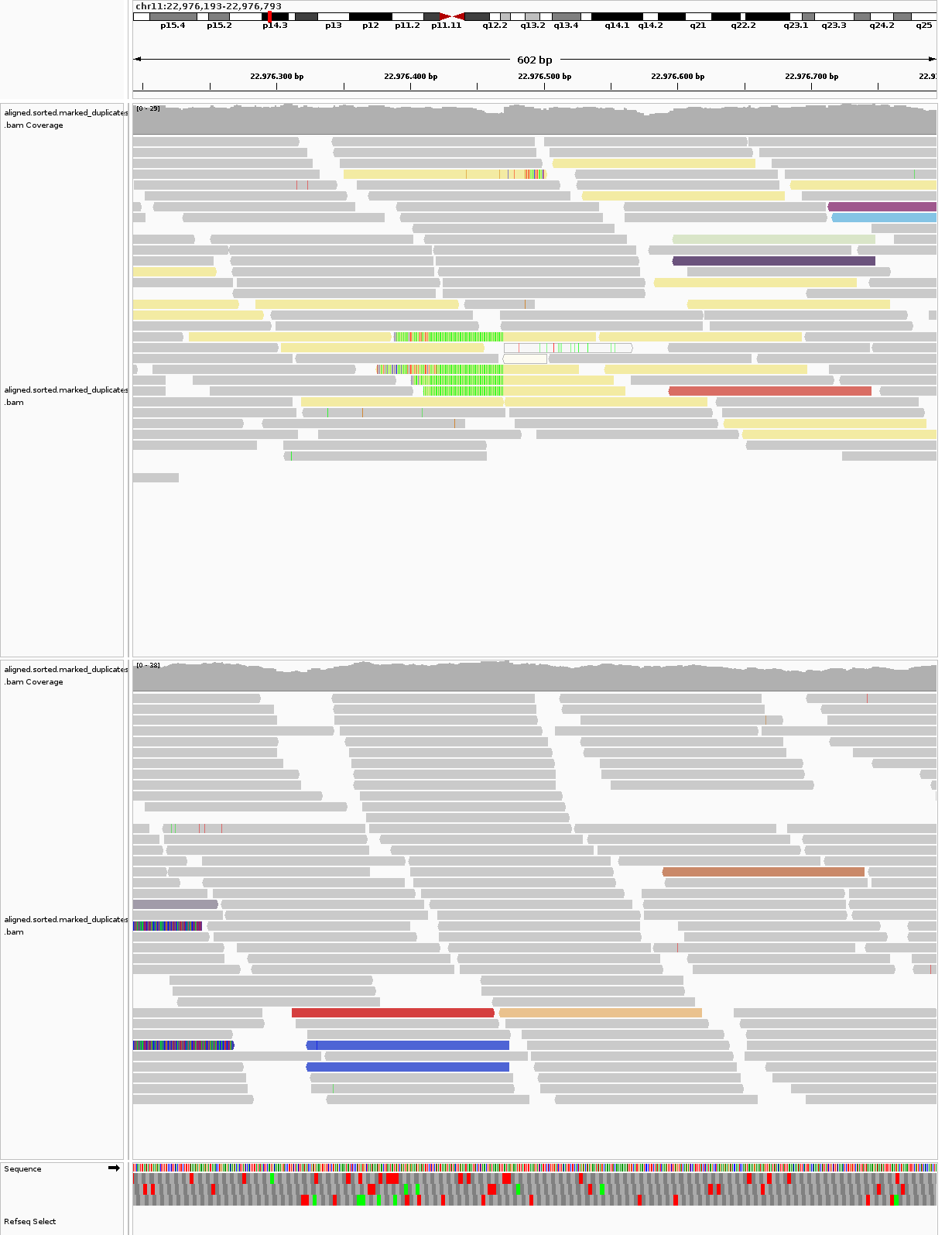

Supplement: Supplementary file 1 — Data S1. Compressed file containing the IGV screenshots for all the RetroTest exclusive insertions inspected in sample_21 and sample_28 WGS data, classified as true positives (TPs), false positives (FPs), and unconclusive. Both the tumor and normal BAM files were included in each screenshot. [file MOL2-19-3769-s003.zip › IGV_screenshots_illuminaWGS_TD2-RetroTest-exclusive_classified/PD0270a_retrotest_exclusive_IlluminaWGS/TPs/chr11_22976193-22976793.png]

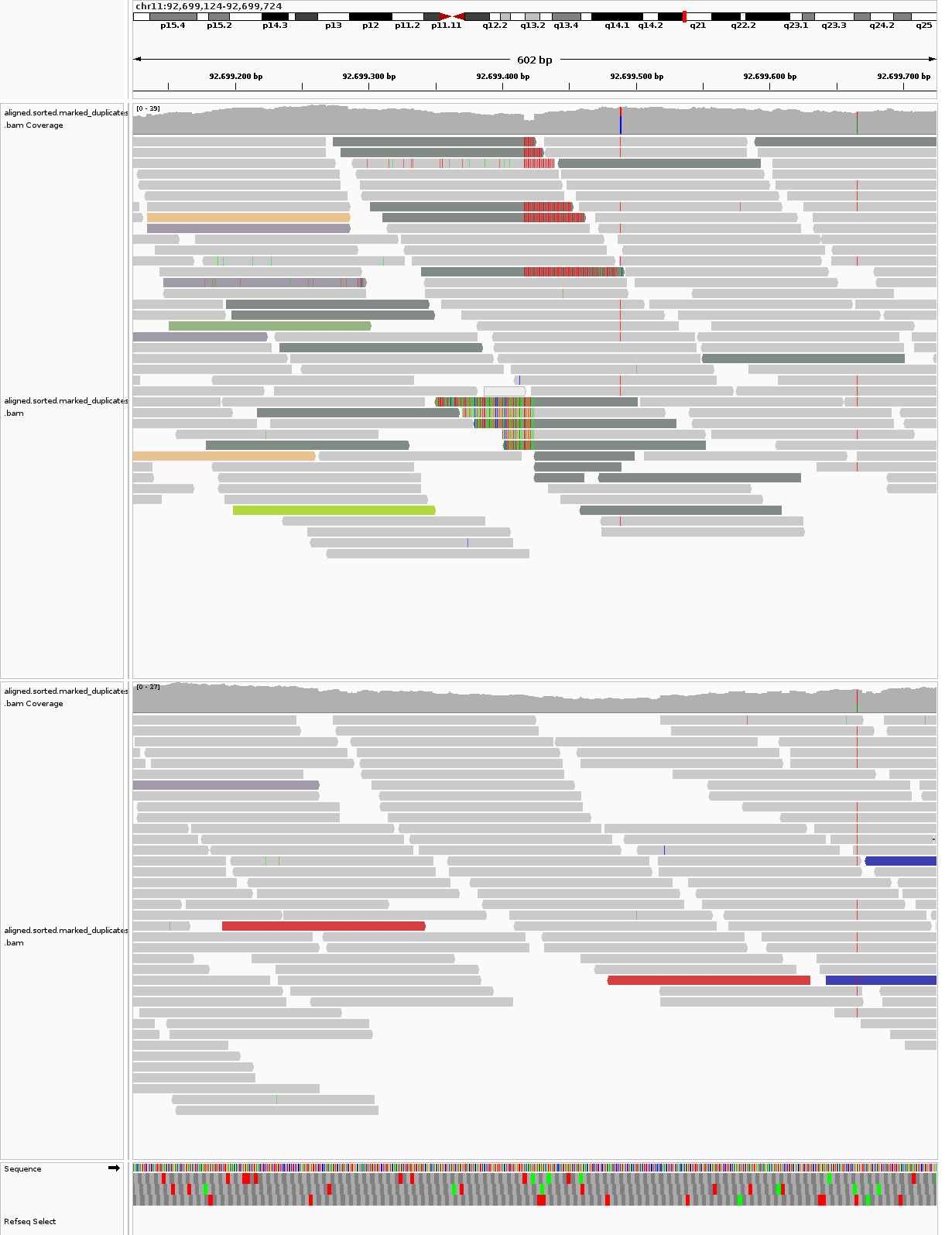

Supplement: Supplementary file 1 — Data S1. Compressed file containing the IGV screenshots for all the RetroTest exclusive insertions inspected in sample_21 and sample_28 WGS data, classified as true positives (TPs), false positives (FPs), and unconclusive. Both the tumor and normal BAM files were included in each screenshot. [file MOL2-19-3769-s003.zip › IGV_screenshots_illuminaWGS_TD2-RetroTest-exclusive_classified/PD0270a_retrotest_exclusive_IlluminaWGS/TPs/chr11_92699124-92699724.png]

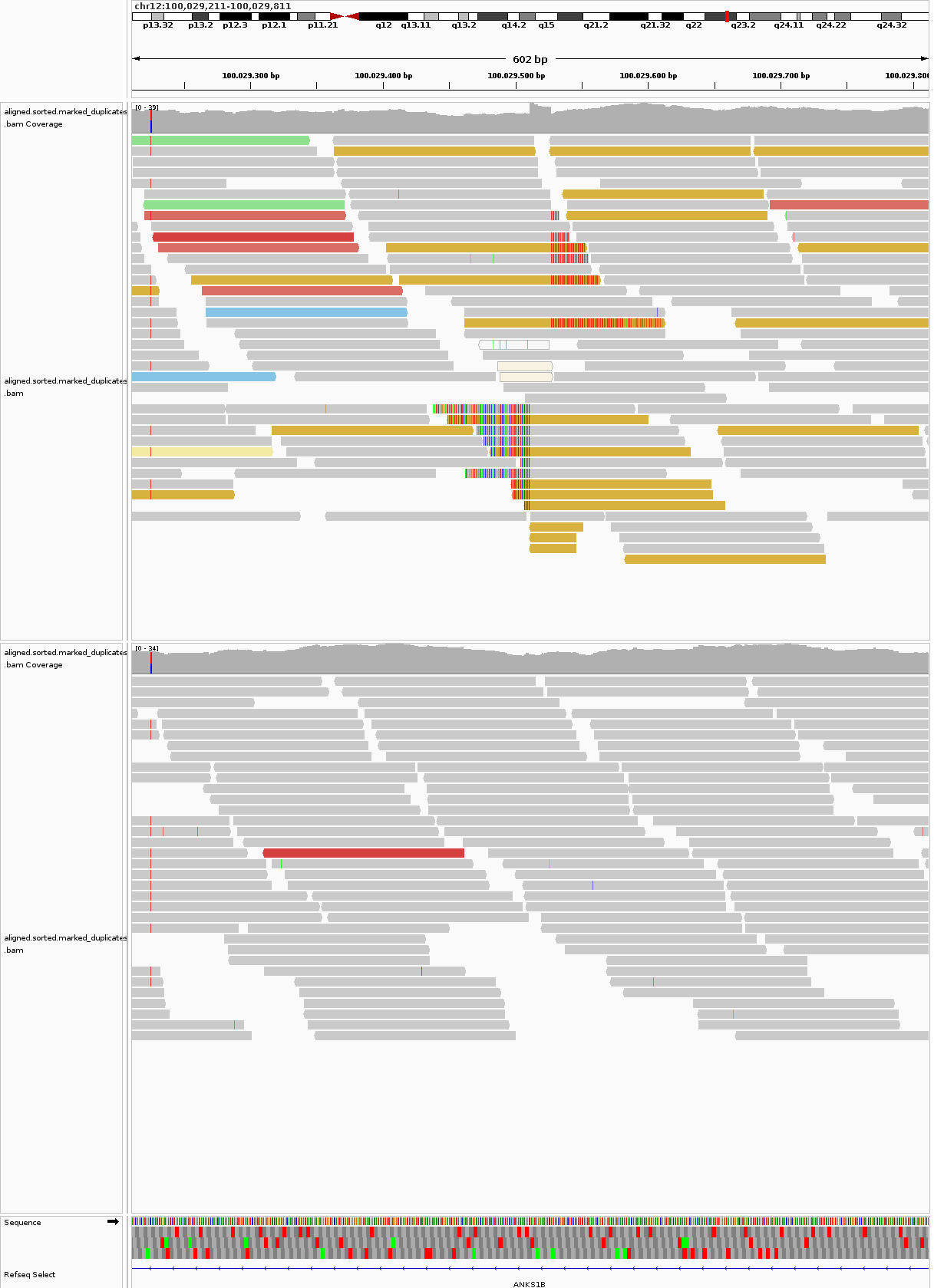

Supplement: Supplementary file 1 — Data S1. Compressed file containing the IGV screenshots for all the RetroTest exclusive insertions inspected in sample_21 and sample_28 WGS data, classified as true positives (TPs), false positives (FPs), and unconclusive. Both the tumor and normal BAM files were included in each screenshot. [file MOL2-19-3769-s003.zip › IGV_screenshots_illuminaWGS_TD2-RetroTest-exclusive_classified/PD0270a_retrotest_exclusive_IlluminaWGS/TPs/chr12_100029211-100029811.png]

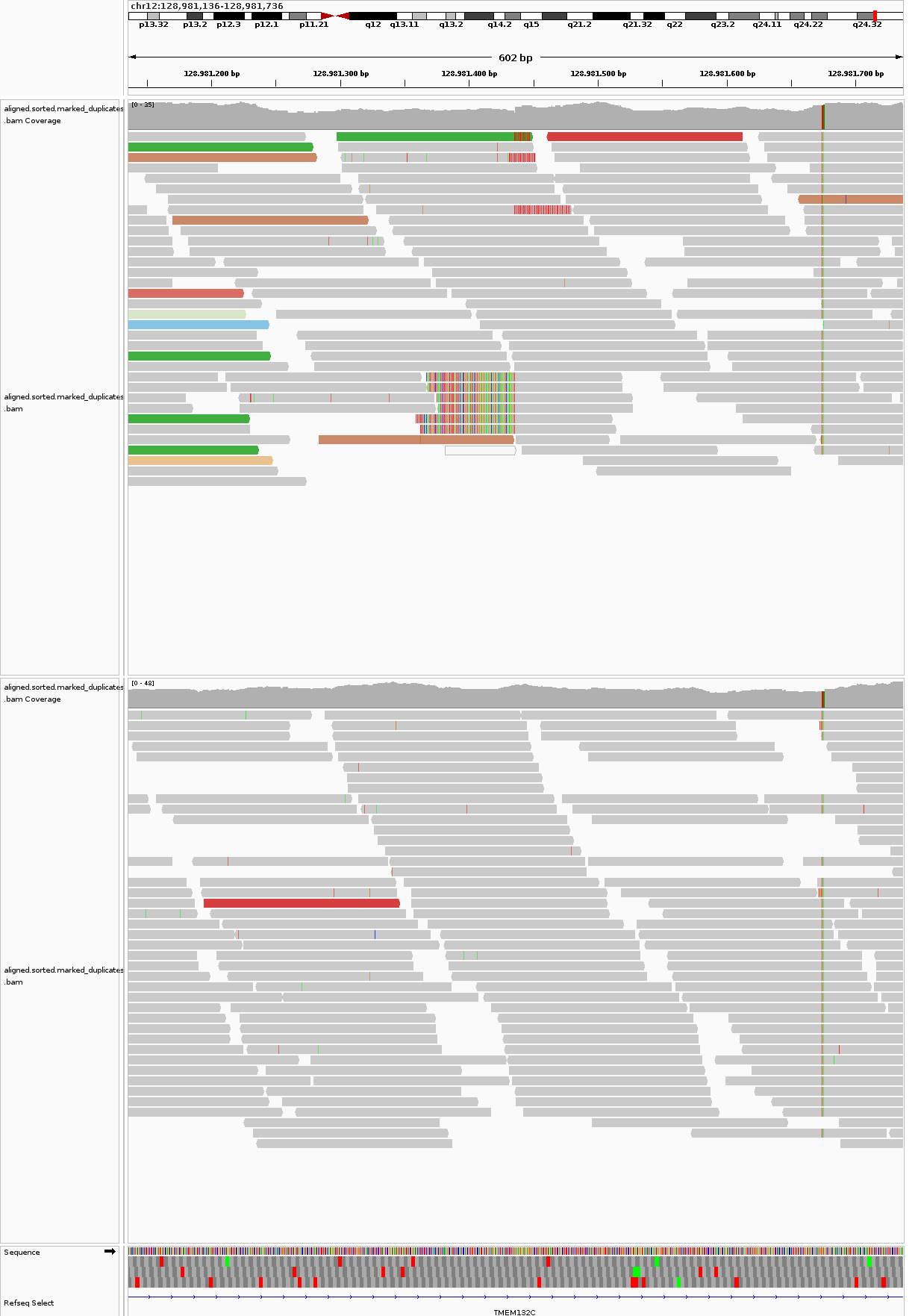

Supplement: Supplementary file 1 — Data S1. Compressed file containing the IGV screenshots for all the RetroTest exclusive insertions inspected in sample_21 and sample_28 WGS data, classified as true positives (TPs), false positives (FPs), and unconclusive. Both the tumor and normal BAM files were included in each screenshot. [file MOL2-19-3769-s003.zip › IGV_screenshots_illuminaWGS_TD2-RetroTest-exclusive_classified/PD0270a_retrotest_exclusive_IlluminaWGS/TPs/chr12_128981136-128981736.png]

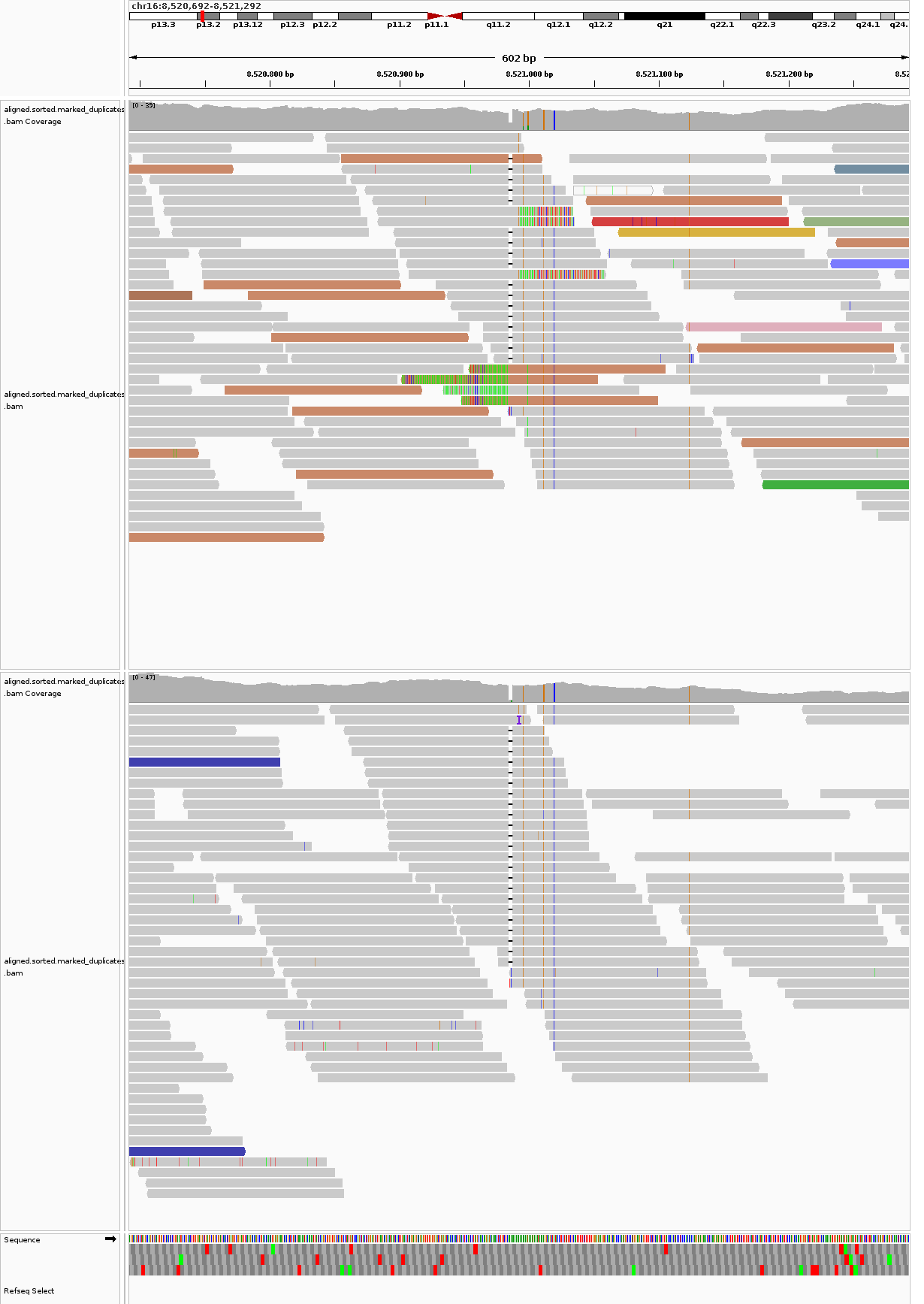

Supplement: Supplementary file 1 — Data S1. Compressed file containing the IGV screenshots for all the RetroTest exclusive insertions inspected in sample_21 and sample_28 WGS data, classified as true positives (TPs), false positives (FPs), and unconclusive. Both the tumor and normal BAM files were included in each screenshot. [file MOL2-19-3769-s003.zip › IGV_screenshots_illuminaWGS_TD2-RetroTest-exclusive_classified/PD0270a_retrotest_exclusive_IlluminaWGS/TPs/chr16_8520692-8521292.png]

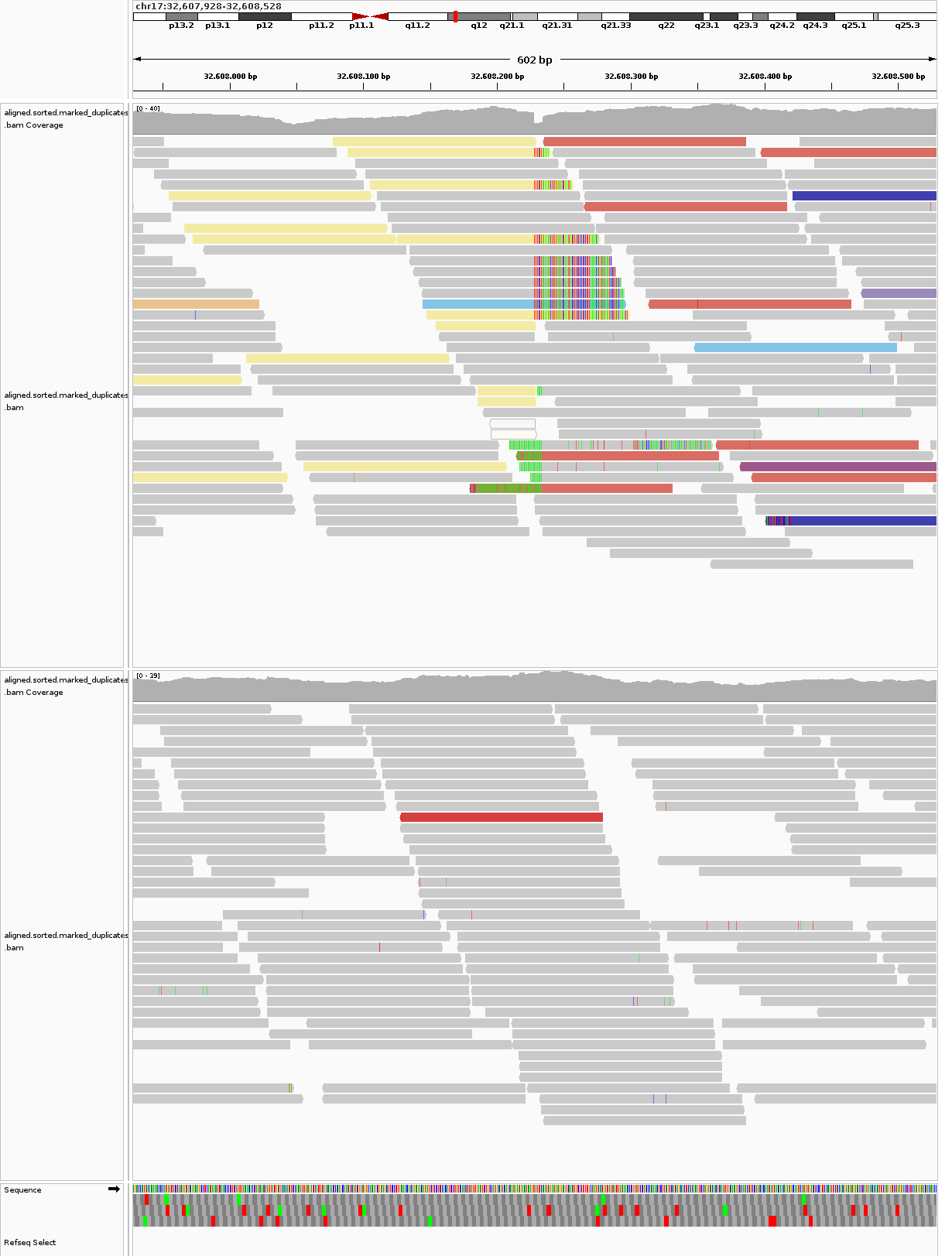

Supplement: Supplementary file 1 — Data S1. Compressed file containing the IGV screenshots for all the RetroTest exclusive insertions inspected in sample_21 and sample_28 WGS data, classified as true positives (TPs), false positives (FPs), and unconclusive. Both the tumor and normal BAM files were included in each screenshot. [file MOL2-19-3769-s003.zip › IGV_screenshots_illuminaWGS_TD2-RetroTest-exclusive_classified/PD0270a_retrotest_exclusive_IlluminaWGS/TPs/chr17_32607928-32608528.png]

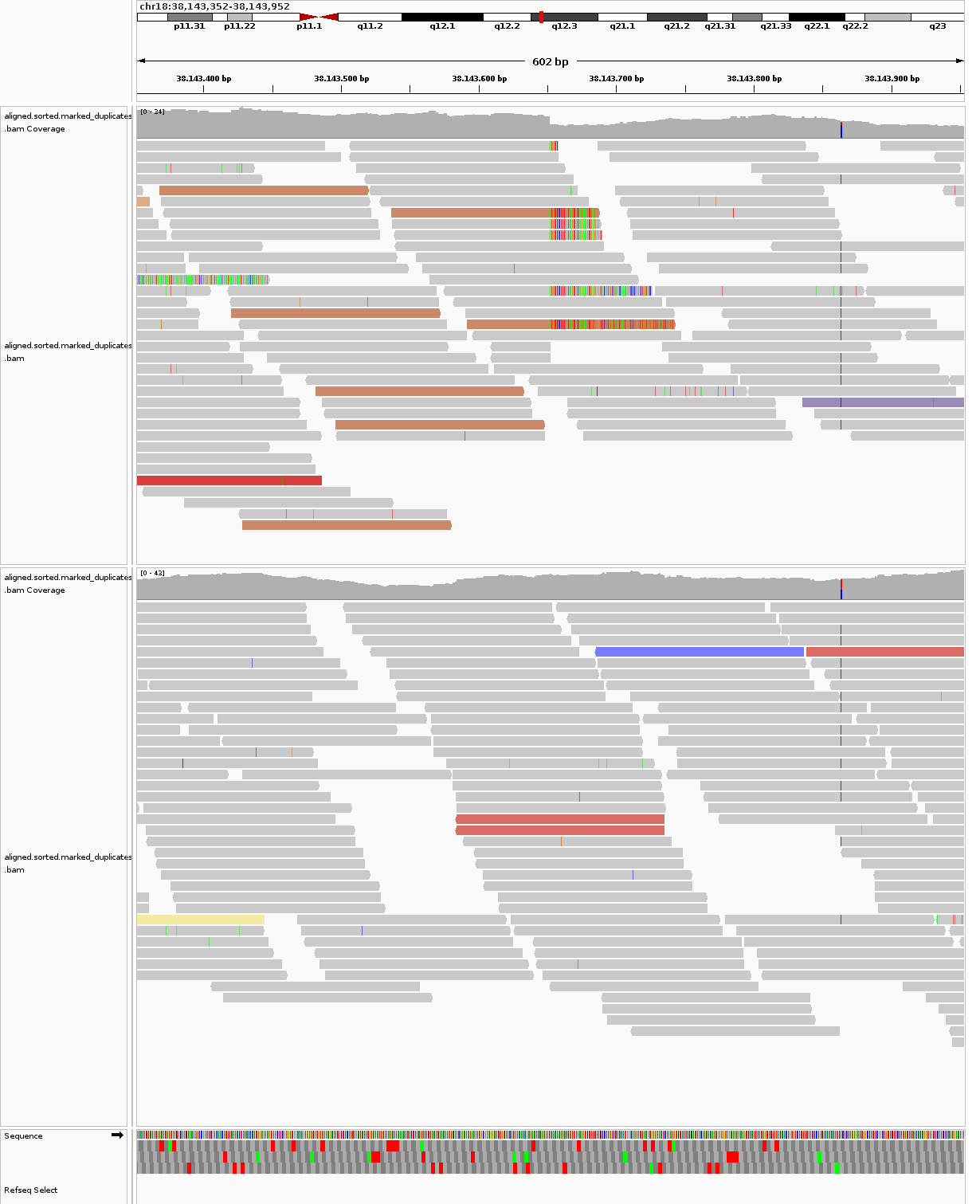

Supplement: Supplementary file 1 — Data S1. Compressed file containing the IGV screenshots for all the RetroTest exclusive insertions inspected in sample_21 and sample_28 WGS data, classified as true positives (TPs), false positives (FPs), and unconclusive. Both the tumor and normal BAM files were included in each screenshot. [file MOL2-19-3769-s003.zip › IGV_screenshots_illuminaWGS_TD2-RetroTest-exclusive_classified/PD0270a_retrotest_exclusive_IlluminaWGS/TPs/chr18_38143352-38143952.png]

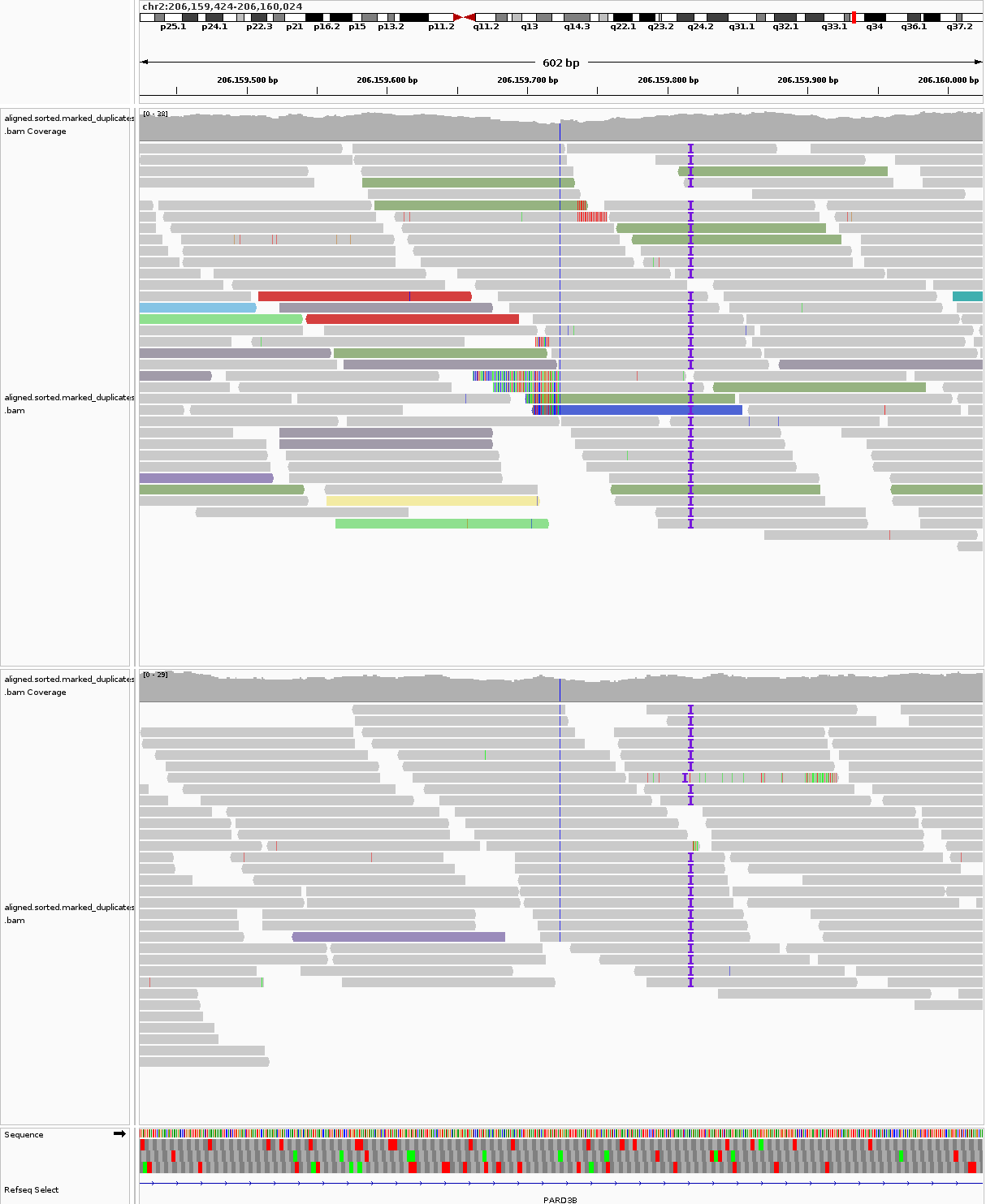

Supplement: Supplementary file 1 — Data S1. Compressed file containing the IGV screenshots for all the RetroTest exclusive insertions inspected in sample_21 and sample_28 WGS data, classified as true positives (TPs), false positives (FPs), and unconclusive. Both the tumor and normal BAM files were included in each screenshot. [file MOL2-19-3769-s003.zip › IGV_screenshots_illuminaWGS_TD2-RetroTest-exclusive_classified/PD0270a_retrotest_exclusive_IlluminaWGS/TPs/chr2_206159424-206160024.png]

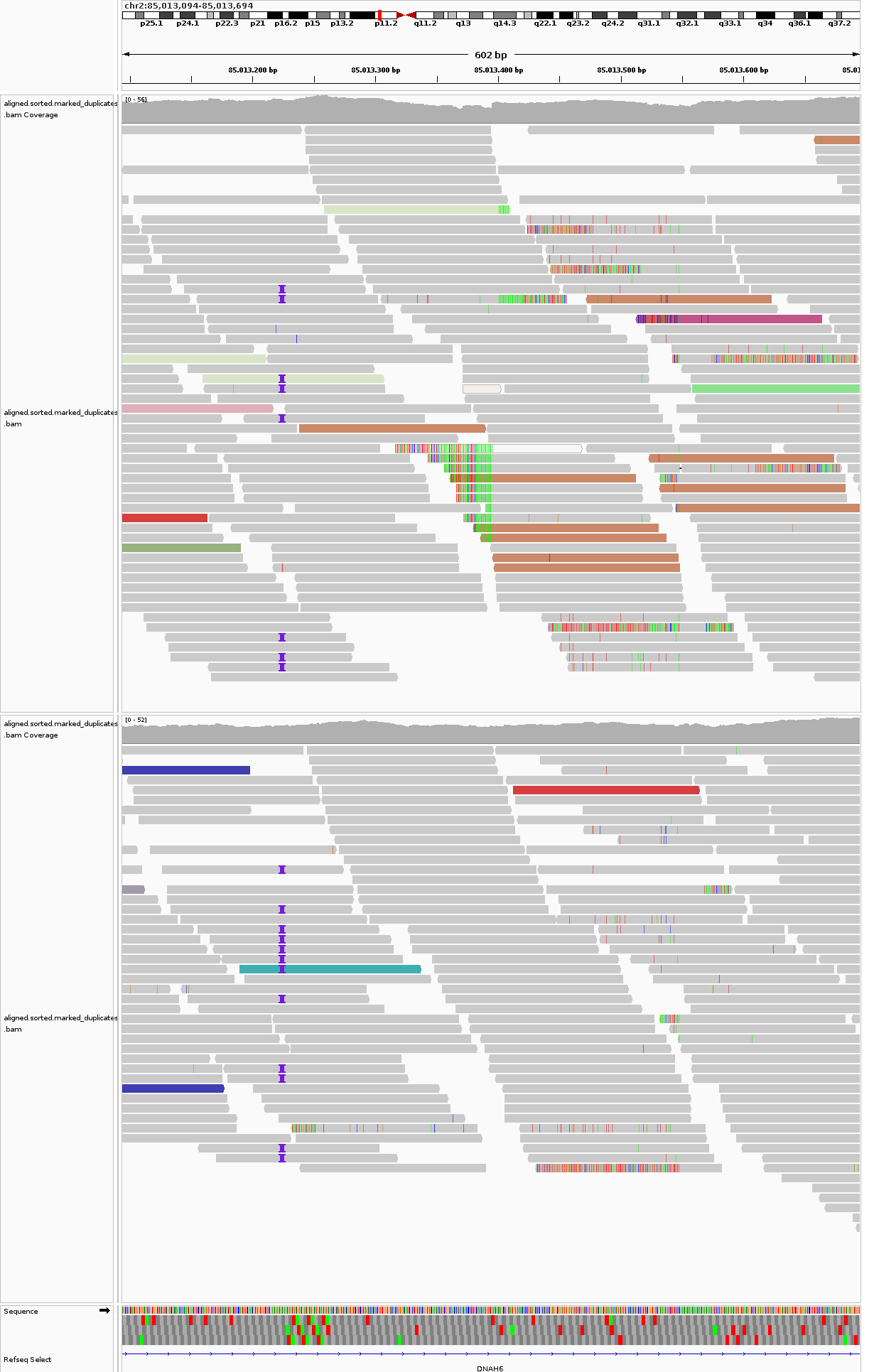

Supplement: Supplementary file 1 — Data S1. Compressed file containing the IGV screenshots for all the RetroTest exclusive insertions inspected in sample_21 and sample_28 WGS data, classified as true positives (TPs), false positives (FPs), and unconclusive. Both the tumor and normal BAM files were included in each screenshot. [file MOL2-19-3769-s003.zip › IGV_screenshots_illuminaWGS_TD2-RetroTest-exclusive_classified/PD0270a_retrotest_exclusive_IlluminaWGS/TPs/chr2_85013094-85013694.png]

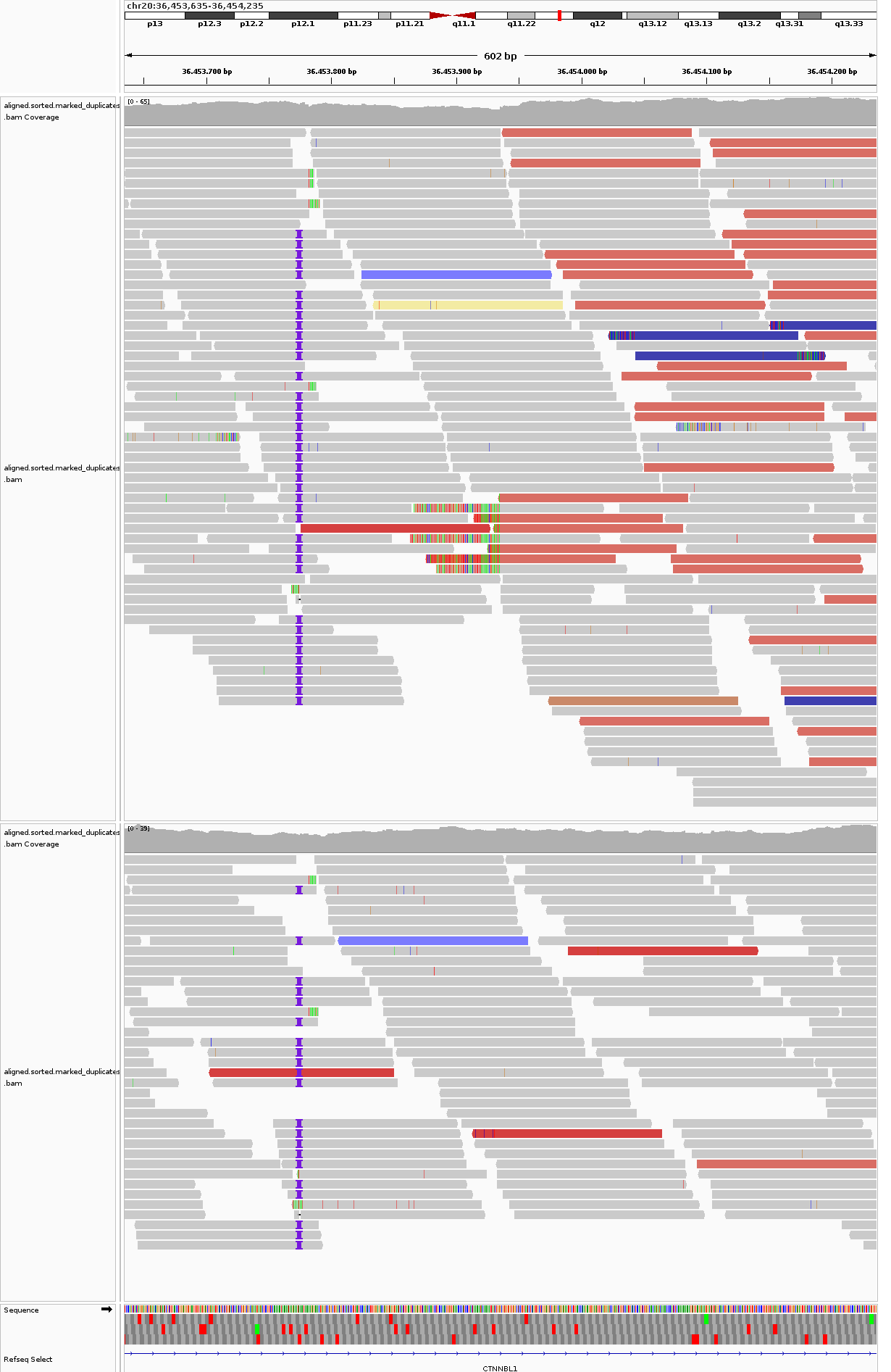

Supplement: Supplementary file 1 — Data S1. Compressed file containing the IGV screenshots for all the RetroTest exclusive insertions inspected in sample_21 and sample_28 WGS data, classified as true positives (TPs), false positives (FPs), and unconclusive. Both the tumor and normal BAM files were included in each screenshot. [file MOL2-19-3769-s003.zip › IGV_screenshots_illuminaWGS_TD2-RetroTest-exclusive_classified/PD0270a_retrotest_exclusive_IlluminaWGS/TPs/chr20_36453635-36454235.png]

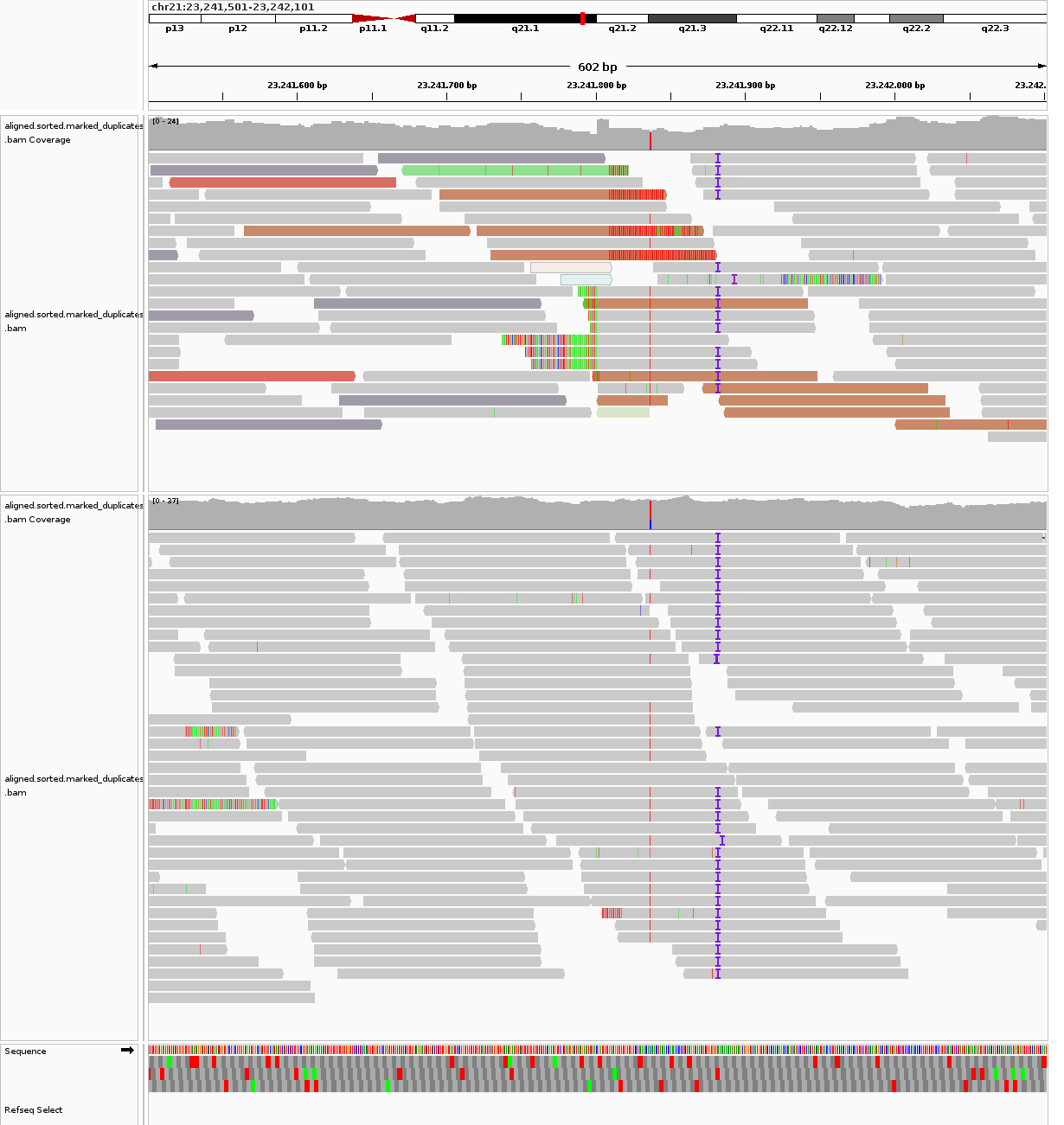

Supplement: Supplementary file 1 — Data S1. Compressed file containing the IGV screenshots for all the RetroTest exclusive insertions inspected in sample_21 and sample_28 WGS data, classified as true positives (TPs), false positives (FPs), and unconclusive. Both the tumor and normal BAM files were included in each screenshot. [file MOL2-19-3769-s003.zip › IGV_screenshots_illuminaWGS_TD2-RetroTest-exclusive_classified/PD0270a_retrotest_exclusive_IlluminaWGS/TPs/chr21_23241501-23242101.png]

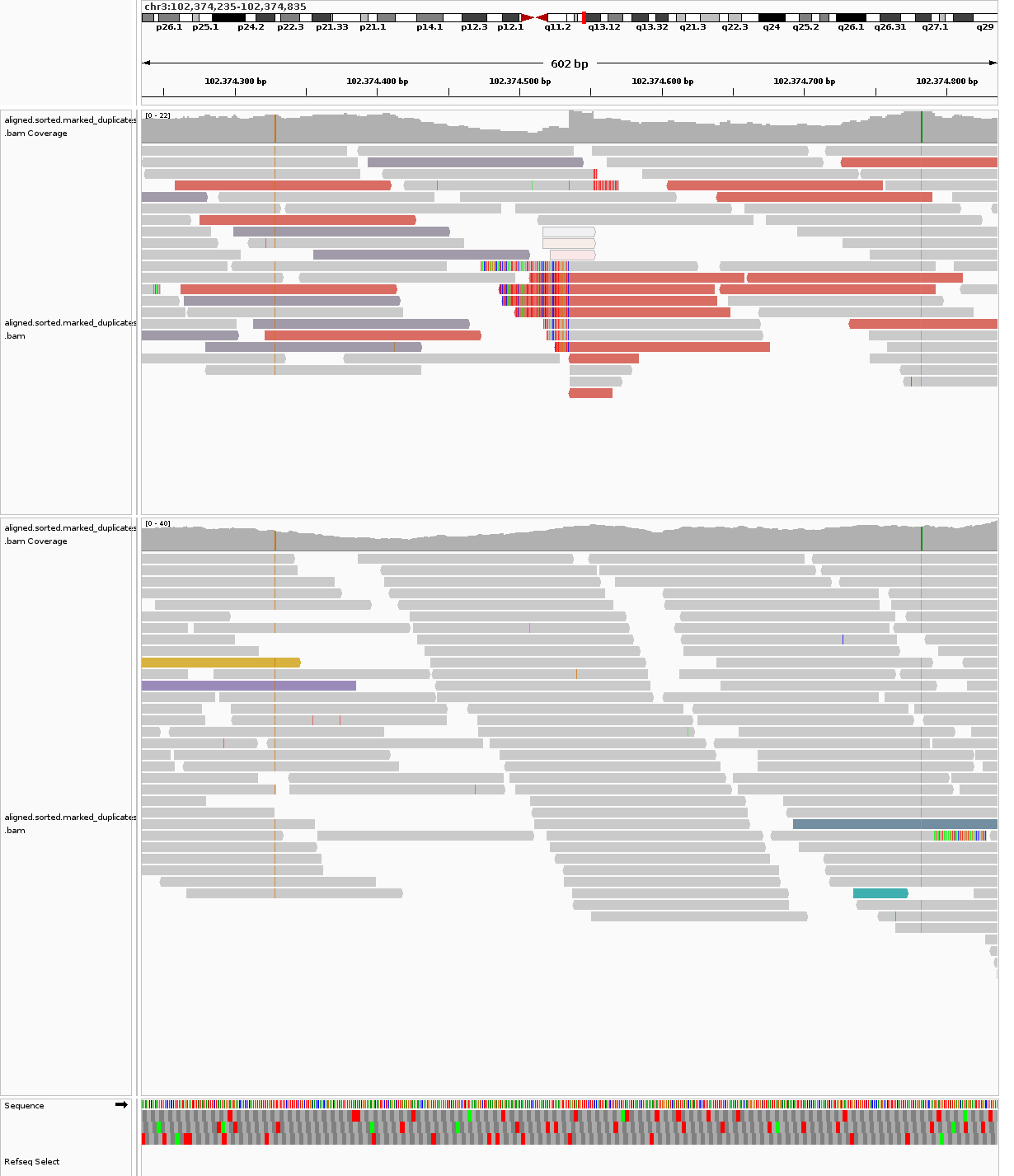

Supplement: Supplementary file 1 — Data S1. Compressed file containing the IGV screenshots for all the RetroTest exclusive insertions inspected in sample_21 and sample_28 WGS data, classified as true positives (TPs), false positives (FPs), and unconclusive. Both the tumor and normal BAM files were included in each screenshot. [file MOL2-19-3769-s003.zip › IGV_screenshots_illuminaWGS_TD2-RetroTest-exclusive_classified/PD0270a_retrotest_exclusive_IlluminaWGS/TPs/chr3_102374235-102374835.png]

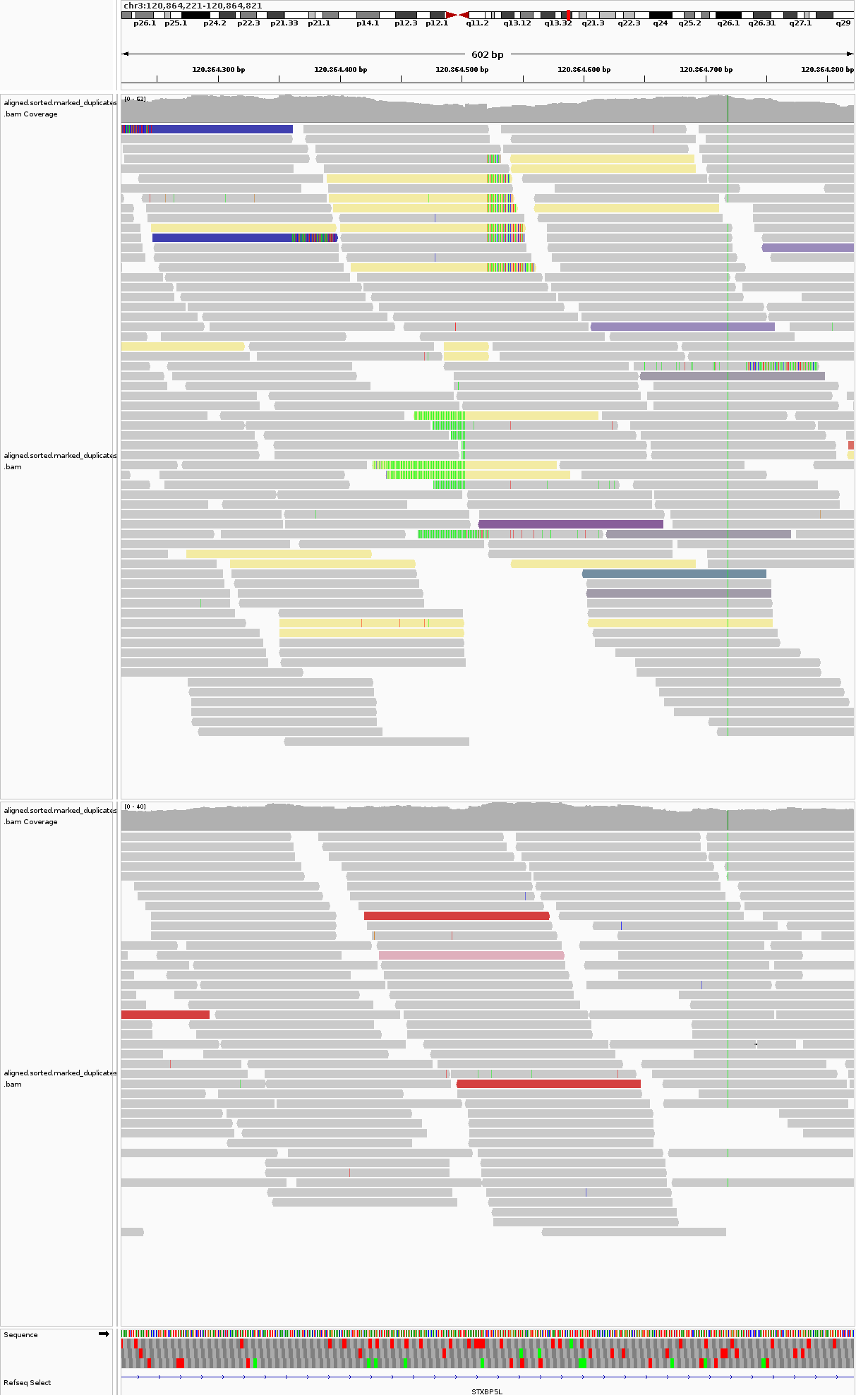

Supplement: Supplementary file 1 — Data S1. Compressed file containing the IGV screenshots for all the RetroTest exclusive insertions inspected in sample_21 and sample_28 WGS data, classified as true positives (TPs), false positives (FPs), and unconclusive. Both the tumor and normal BAM files were included in each screenshot. [file MOL2-19-3769-s003.zip › IGV_screenshots_illuminaWGS_TD2-RetroTest-exclusive_classified/PD0270a_retrotest_exclusive_IlluminaWGS/TPs/chr3_120864221-120864821.png]

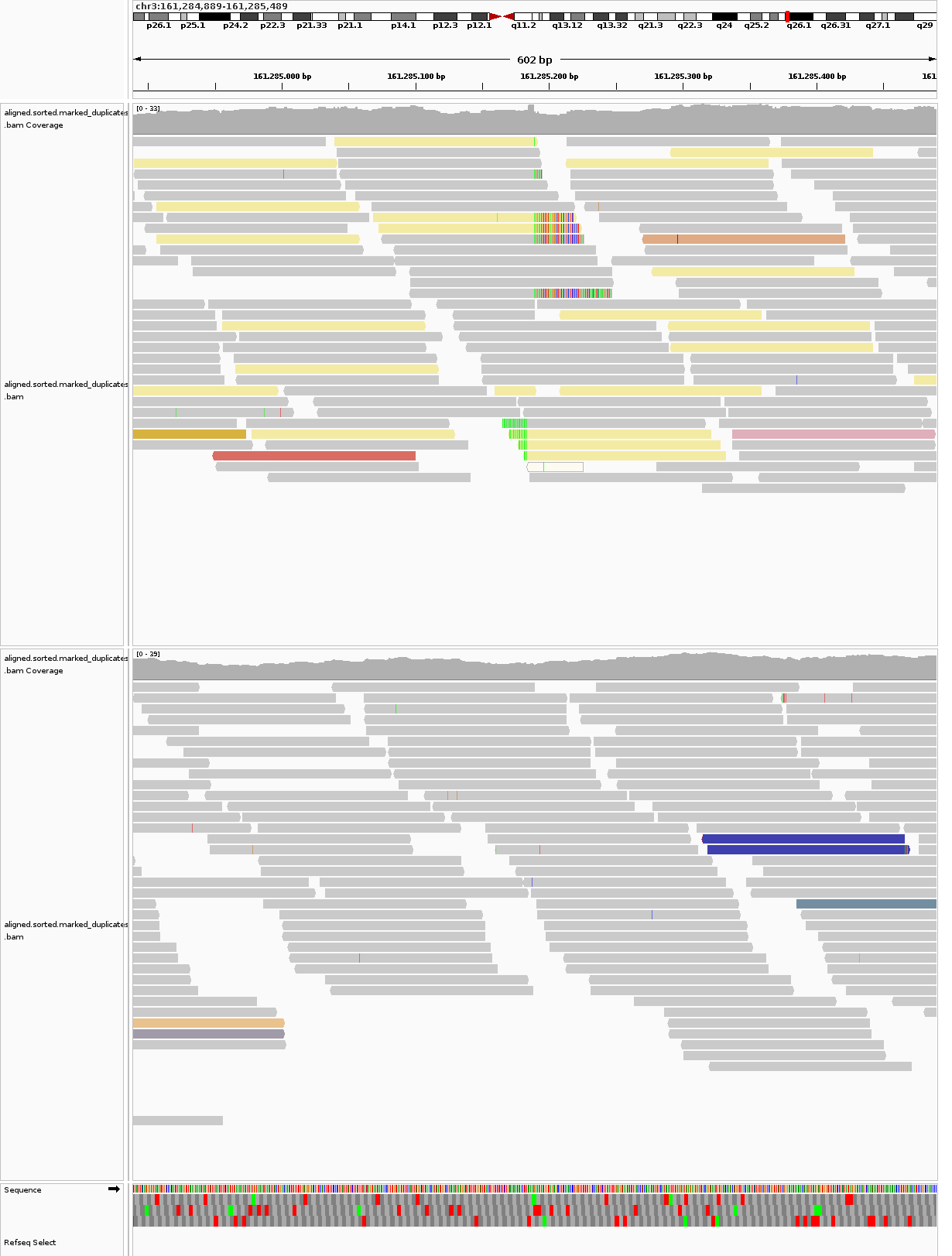

Supplement: Supplementary file 1 — Data S1. Compressed file containing the IGV screenshots for all the RetroTest exclusive insertions inspected in sample_21 and sample_28 WGS data, classified as true positives (TPs), false positives (FPs), and unconclusive. Both the tumor and normal BAM files were included in each screenshot. [file MOL2-19-3769-s003.zip › IGV_screenshots_illuminaWGS_TD2-RetroTest-exclusive_classified/PD0270a_retrotest_exclusive_IlluminaWGS/TPs/chr3_161284889-161285489.png]

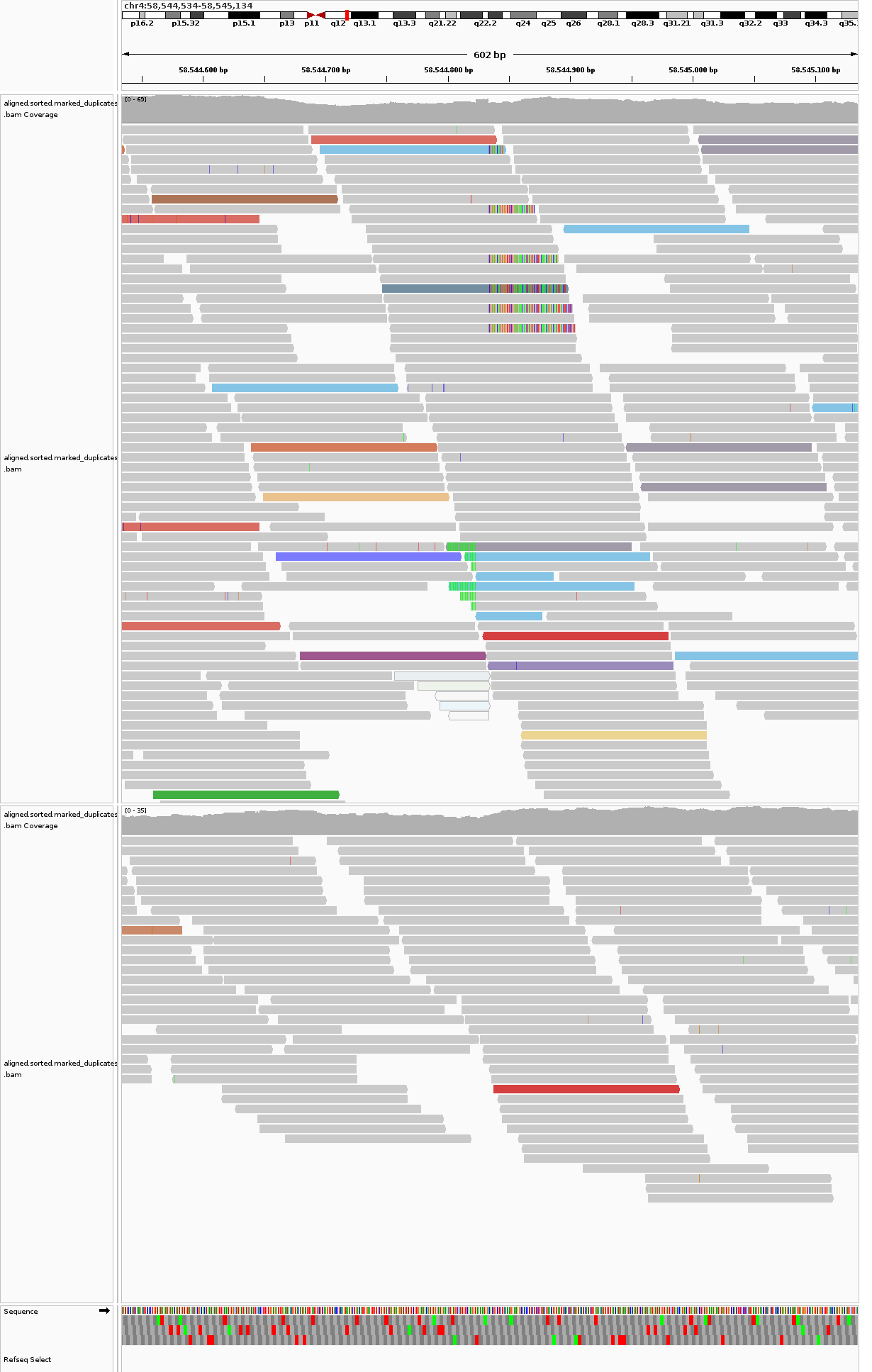

Supplement: Supplementary file 1 — Data S1. Compressed file containing the IGV screenshots for all the RetroTest exclusive insertions inspected in sample_21 and sample_28 WGS data, classified as true positives (TPs), false positives (FPs), and unconclusive. Both the tumor and normal BAM files were included in each screenshot. [file MOL2-19-3769-s003.zip › IGV_screenshots_illuminaWGS_TD2-RetroTest-exclusive_classified/PD0270a_retrotest_exclusive_IlluminaWGS/TPs/chr4_58544534-58545134.png]

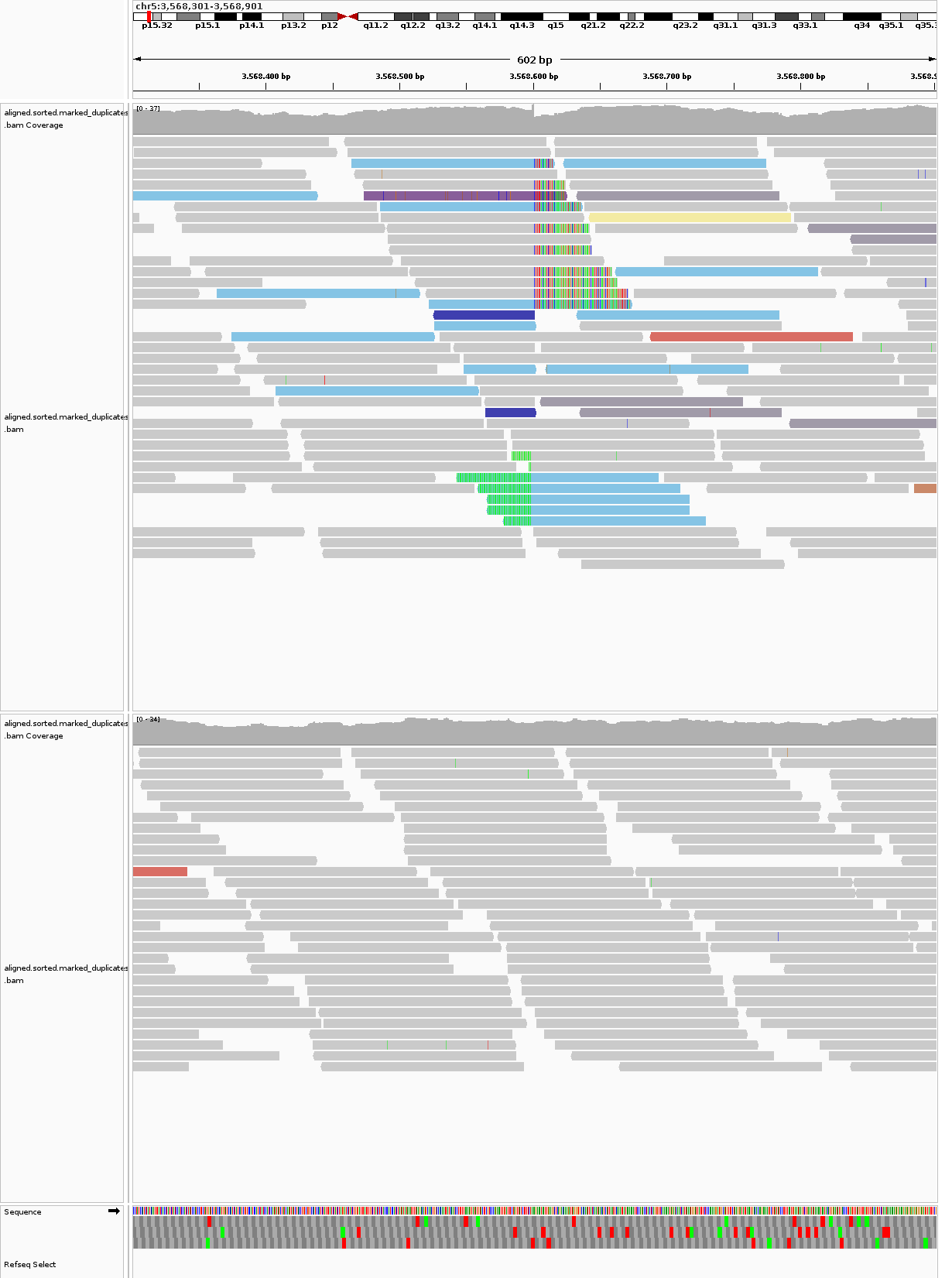

Supplement: Supplementary file 1 — Data S1. Compressed file containing the IGV screenshots for all the RetroTest exclusive insertions inspected in sample_21 and sample_28 WGS data, classified as true positives (TPs), false positives (FPs), and unconclusive. Both the tumor and normal BAM files were included in each screenshot. [file MOL2-19-3769-s003.zip › IGV_screenshots_illuminaWGS_TD2-RetroTest-exclusive_classified/PD0270a_retrotest_exclusive_IlluminaWGS/TPs/chr5_3568301-3568901.png]

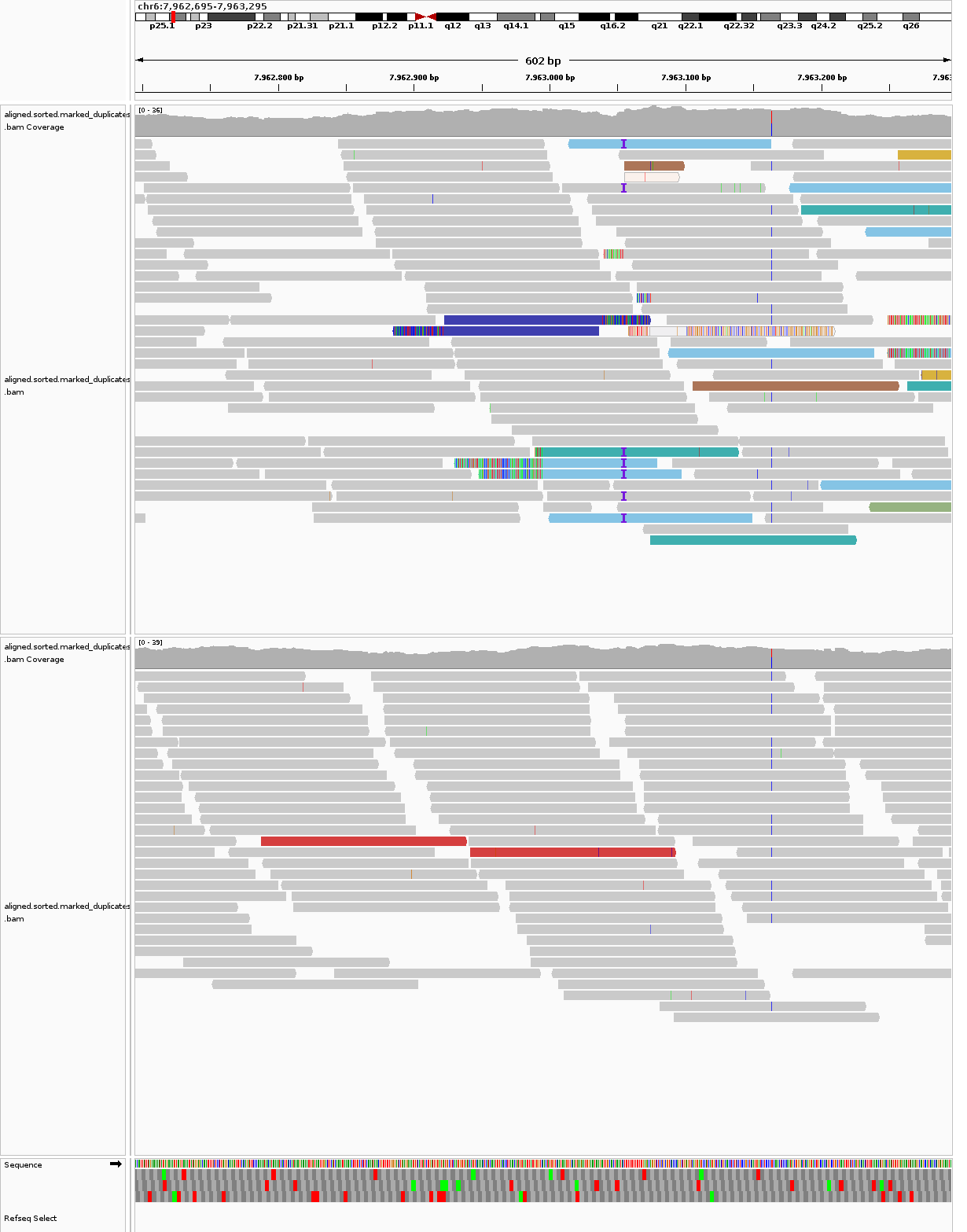

Supplement: Supplementary file 1 — Data S1. Compressed file containing the IGV screenshots for all the RetroTest exclusive insertions inspected in sample_21 and sample_28 WGS data, classified as true positives (TPs), false positives (FPs), and unconclusive. Both the tumor and normal BAM files were included in each screenshot. [file MOL2-19-3769-s003.zip › IGV_screenshots_illuminaWGS_TD2-RetroTest-exclusive_classified/PD0270a_retrotest_exclusive_IlluminaWGS/TPs/chr6_7962695-7963295.png]

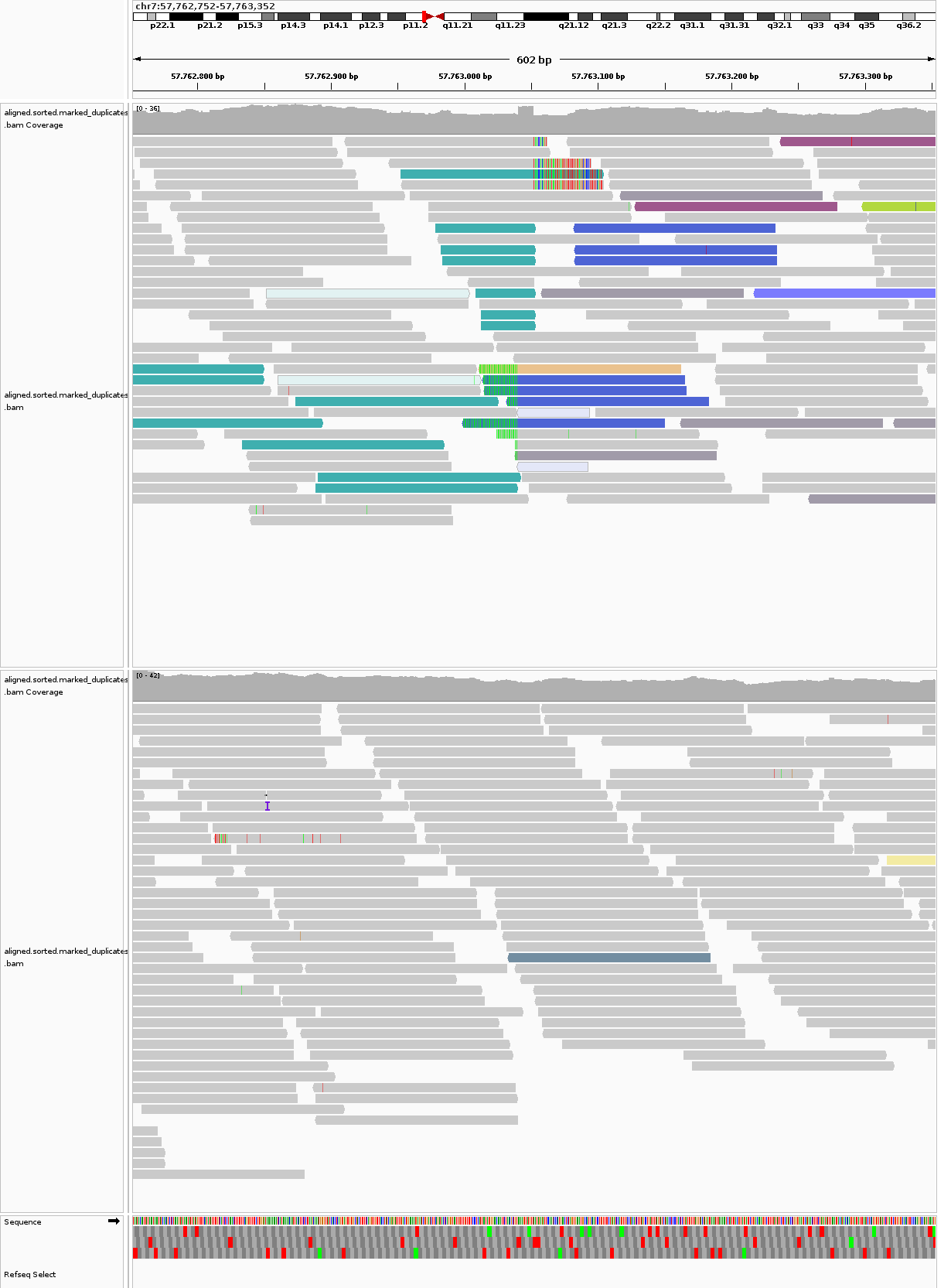

Supplement: Supplementary file 1 — Data S1. Compressed file containing the IGV screenshots for all the RetroTest exclusive insertions inspected in sample_21 and sample_28 WGS data, classified as true positives (TPs), false positives (FPs), and unconclusive. Both the tumor and normal BAM files were included in each screenshot. [file MOL2-19-3769-s003.zip › IGV_screenshots_illuminaWGS_TD2-RetroTest-exclusive_classified/PD0270a_retrotest_exclusive_IlluminaWGS/TPs/chr7_57762752-57763352.png]

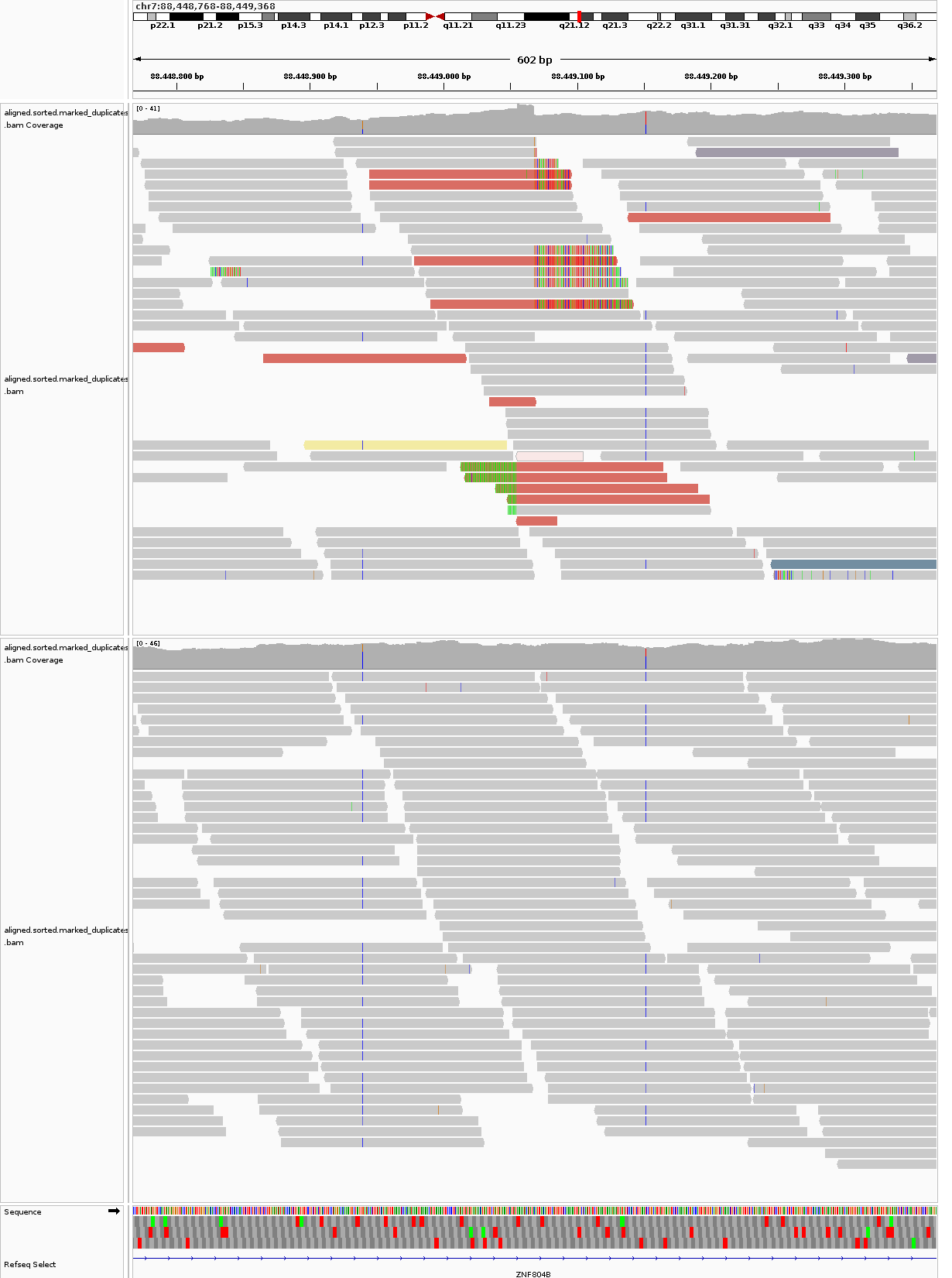

Supplement: Supplementary file 1 — Data S1. Compressed file containing the IGV screenshots for all the RetroTest exclusive insertions inspected in sample_21 and sample_28 WGS data, classified as true positives (TPs), false positives (FPs), and unconclusive. Both the tumor and normal BAM files were included in each screenshot. [file MOL2-19-3769-s003.zip › IGV_screenshots_illuminaWGS_TD2-RetroTest-exclusive_classified/PD0270a_retrotest_exclusive_IlluminaWGS/TPs/chr7_88448768-88449368.png]

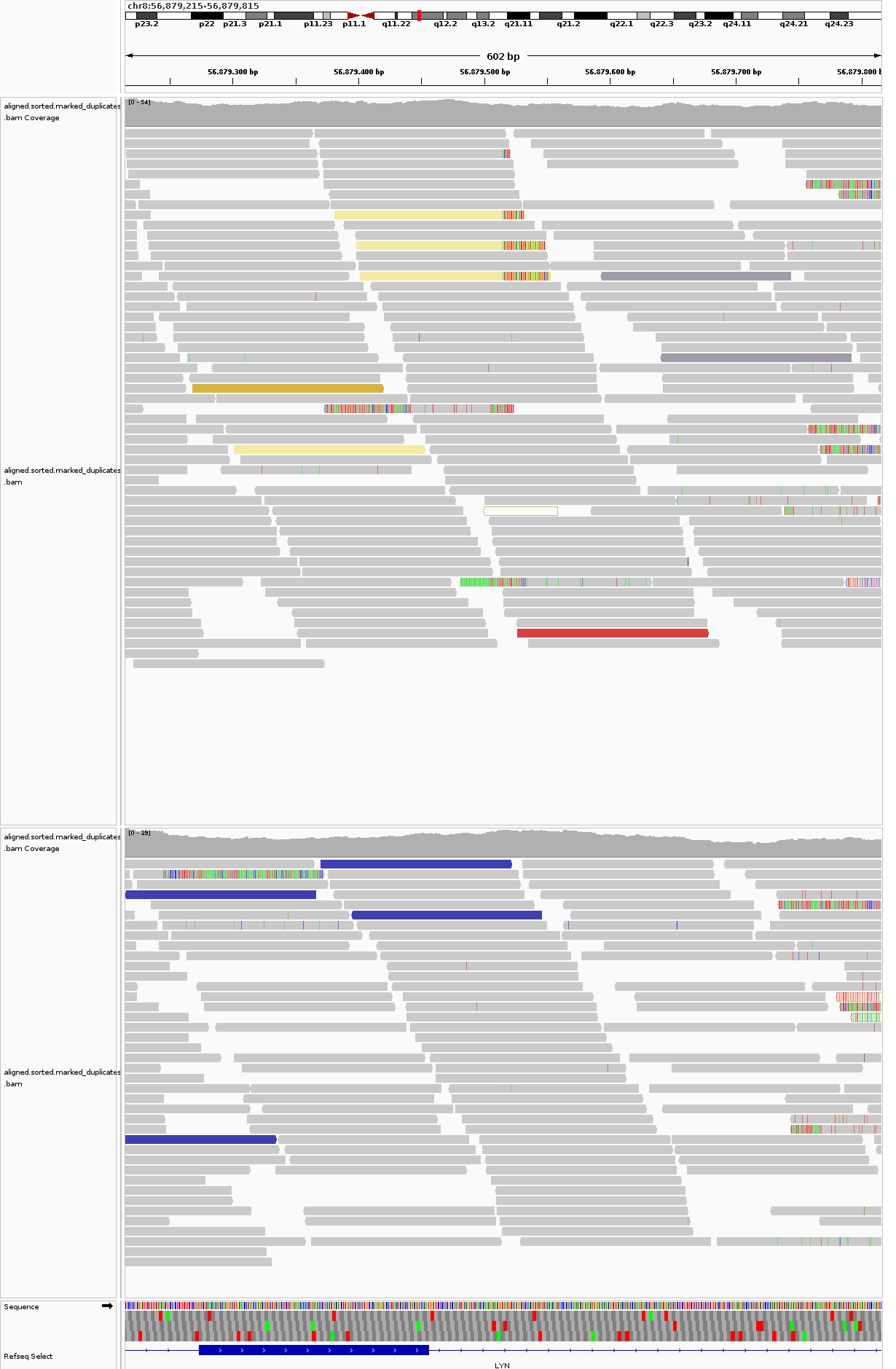

Supplement: Supplementary file 1 — Data S1. Compressed file containing the IGV screenshots for all the RetroTest exclusive insertions inspected in sample_21 and sample_28 WGS data, classified as true positives (TPs), false positives (FPs), and unconclusive. Both the tumor and normal BAM files were included in each screenshot. [file MOL2-19-3769-s003.zip › IGV_screenshots_illuminaWGS_TD2-RetroTest-exclusive_classified/PD0270a_retrotest_exclusive_IlluminaWGS/TPs/chr8_56879215-56879815.png]

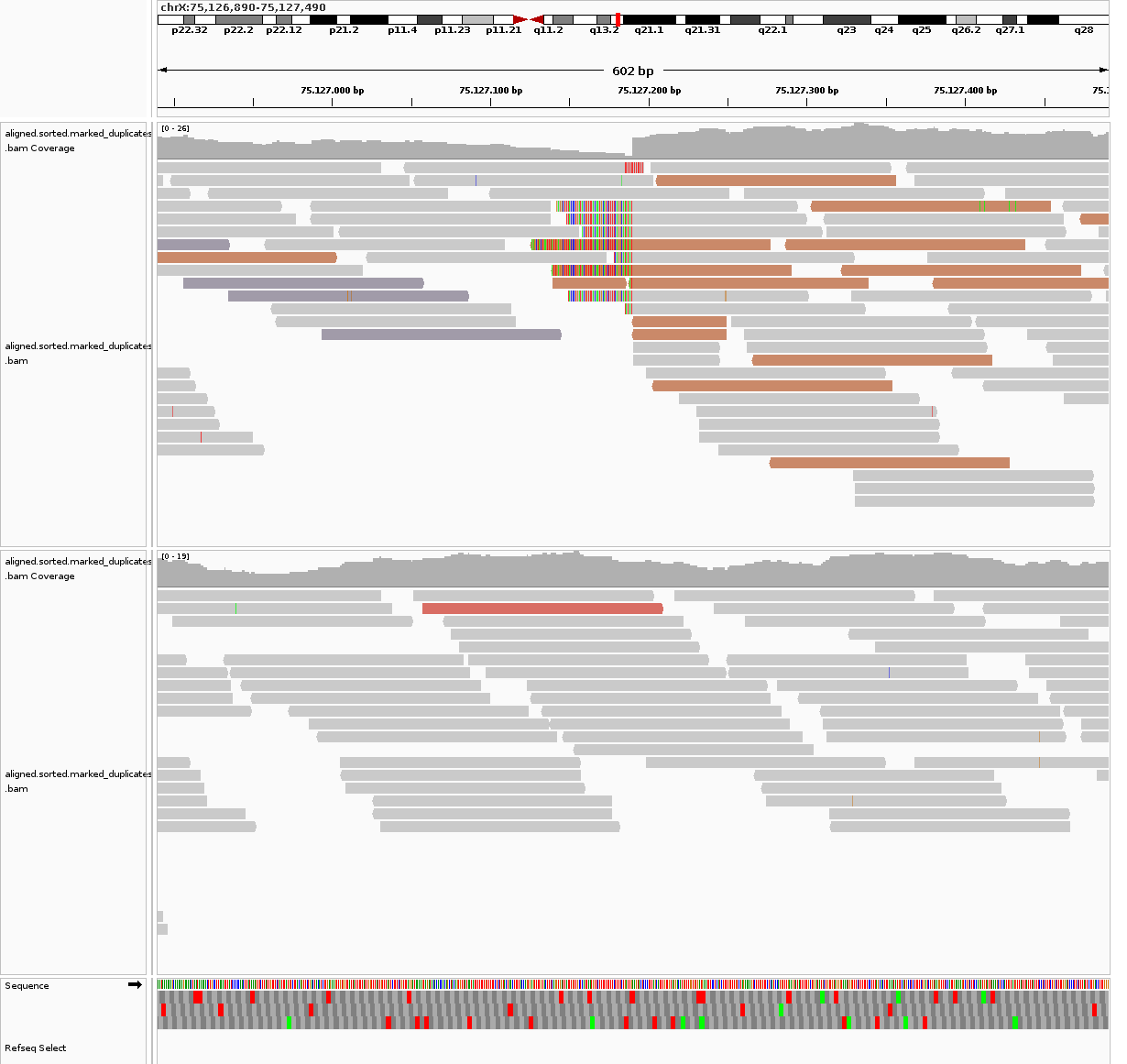

Supplement: Supplementary file 1 — Data S1. Compressed file containing the IGV screenshots for all the RetroTest exclusive insertions inspected in sample_21 and sample_28 WGS data, classified as true positives (TPs), false positives (FPs), and unconclusive. Both the tumor and normal BAM files were included in each screenshot. [file MOL2-19-3769-s003.zip › IGV_screenshots_illuminaWGS_TD2-RetroTest-exclusive_classified/PD0270a_retrotest_exclusive_IlluminaWGS/TPs/chrX_75126890-75127490.png]

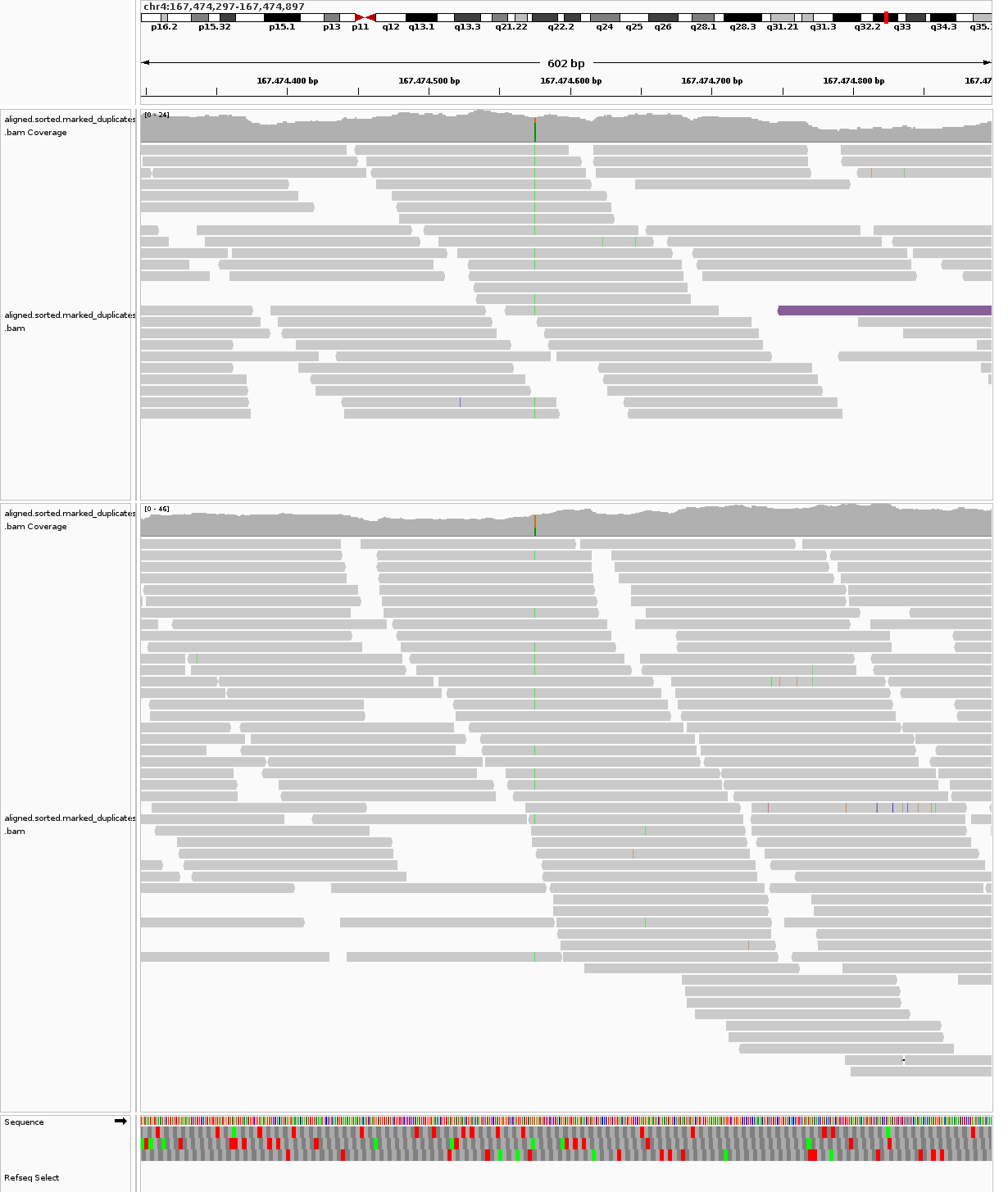

Supplement: Supplementary file 1 — Data S1. Compressed file containing the IGV screenshots for all the RetroTest exclusive insertions inspected in sample_21 and sample_28 WGS data, classified as true positives (TPs), false positives (FPs), and unconclusive. Both the tumor and normal BAM files were included in each screenshot. [file MOL2-19-3769-s003.zip › IGV_screenshots_illuminaWGS_TD2-RetroTest-exclusive_classified/PD0270a_retrotest_exclusive_IlluminaWGS/unconclusive/chr4_167474297-167474897.png]

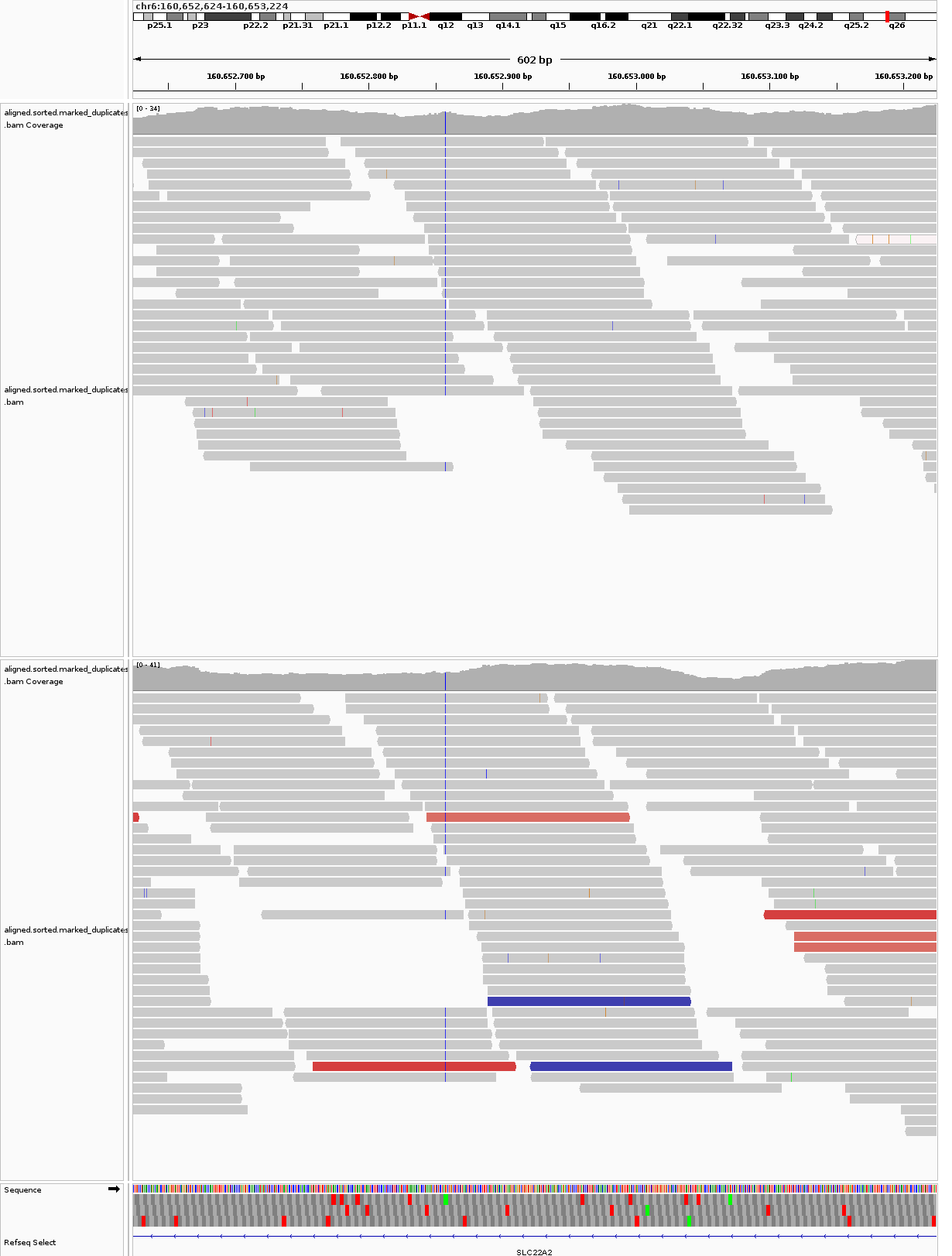

Supplement: Supplementary file 1 — Data S1. Compressed file containing the IGV screenshots for all the RetroTest exclusive insertions inspected in sample_21 and sample_28 WGS data, classified as true positives (TPs), false positives (FPs), and unconclusive. Both the tumor and normal BAM files were included in each screenshot. [file MOL2-19-3769-s003.zip › IGV_screenshots_illuminaWGS_TD2-RetroTest-exclusive_classified/PD0270a_retrotest_exclusive_IlluminaWGS/unconclusive/chr6_160652624-160653224.png]

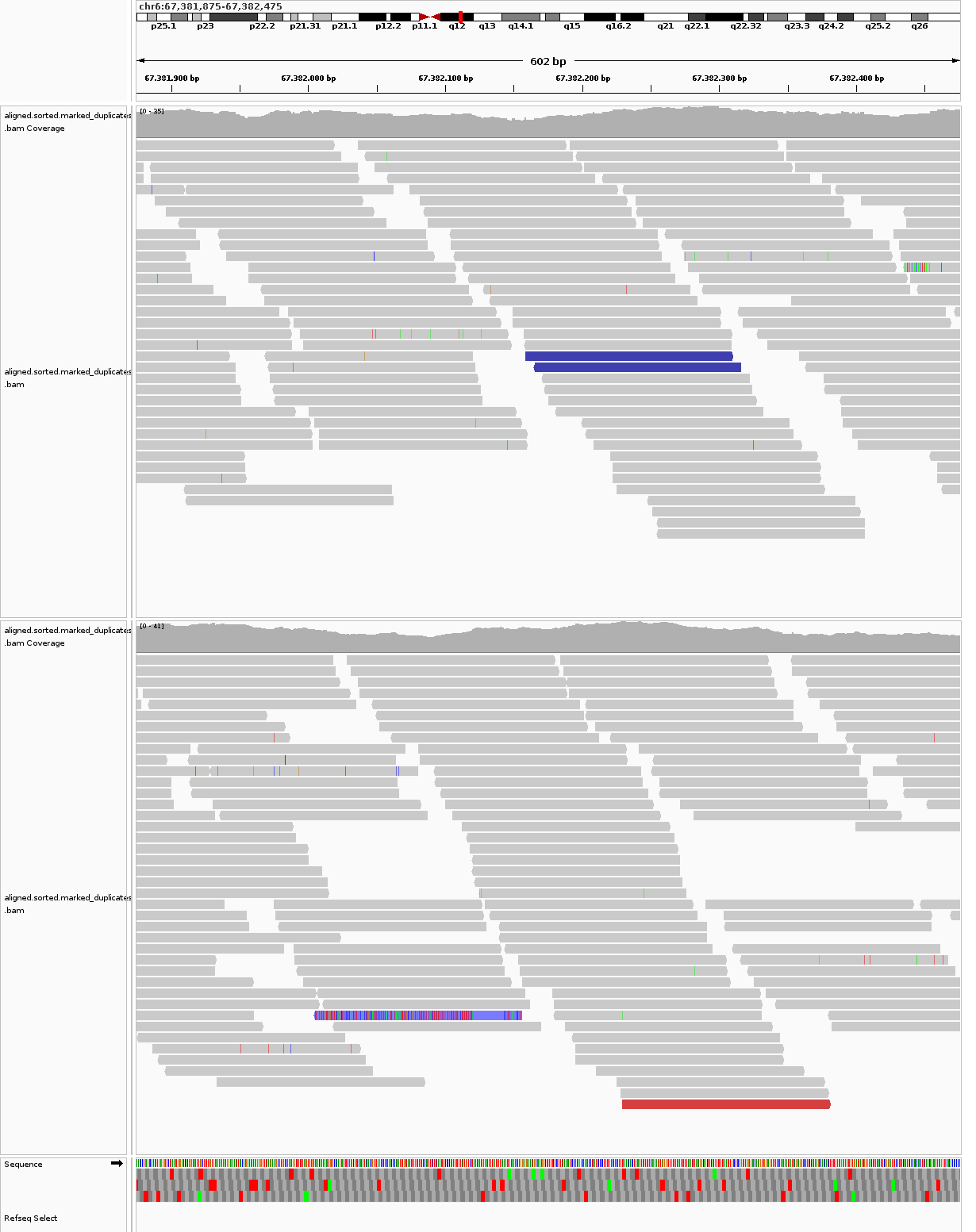

Supplement: Supplementary file 1 — Data S1. Compressed file containing the IGV screenshots for all the RetroTest exclusive insertions inspected in sample_21 and sample_28 WGS data, classified as true positives (TPs), false positives (FPs), and unconclusive. Both the tumor and normal BAM files were included in each screenshot. [file MOL2-19-3769-s003.zip › IGV_screenshots_illuminaWGS_TD2-RetroTest-exclusive_classified/PD0270a_retrotest_exclusive_IlluminaWGS/unconclusive/chr6_67381875-67382475.png]

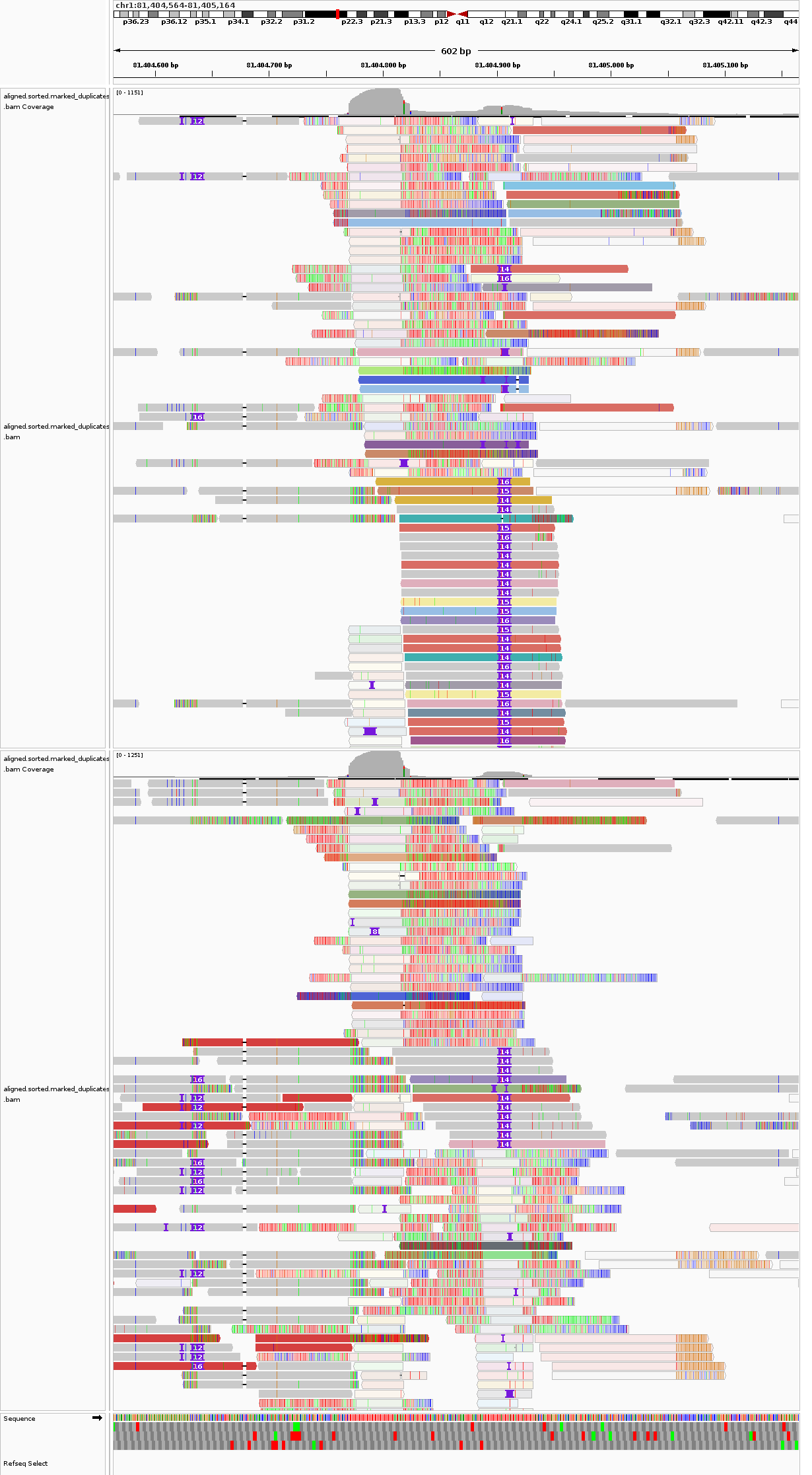

Supplement: Supplementary file 1 — Data S1. Compressed file containing the IGV screenshots for all the RetroTest exclusive insertions inspected in sample_21 and sample_28 WGS data, classified as true positives (TPs), false positives (FPs), and unconclusive. Both the tumor and normal BAM files were included in each screenshot. [file MOL2-19-3769-s003.zip › IGV_screenshots_illuminaWGS_TD2-RetroTest-exclusive_classified/PD0277a_retrotest_exclusive_IlluminaWGS/FPs/chr1_81404564-81405164.png]

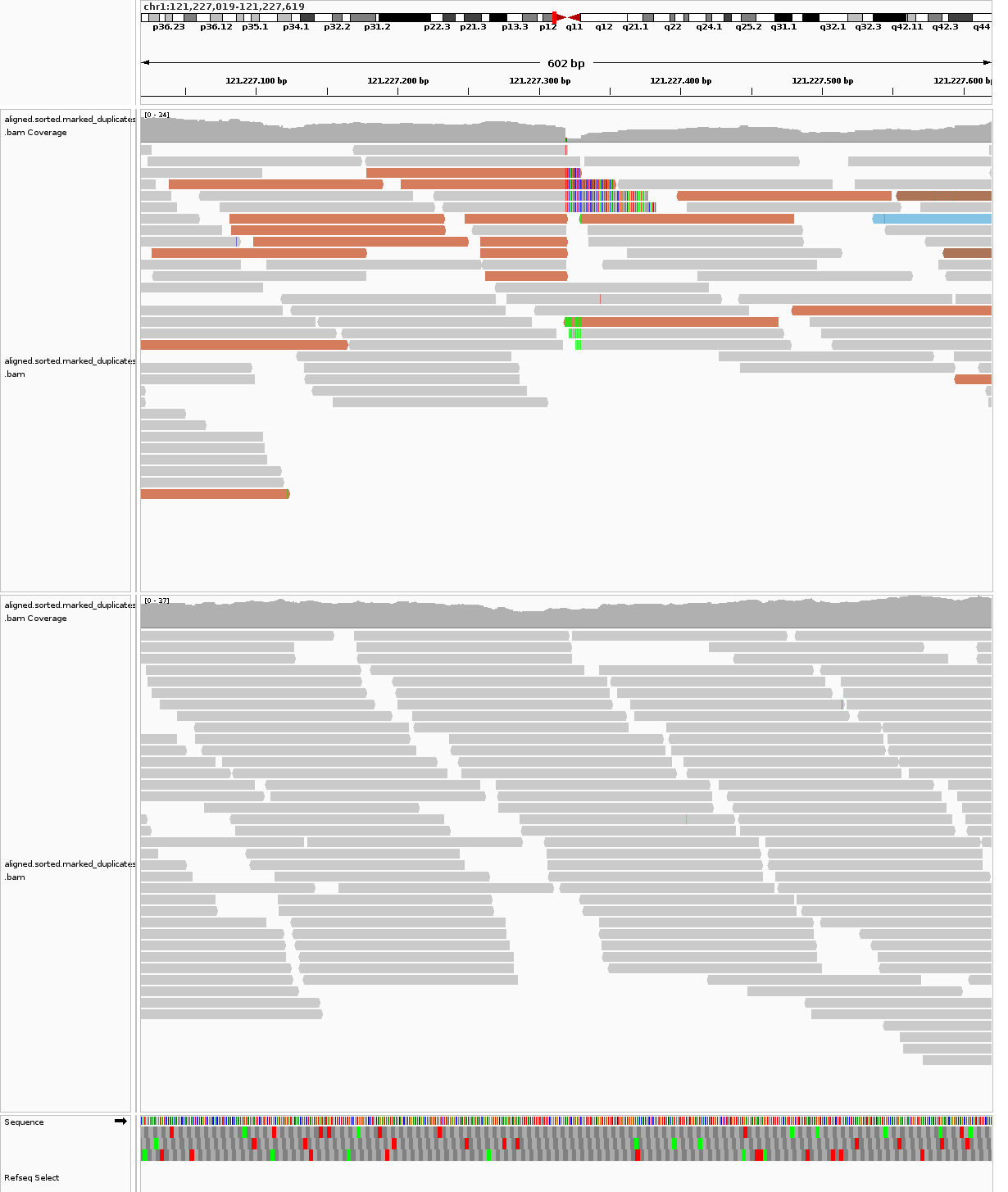

Supplement: Supplementary file 1 — Data S1. Compressed file containing the IGV screenshots for all the RetroTest exclusive insertions inspected in sample_21 and sample_28 WGS data, classified as true positives (TPs), false positives (FPs), and unconclusive. Both the tumor and normal BAM files were included in each screenshot. [file MOL2-19-3769-s003.zip › IGV_screenshots_illuminaWGS_TD2-RetroTest-exclusive_classified/PD0277a_retrotest_exclusive_IlluminaWGS/TPs/chr1_121227019-121227619.png]

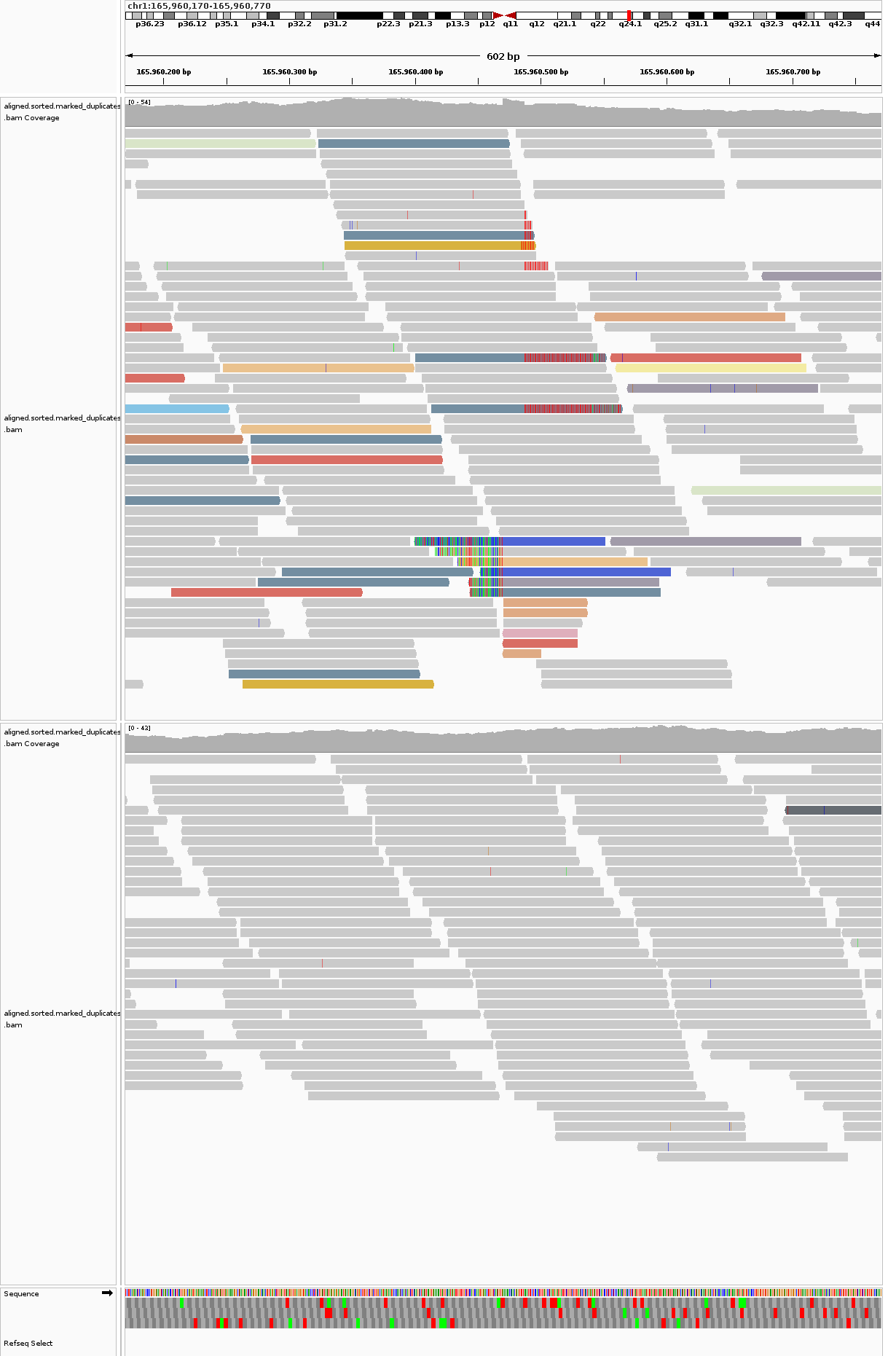

Supplement: Supplementary file 1 — Data S1. Compressed file containing the IGV screenshots for all the RetroTest exclusive insertions inspected in sample_21 and sample_28 WGS data, classified as true positives (TPs), false positives (FPs), and unconclusive. Both the tumor and normal BAM files were included in each screenshot. [file MOL2-19-3769-s003.zip › IGV_screenshots_illuminaWGS_TD2-RetroTest-exclusive_classified/PD0277a_retrotest_exclusive_IlluminaWGS/TPs/chr1_165960170-165960770.png]

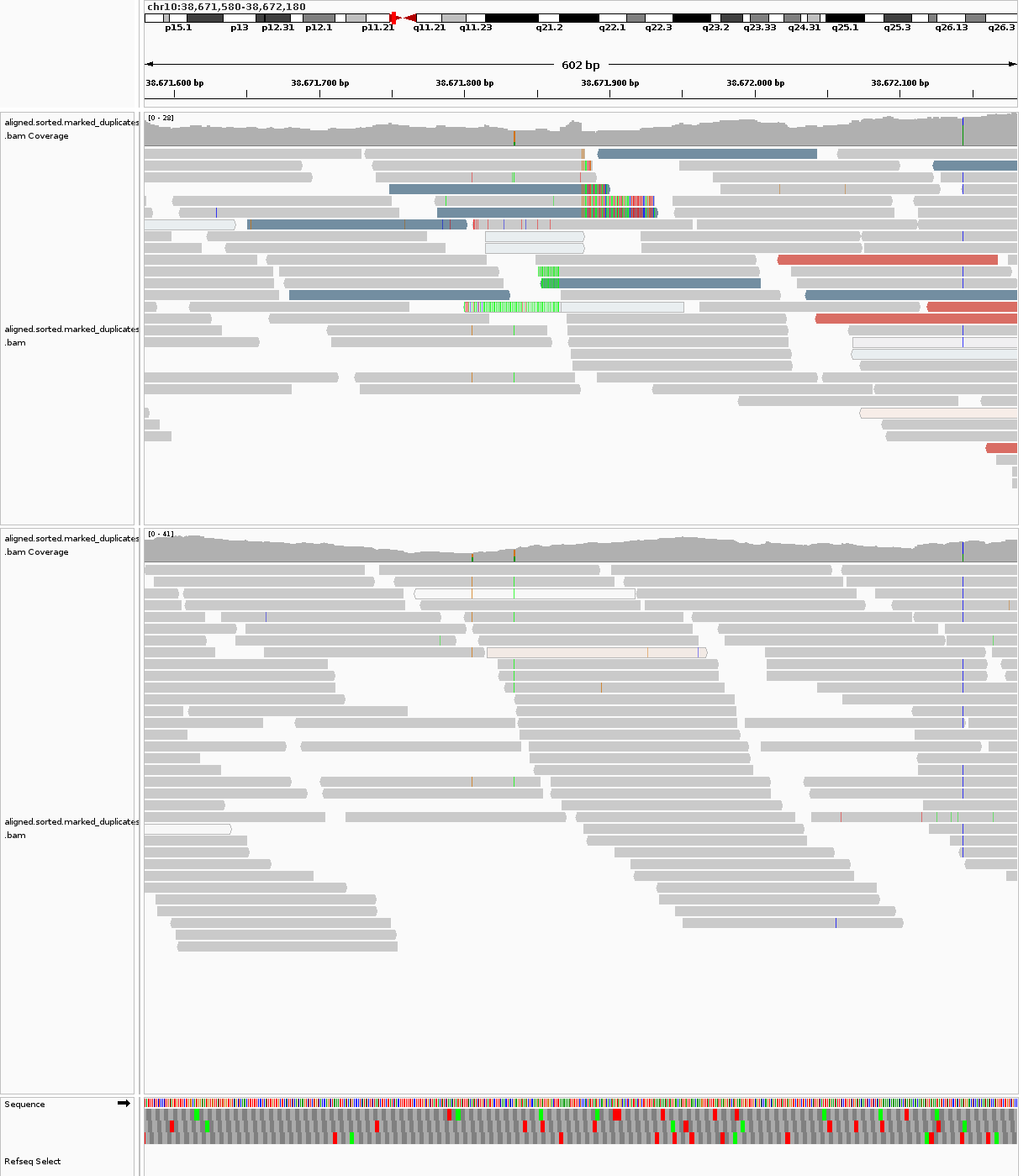

Supplement: Supplementary file 1 — Data S1. Compressed file containing the IGV screenshots for all the RetroTest exclusive insertions inspected in sample_21 and sample_28 WGS data, classified as true positives (TPs), false positives (FPs), and unconclusive. Both the tumor and normal BAM files were included in each screenshot. [file MOL2-19-3769-s003.zip › IGV_screenshots_illuminaWGS_TD2-RetroTest-exclusive_classified/PD0277a_retrotest_exclusive_IlluminaWGS/TPs/chr10_38671580-38672180.png]

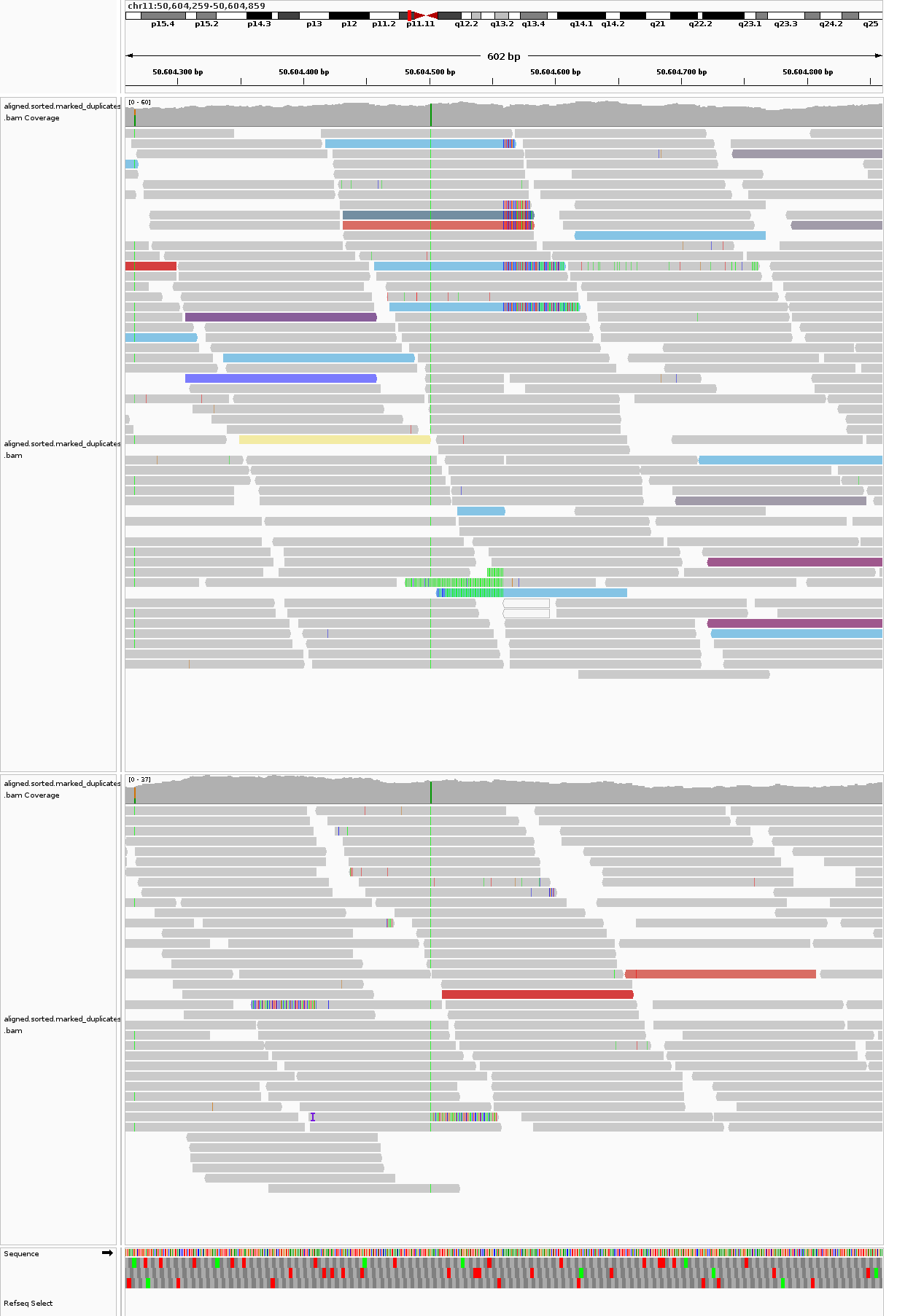

Supplement: Supplementary file 1 — Data S1. Compressed file containing the IGV screenshots for all the RetroTest exclusive insertions inspected in sample_21 and sample_28 WGS data, classified as true positives (TPs), false positives (FPs), and unconclusive. Both the tumor and normal BAM files were included in each screenshot. [file MOL2-19-3769-s003.zip › IGV_screenshots_illuminaWGS_TD2-RetroTest-exclusive_classified/PD0277a_retrotest_exclusive_IlluminaWGS/TPs/chr11_50604259-50604859.png]

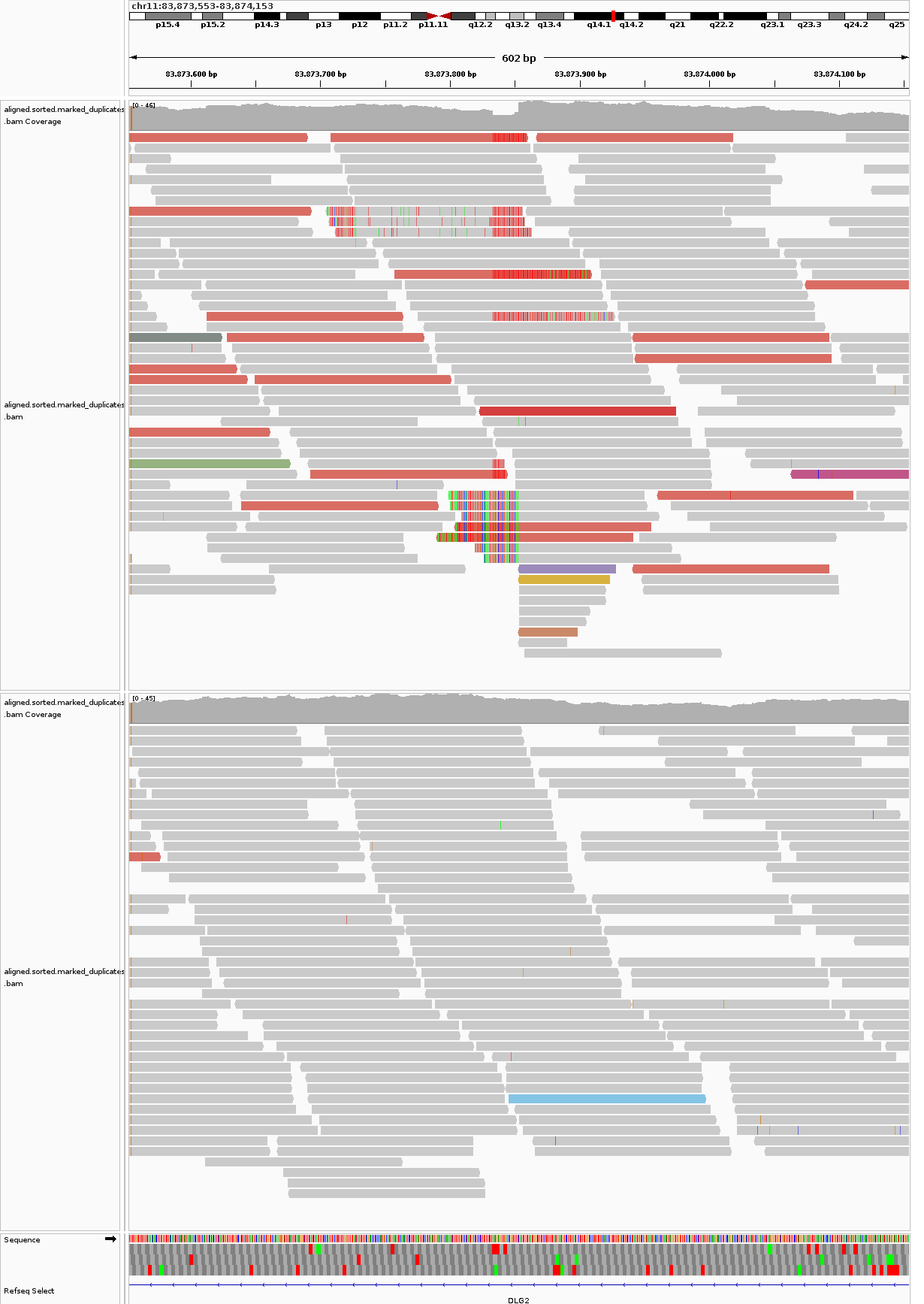

Supplement: Supplementary file 1 — Data S1. Compressed file containing the IGV screenshots for all the RetroTest exclusive insertions inspected in sample_21 and sample_28 WGS data, classified as true positives (TPs), false positives (FPs), and unconclusive. Both the tumor and normal BAM files were included in each screenshot. [file MOL2-19-3769-s003.zip › IGV_screenshots_illuminaWGS_TD2-RetroTest-exclusive_classified/PD0277a_retrotest_exclusive_IlluminaWGS/TPs/chr11_83873553-83874153.png]

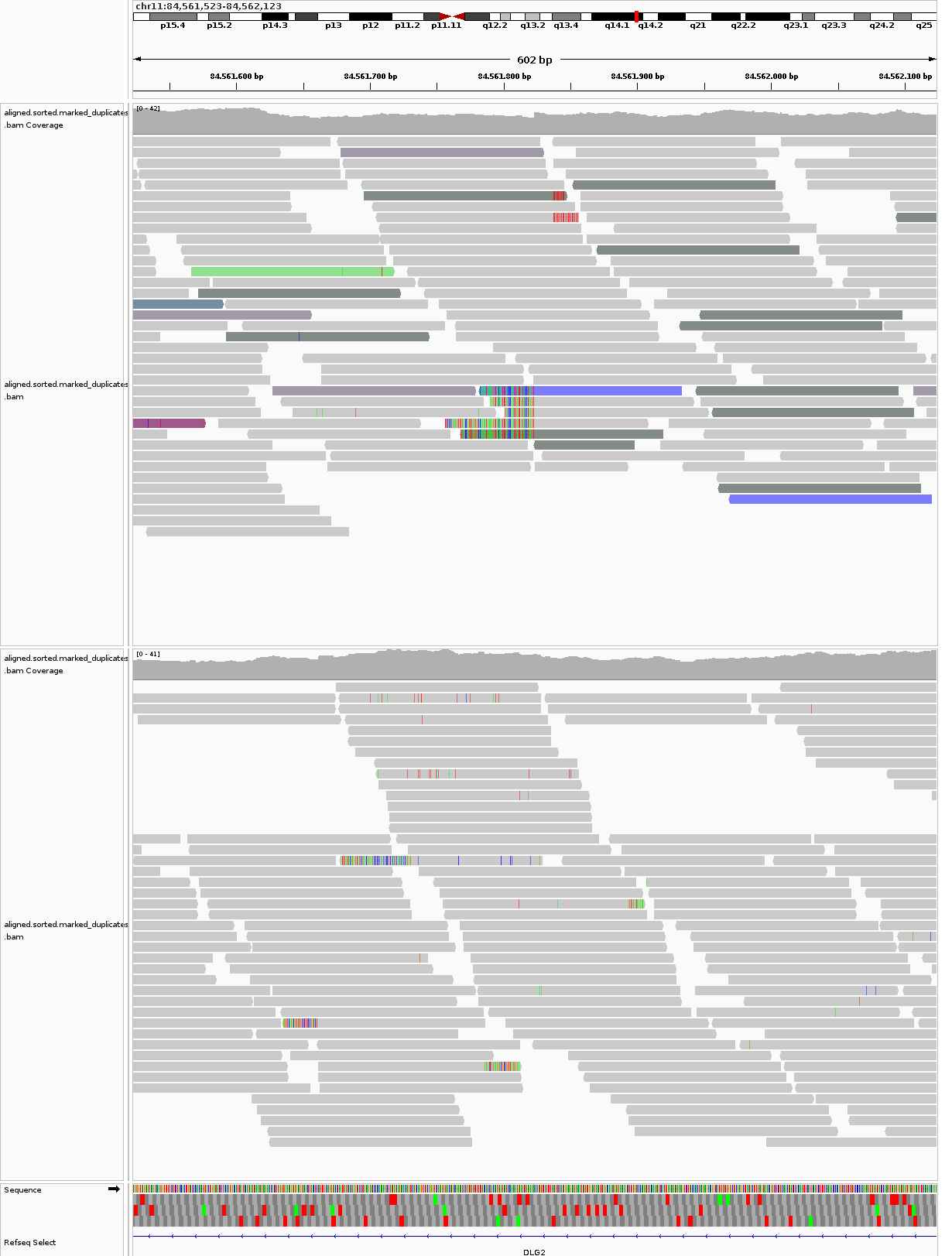

Supplement: Supplementary file 1 — Data S1. Compressed file containing the IGV screenshots for all the RetroTest exclusive insertions inspected in sample_21 and sample_28 WGS data, classified as true positives (TPs), false positives (FPs), and unconclusive. Both the tumor and normal BAM files were included in each screenshot. [file MOL2-19-3769-s003.zip › IGV_screenshots_illuminaWGS_TD2-RetroTest-exclusive_classified/PD0277a_retrotest_exclusive_IlluminaWGS/TPs/chr11_84561523-84562123.png]

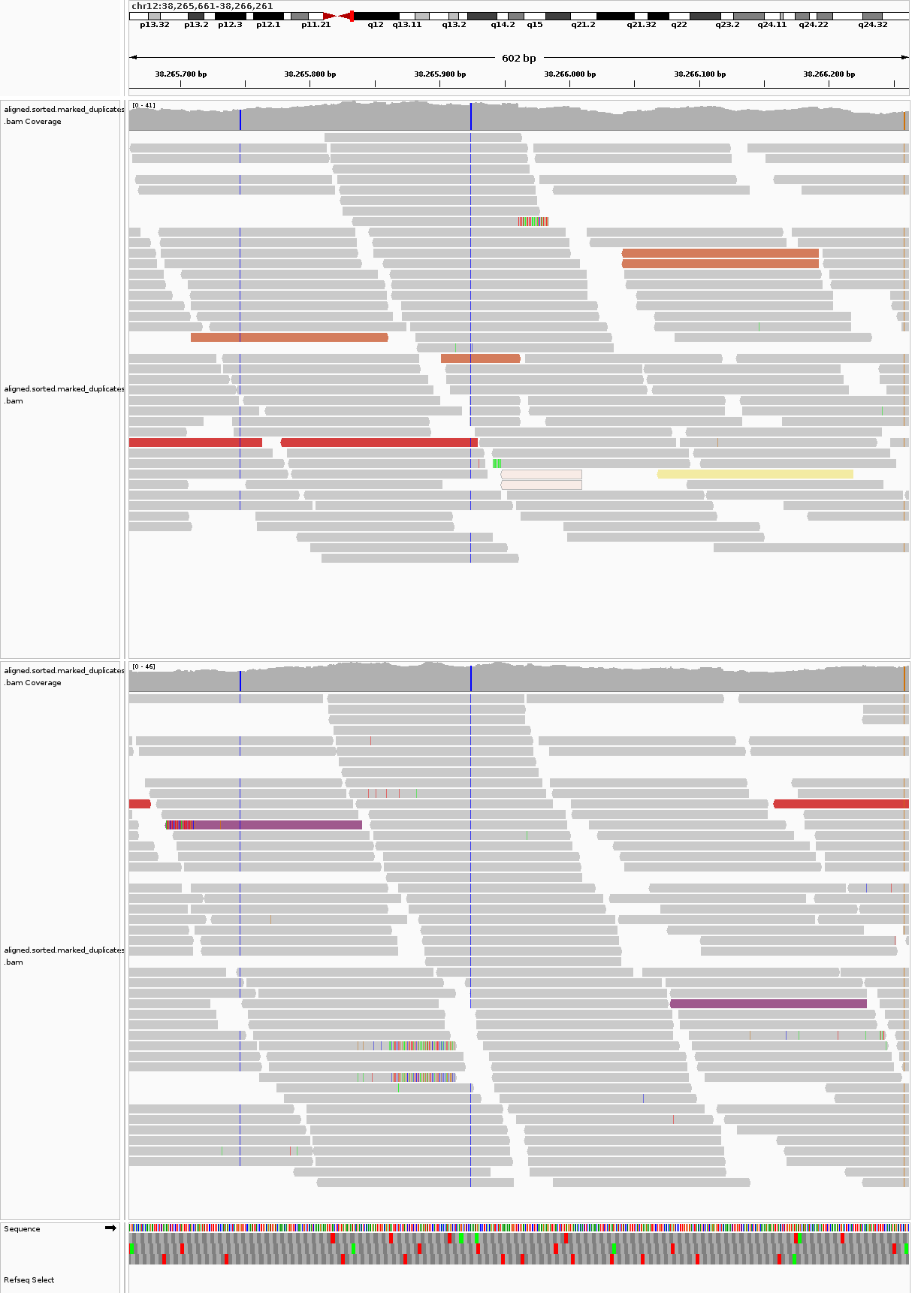

Supplement: Supplementary file 1 — Data S1. Compressed file containing the IGV screenshots for all the RetroTest exclusive insertions inspected in sample_21 and sample_28 WGS data, classified as true positives (TPs), false positives (FPs), and unconclusive. Both the tumor and normal BAM files were included in each screenshot. [file MOL2-19-3769-s003.zip › IGV_screenshots_illuminaWGS_TD2-RetroTest-exclusive_classified/PD0277a_retrotest_exclusive_IlluminaWGS/TPs/chr12_38265661-38266261.png]

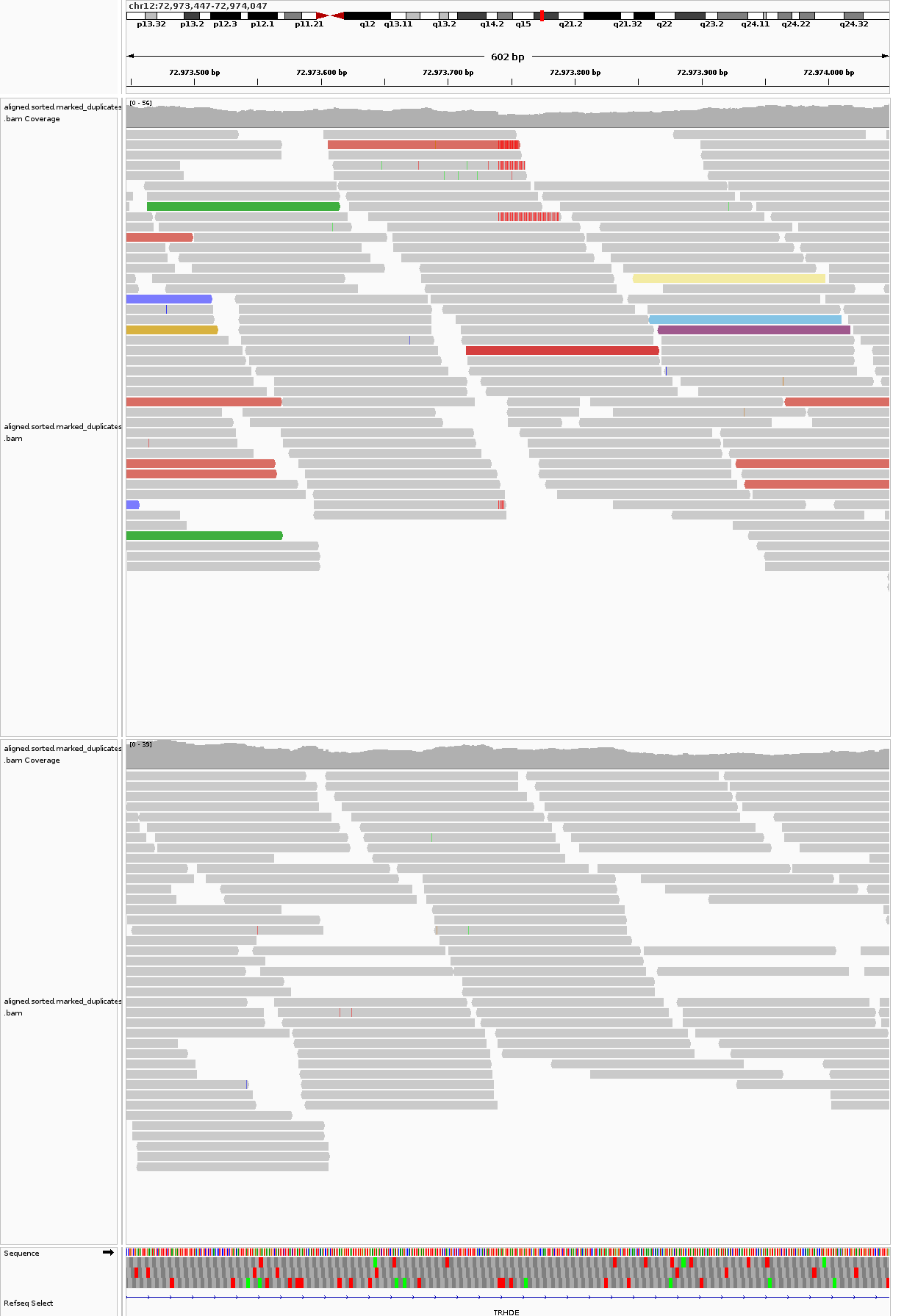

Supplement: Supplementary file 1 — Data S1. Compressed file containing the IGV screenshots for all the RetroTest exclusive insertions inspected in sample_21 and sample_28 WGS data, classified as true positives (TPs), false positives (FPs), and unconclusive. Both the tumor and normal BAM files were included in each screenshot. [file MOL2-19-3769-s003.zip › IGV_screenshots_illuminaWGS_TD2-RetroTest-exclusive_classified/PD0277a_retrotest_exclusive_IlluminaWGS/TPs/chr12_72973447-72974047.png]

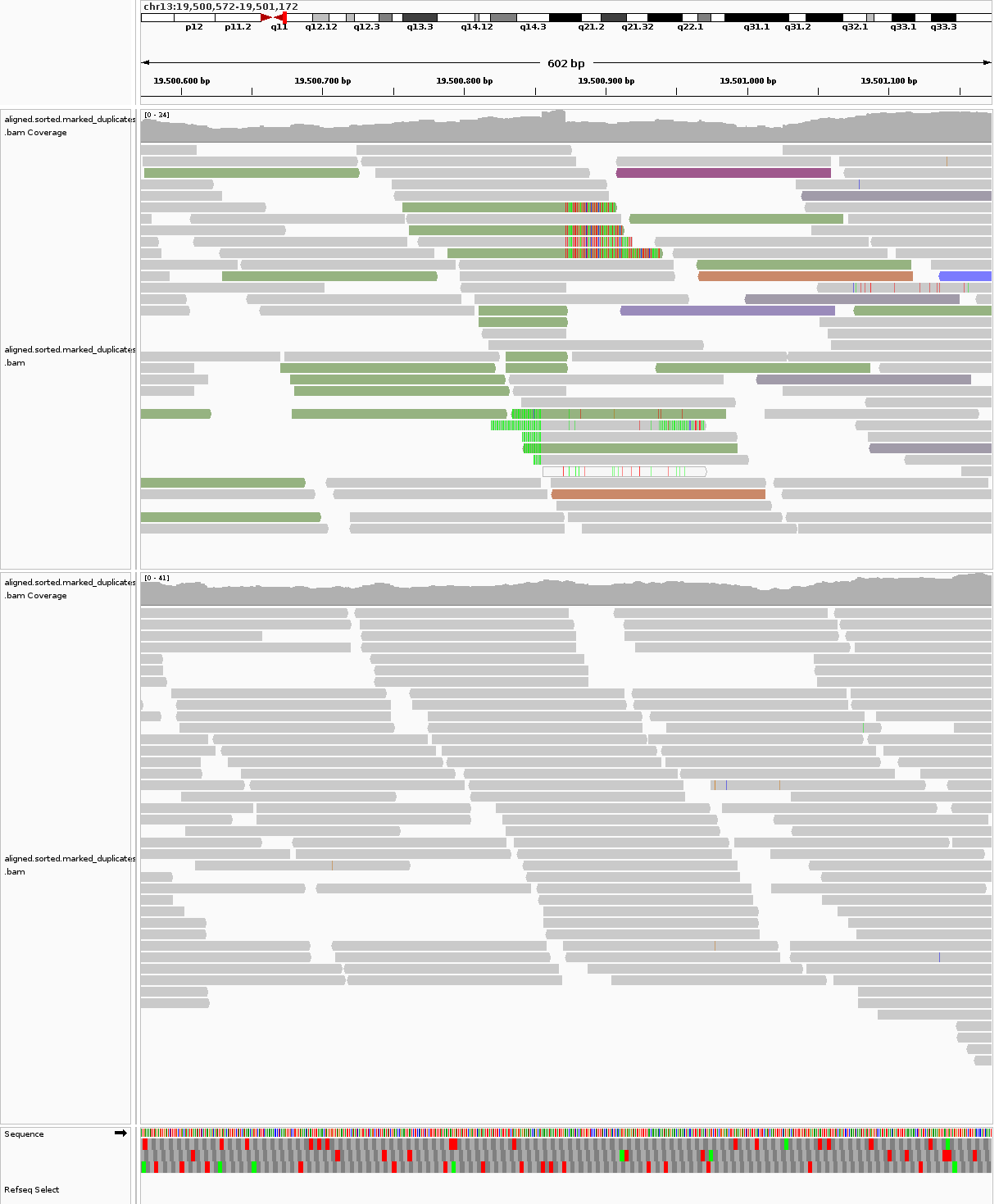

Supplement: Supplementary file 1 — Data S1. Compressed file containing the IGV screenshots for all the RetroTest exclusive insertions inspected in sample_21 and sample_28 WGS data, classified as true positives (TPs), false positives (FPs), and unconclusive. Both the tumor and normal BAM files were included in each screenshot. [file MOL2-19-3769-s003.zip › IGV_screenshots_illuminaWGS_TD2-RetroTest-exclusive_classified/PD0277a_retrotest_exclusive_IlluminaWGS/TPs/chr13_19500572-19501172.png]

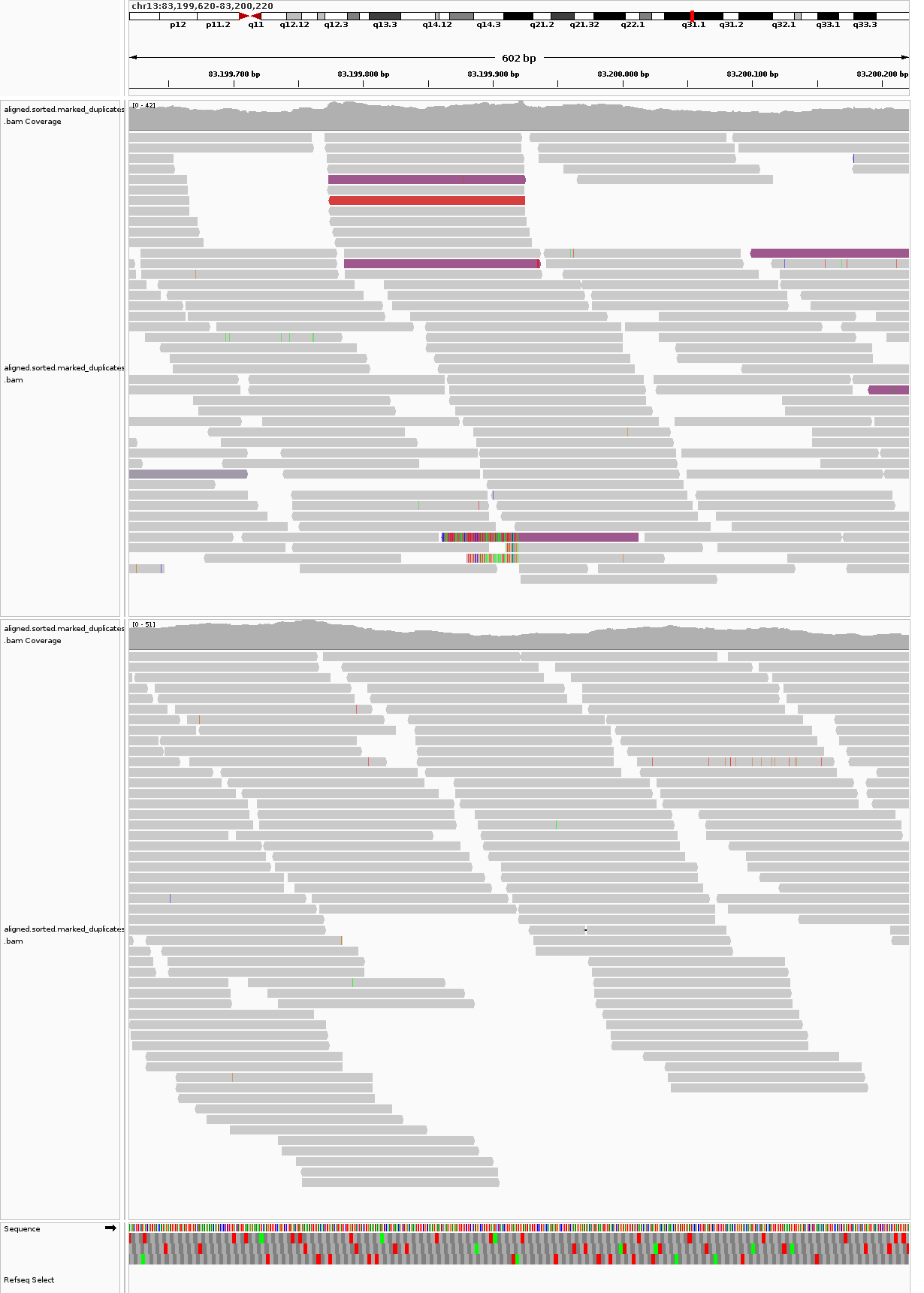

Supplement: Supplementary file 1 — Data S1. Compressed file containing the IGV screenshots for all the RetroTest exclusive insertions inspected in sample_21 and sample_28 WGS data, classified as true positives (TPs), false positives (FPs), and unconclusive. Both the tumor and normal BAM files were included in each screenshot. [file MOL2-19-3769-s003.zip › IGV_screenshots_illuminaWGS_TD2-RetroTest-exclusive_classified/PD0277a_retrotest_exclusive_IlluminaWGS/TPs/chr13_83199620-83200220.png]

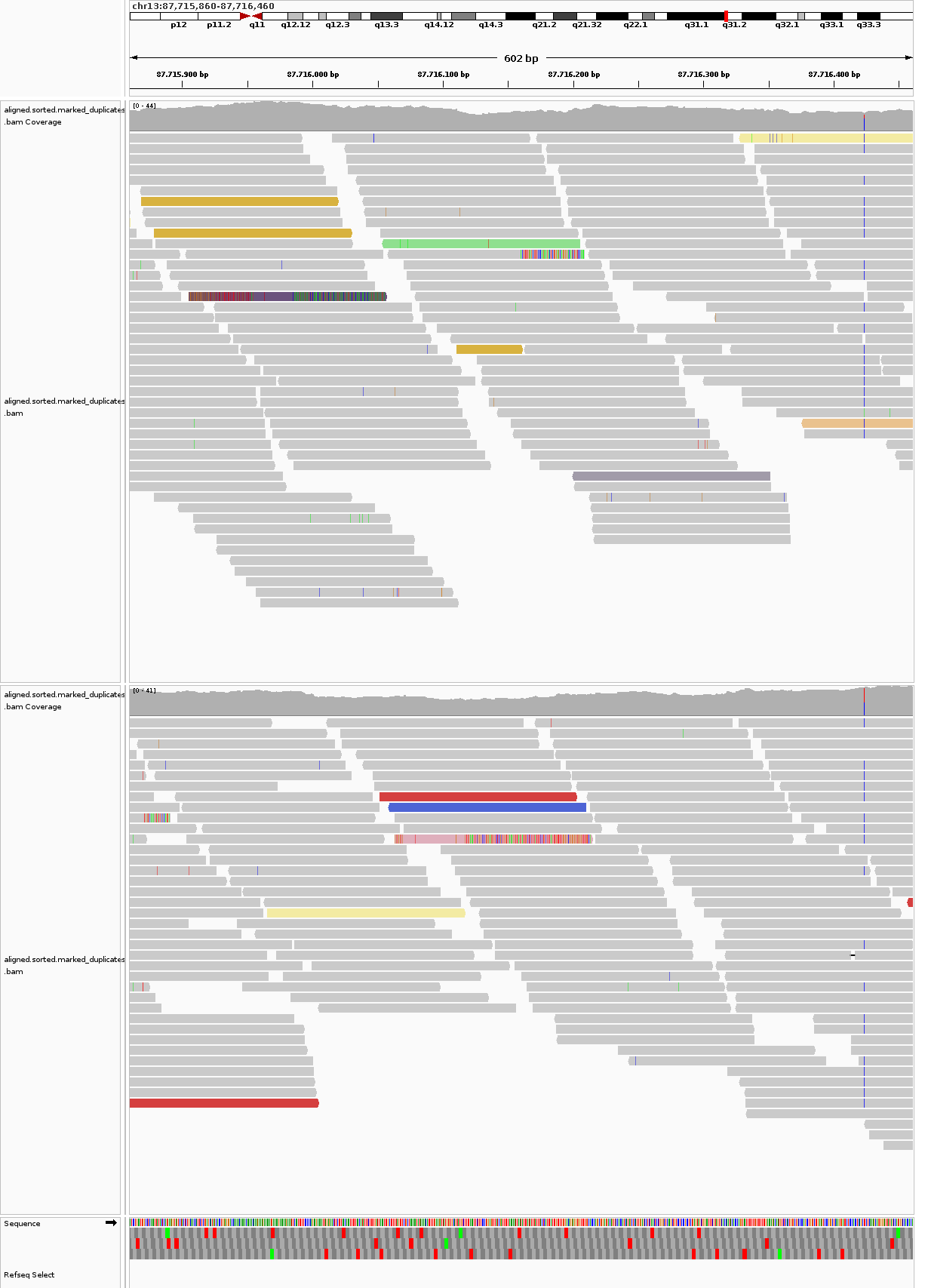

Supplement: Supplementary file 1 — Data S1. Compressed file containing the IGV screenshots for all the RetroTest exclusive insertions inspected in sample_21 and sample_28 WGS data, classified as true positives (TPs), false positives (FPs), and unconclusive. Both the tumor and normal BAM files were included in each screenshot. [file MOL2-19-3769-s003.zip › IGV_screenshots_illuminaWGS_TD2-RetroTest-exclusive_classified/PD0277a_retrotest_exclusive_IlluminaWGS/TPs/chr13_87715860-87716460.png]

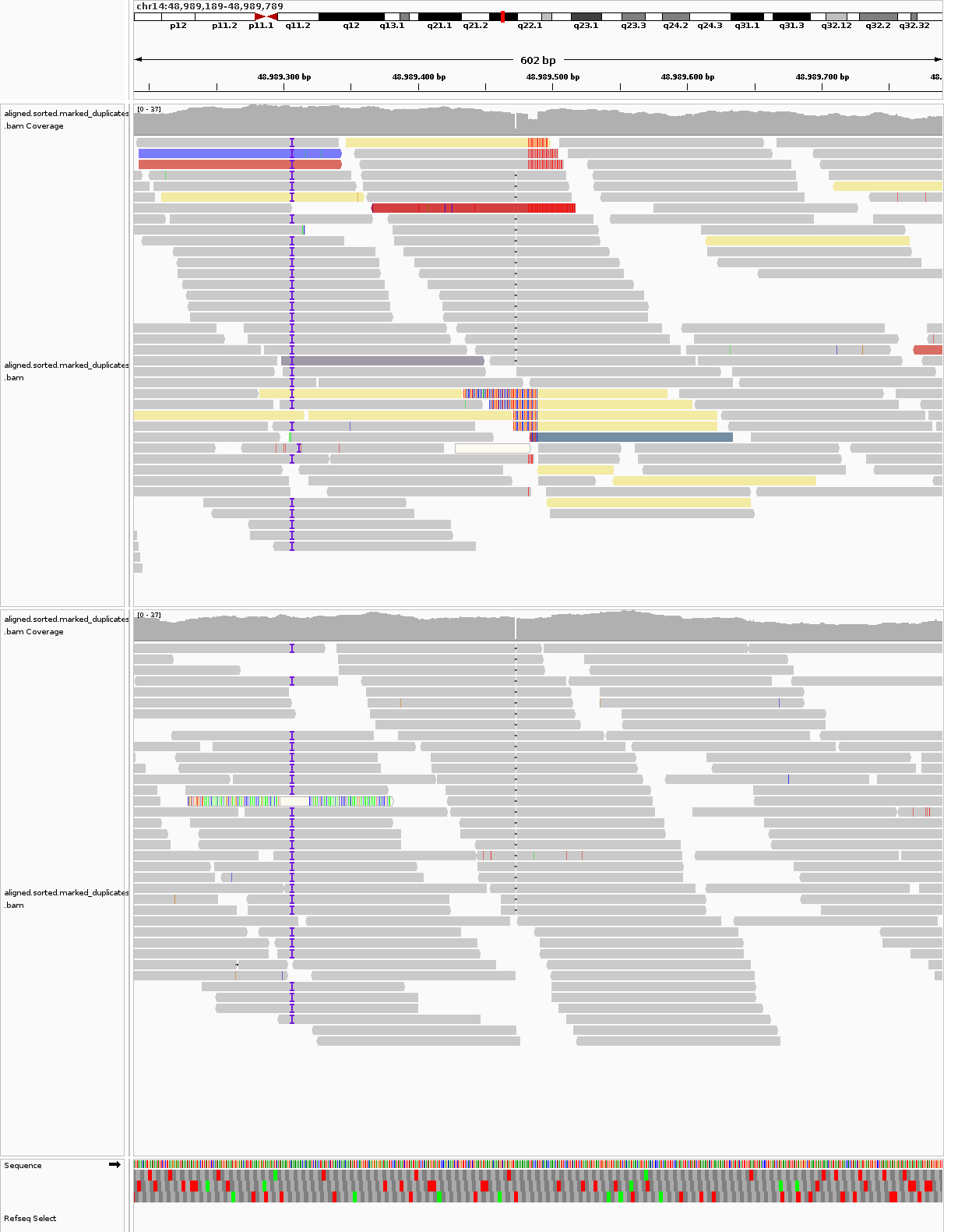

Supplement: Supplementary file 1 — Data S1. Compressed file containing the IGV screenshots for all the RetroTest exclusive insertions inspected in sample_21 and sample_28 WGS data, classified as true positives (TPs), false positives (FPs), and unconclusive. Both the tumor and normal BAM files were included in each screenshot. [file MOL2-19-3769-s003.zip › IGV_screenshots_illuminaWGS_TD2-RetroTest-exclusive_classified/PD0277a_retrotest_exclusive_IlluminaWGS/TPs/chr14_48989189-48989789.png]

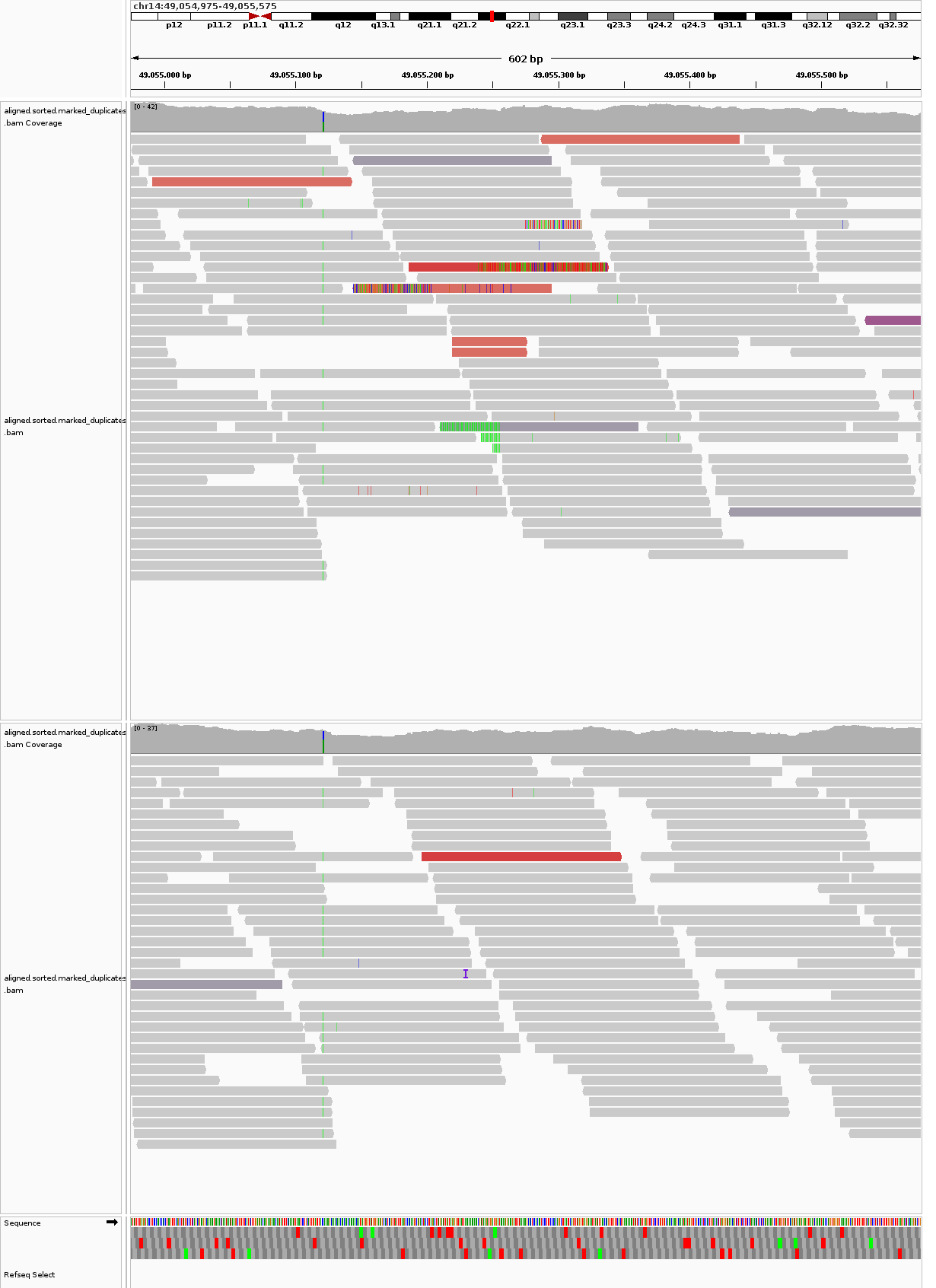

Supplement: Supplementary file 1 — Data S1. Compressed file containing the IGV screenshots for all the RetroTest exclusive insertions inspected in sample_21 and sample_28 WGS data, classified as true positives (TPs), false positives (FPs), and unconclusive. Both the tumor and normal BAM files were included in each screenshot. [file MOL2-19-3769-s003.zip › IGV_screenshots_illuminaWGS_TD2-RetroTest-exclusive_classified/PD0277a_retrotest_exclusive_IlluminaWGS/TPs/chr14_49054975-49055575.png]

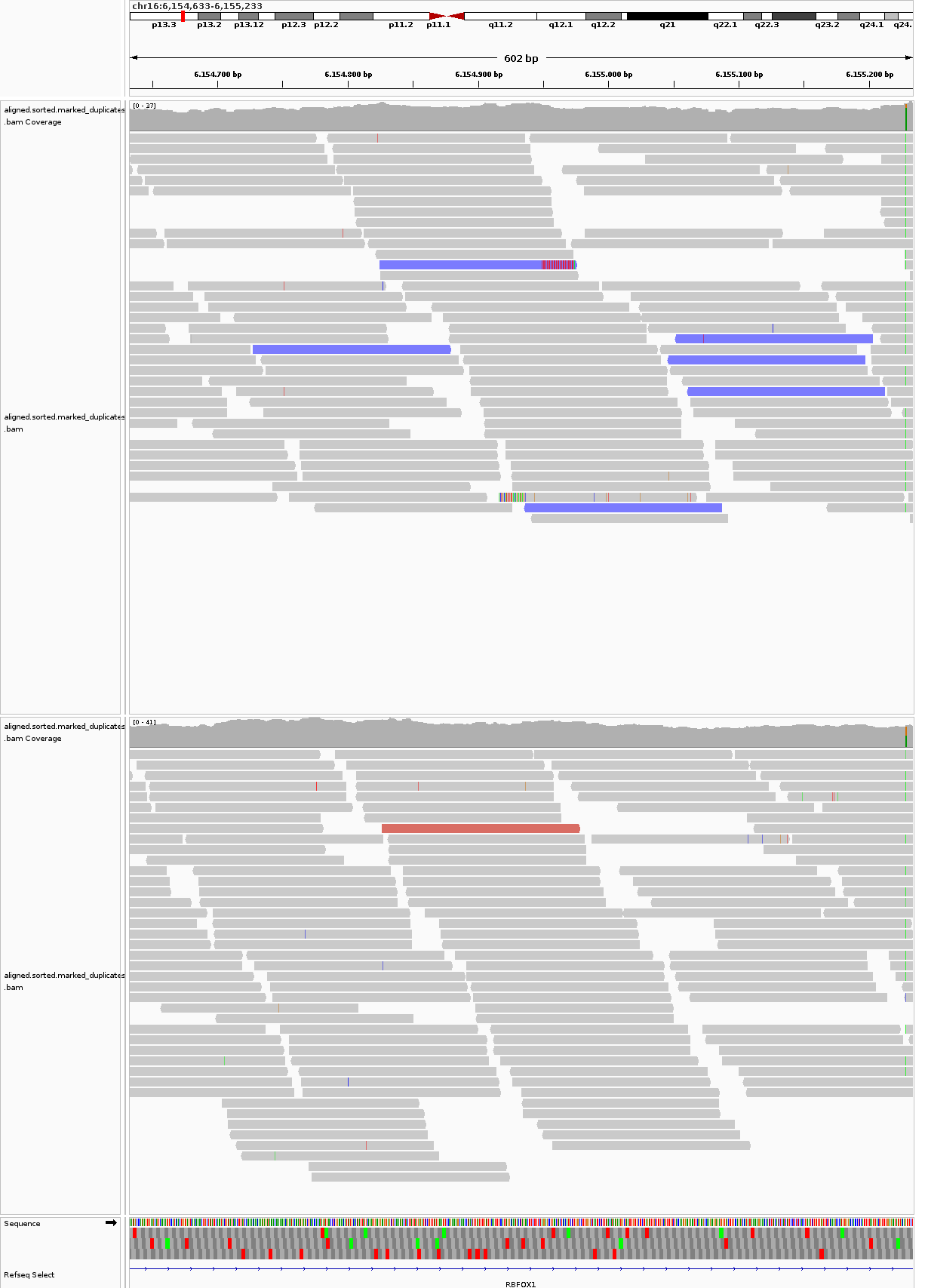

Supplement: Supplementary file 1 — Data S1. Compressed file containing the IGV screenshots for all the RetroTest exclusive insertions inspected in sample_21 and sample_28 WGS data, classified as true positives (TPs), false positives (FPs), and unconclusive. Both the tumor and normal BAM files were included in each screenshot. [file MOL2-19-3769-s003.zip › IGV_screenshots_illuminaWGS_TD2-RetroTest-exclusive_classified/PD0277a_retrotest_exclusive_IlluminaWGS/TPs/chr16_6154633-6155233.png]

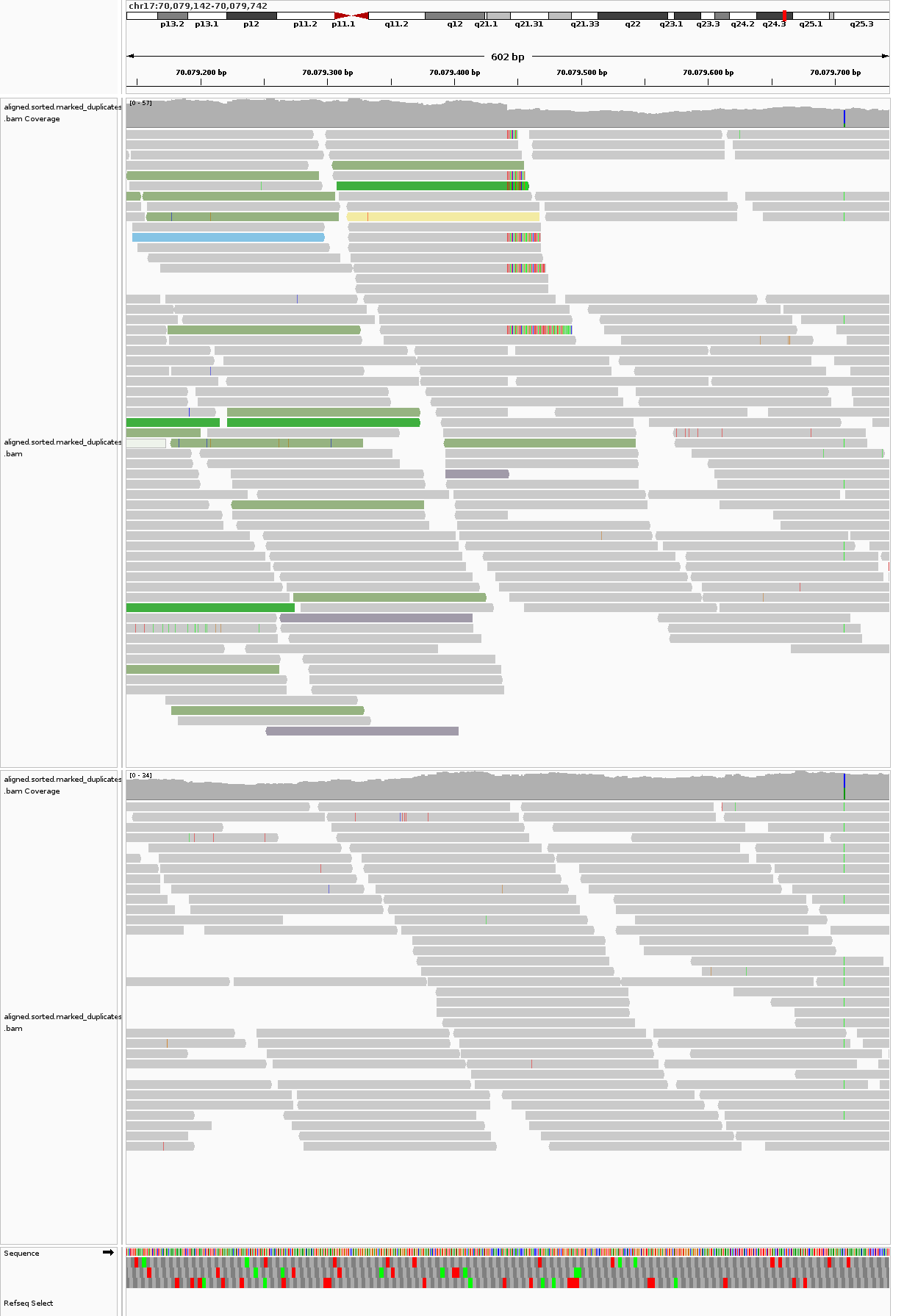

Supplement: Supplementary file 1 — Data S1. Compressed file containing the IGV screenshots for all the RetroTest exclusive insertions inspected in sample_21 and sample_28 WGS data, classified as true positives (TPs), false positives (FPs), and unconclusive. Both the tumor and normal BAM files were included in each screenshot. [file MOL2-19-3769-s003.zip › IGV_screenshots_illuminaWGS_TD2-RetroTest-exclusive_classified/PD0277a_retrotest_exclusive_IlluminaWGS/TPs/chr17_70079142-70079742.png]

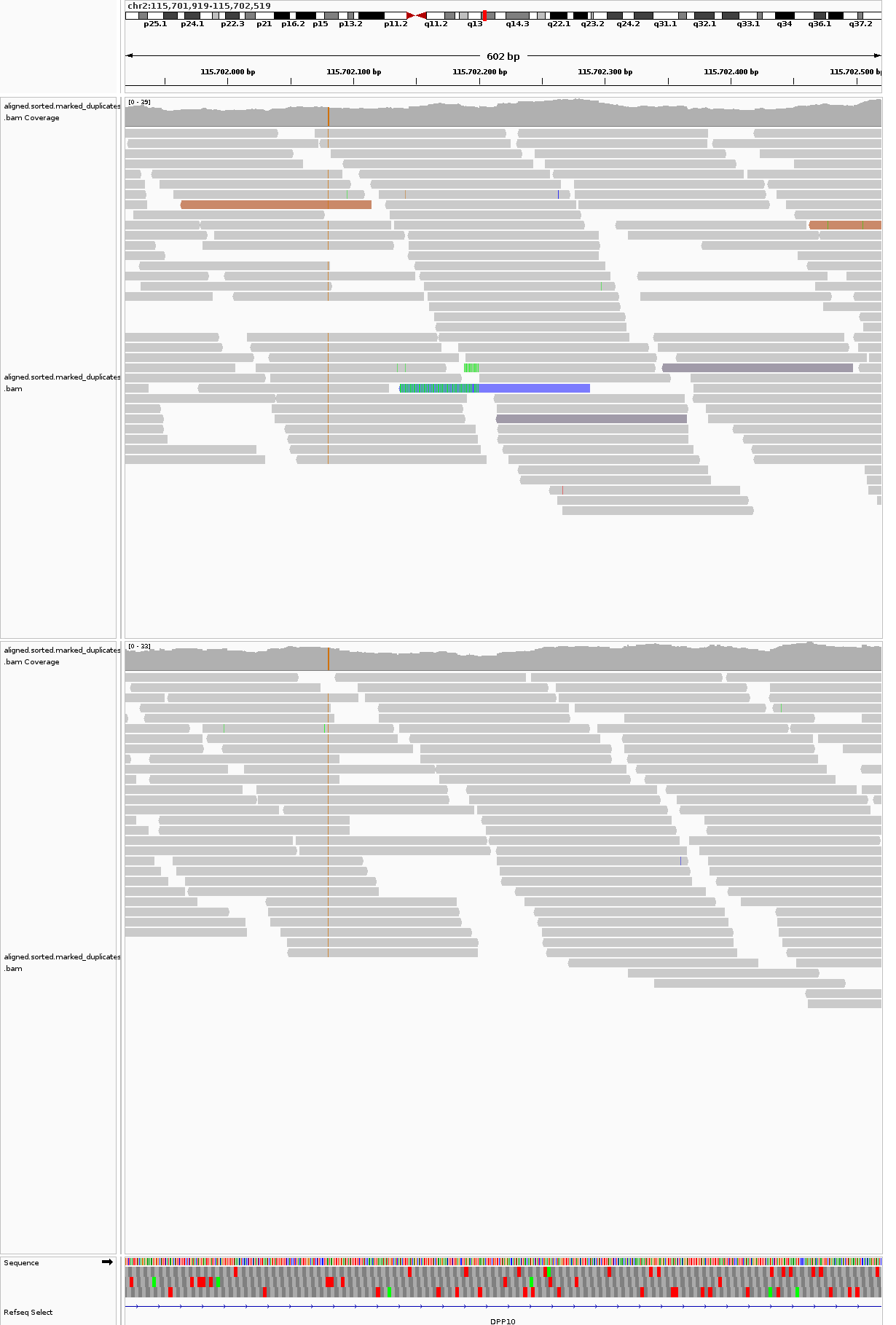

Supplement: Supplementary file 1 — Data S1. Compressed file containing the IGV screenshots for all the RetroTest exclusive insertions inspected in sample_21 and sample_28 WGS data, classified as true positives (TPs), false positives (FPs), and unconclusive. Both the tumor and normal BAM files were included in each screenshot. [file MOL2-19-3769-s003.zip › IGV_screenshots_illuminaWGS_TD2-RetroTest-exclusive_classified/PD0277a_retrotest_exclusive_IlluminaWGS/TPs/chr2_115701919-115702519.png]

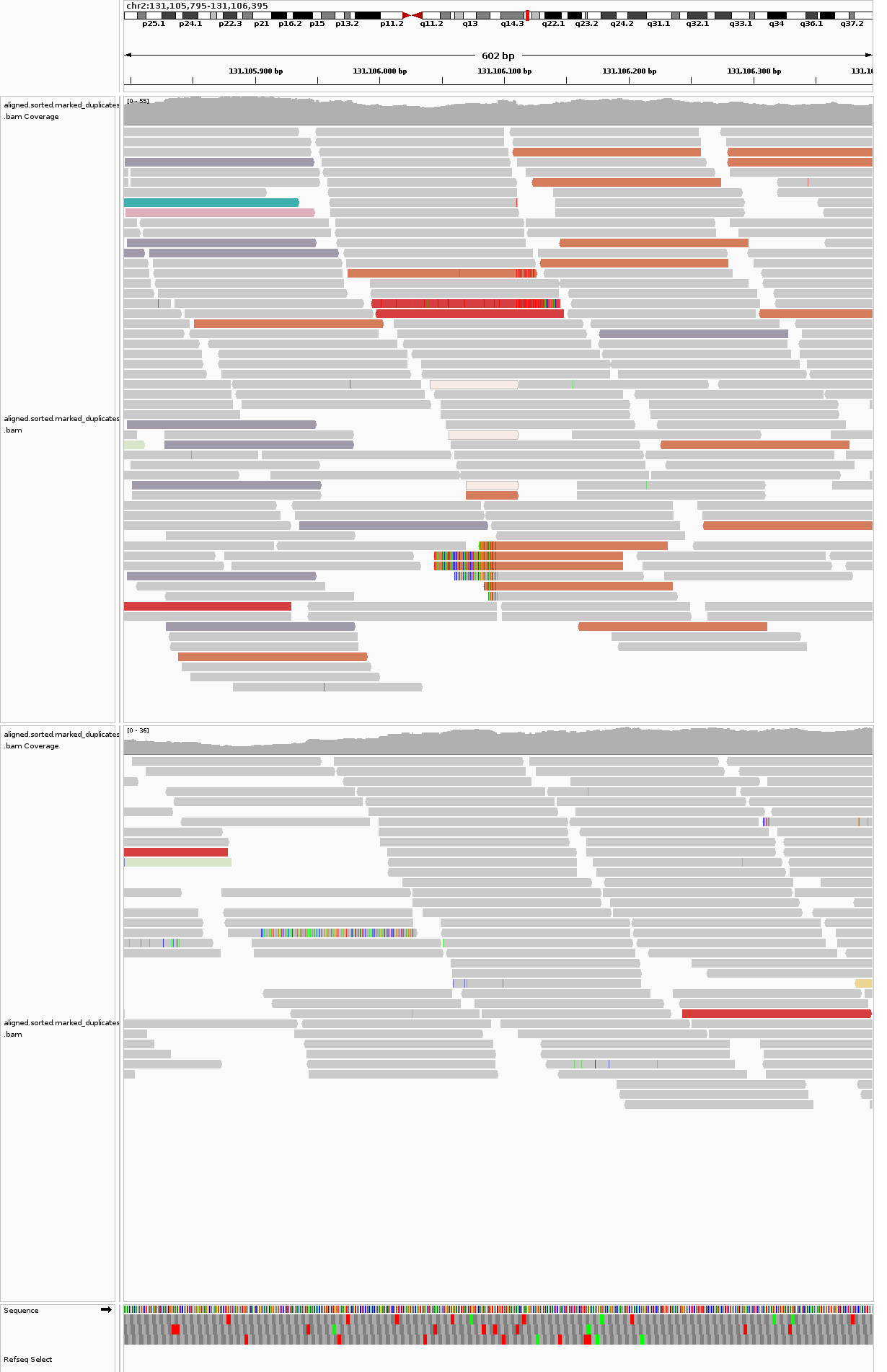

Supplement: Supplementary file 1 — Data S1. Compressed file containing the IGV screenshots for all the RetroTest exclusive insertions inspected in sample_21 and sample_28 WGS data, classified as true positives (TPs), false positives (FPs), and unconclusive. Both the tumor and normal BAM files were included in each screenshot. [file MOL2-19-3769-s003.zip › IGV_screenshots_illuminaWGS_TD2-RetroTest-exclusive_classified/PD0277a_retrotest_exclusive_IlluminaWGS/TPs/chr2_131105795-131106395.png]

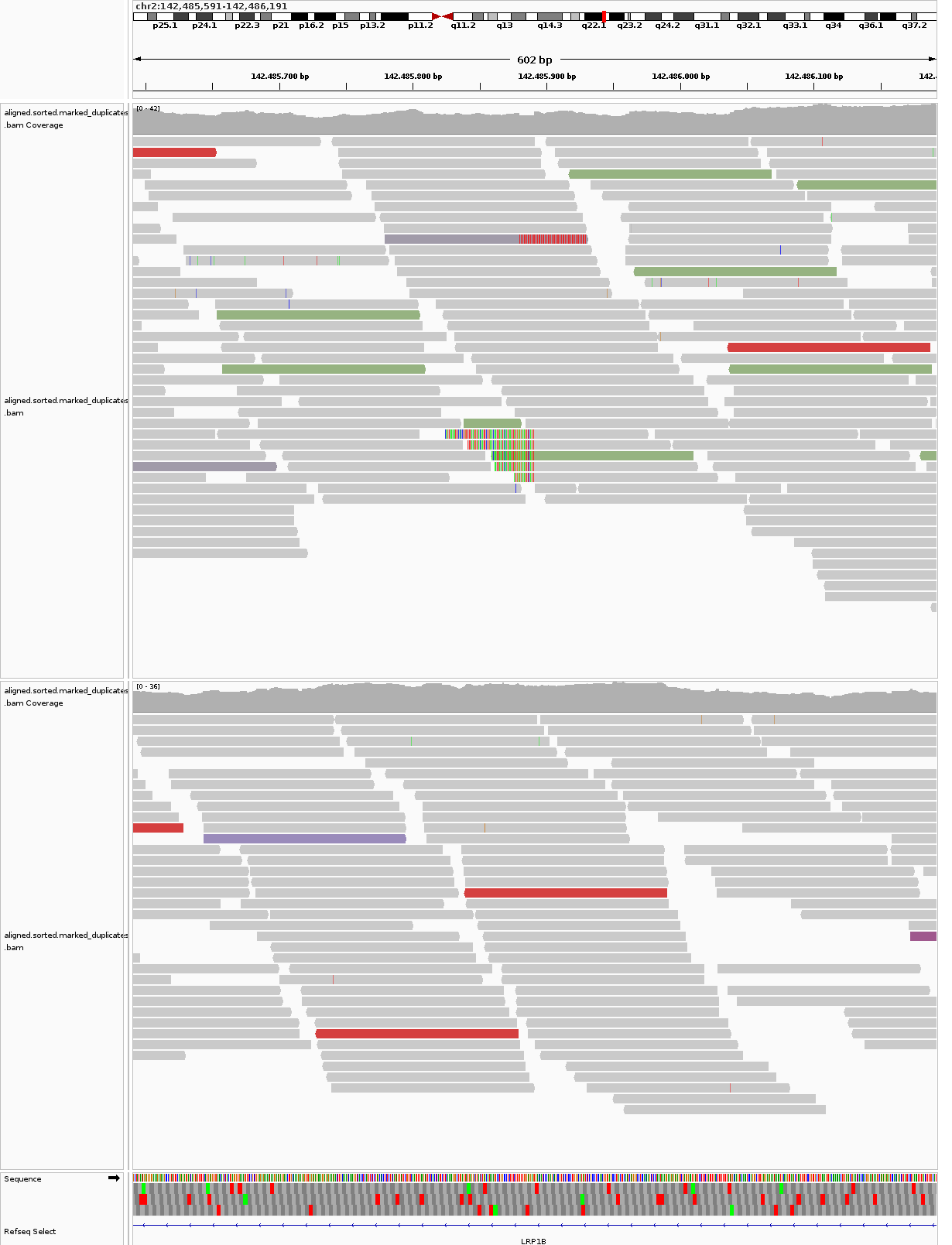

Supplement: Supplementary file 1 — Data S1. Compressed file containing the IGV screenshots for all the RetroTest exclusive insertions inspected in sample_21 and sample_28 WGS data, classified as true positives (TPs), false positives (FPs), and unconclusive. Both the tumor and normal BAM files were included in each screenshot. [file MOL2-19-3769-s003.zip › IGV_screenshots_illuminaWGS_TD2-RetroTest-exclusive_classified/PD0277a_retrotest_exclusive_IlluminaWGS/TPs/chr2_142485591-142486191.png]

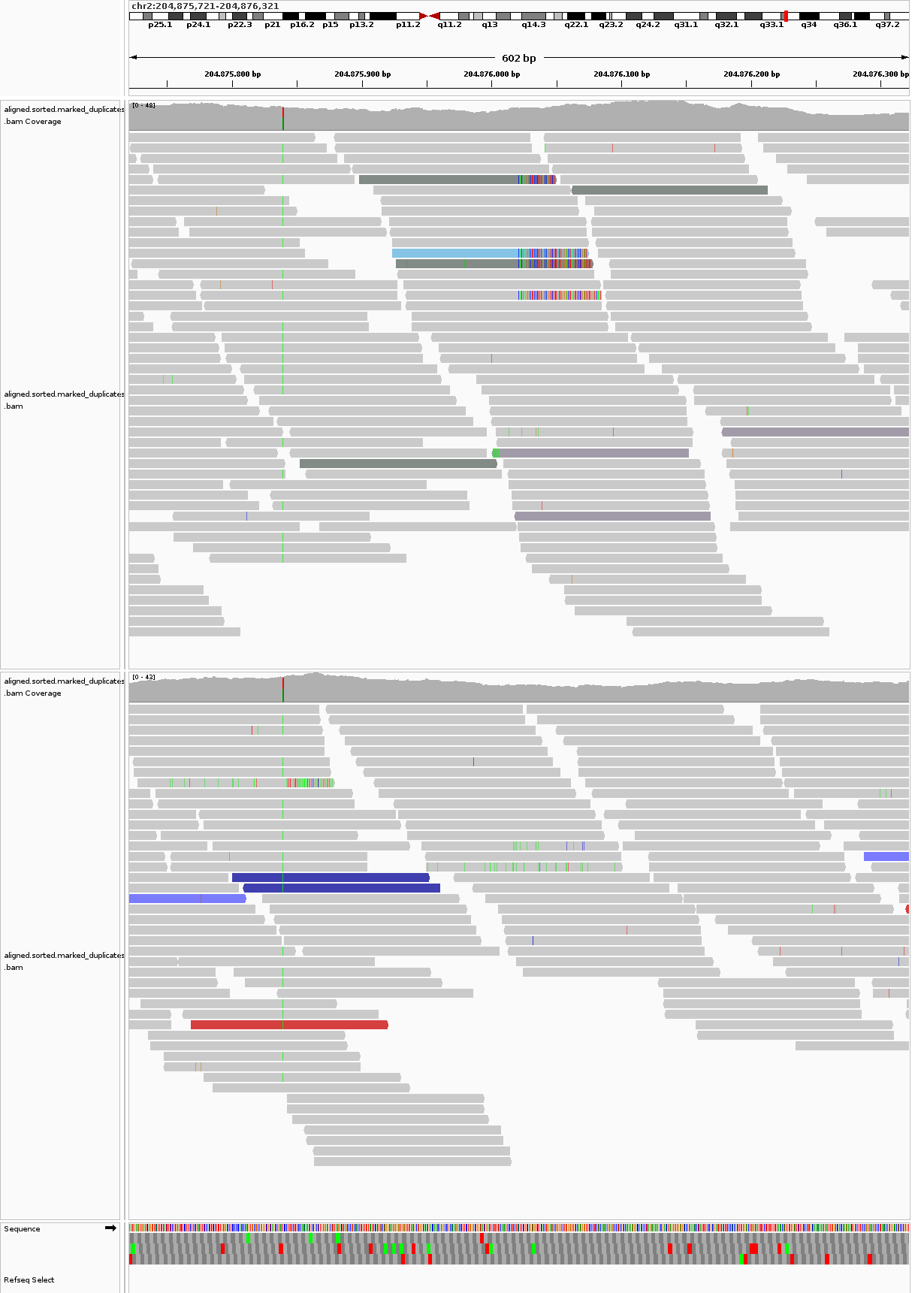

Supplement: Supplementary file 1 — Data S1. Compressed file containing the IGV screenshots for all the RetroTest exclusive insertions inspected in sample_21 and sample_28 WGS data, classified as true positives (TPs), false positives (FPs), and unconclusive. Both the tumor and normal BAM files were included in each screenshot. [file MOL2-19-3769-s003.zip › IGV_screenshots_illuminaWGS_TD2-RetroTest-exclusive_classified/PD0277a_retrotest_exclusive_IlluminaWGS/TPs/chr2_204875721-204876321.png]

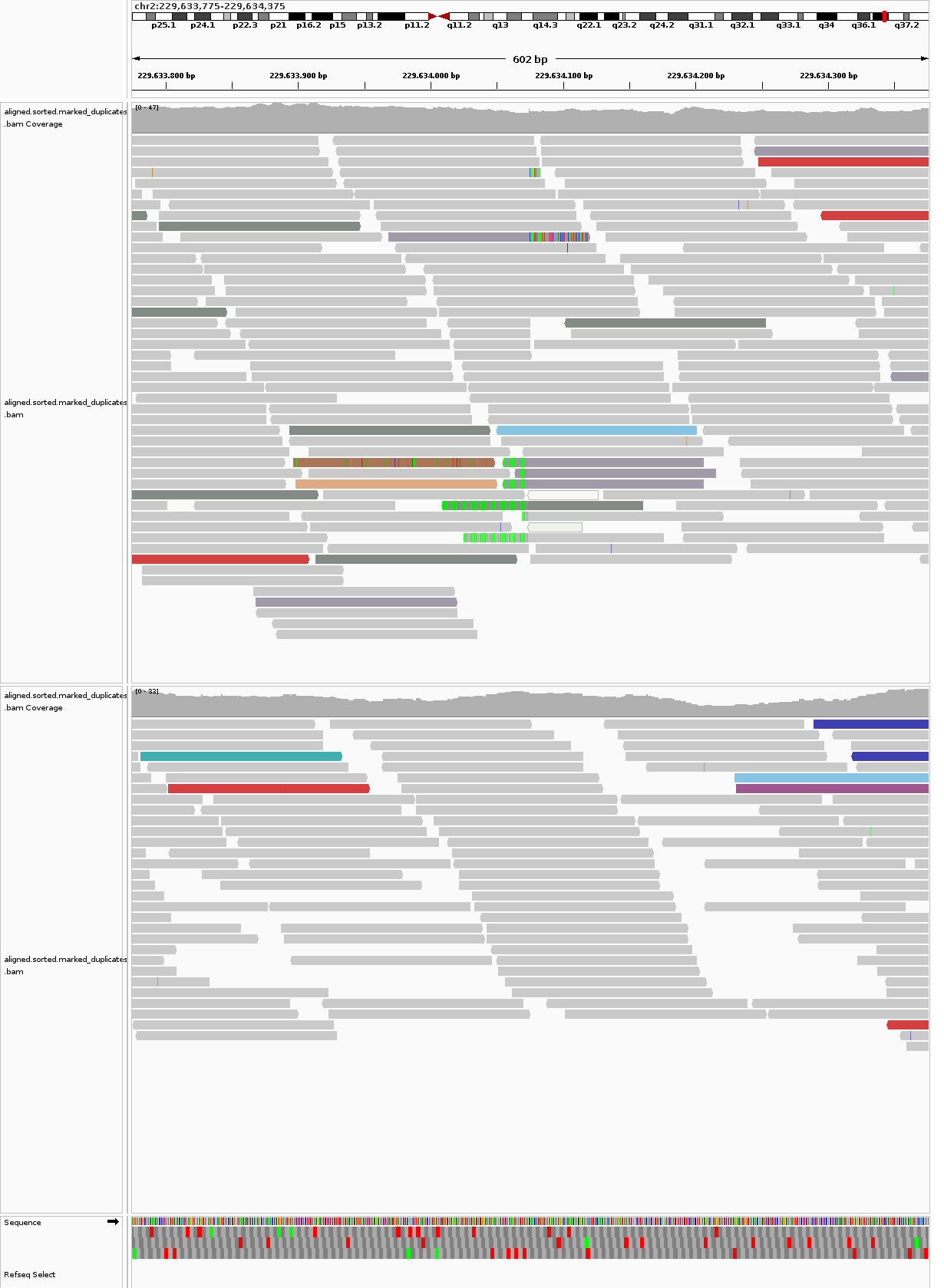

Supplement: Supplementary file 1 — Data S1. Compressed file containing the IGV screenshots for all the RetroTest exclusive insertions inspected in sample_21 and sample_28 WGS data, classified as true positives (TPs), false positives (FPs), and unconclusive. Both the tumor and normal BAM files were included in each screenshot. [file MOL2-19-3769-s003.zip › IGV_screenshots_illuminaWGS_TD2-RetroTest-exclusive_classified/PD0277a_retrotest_exclusive_IlluminaWGS/TPs/chr2_229633775-229634375.png]

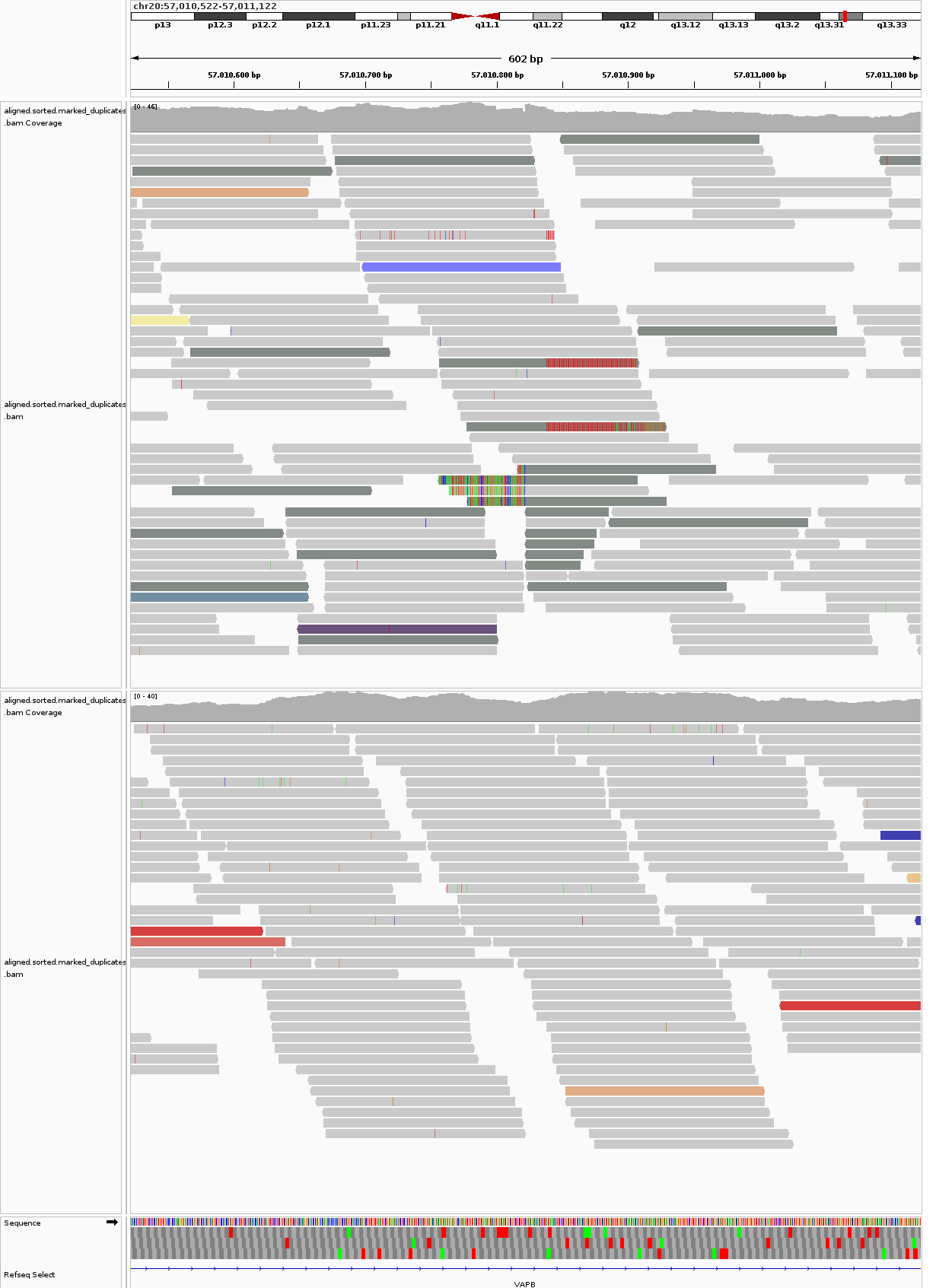

Supplement: Supplementary file 1 — Data S1. Compressed file containing the IGV screenshots for all the RetroTest exclusive insertions inspected in sample_21 and sample_28 WGS data, classified as true positives (TPs), false positives (FPs), and unconclusive. Both the tumor and normal BAM files were included in each screenshot. [file MOL2-19-3769-s003.zip › IGV_screenshots_illuminaWGS_TD2-RetroTest-exclusive_classified/PD0277a_retrotest_exclusive_IlluminaWGS/TPs/chr20_57010522-57011122.png]

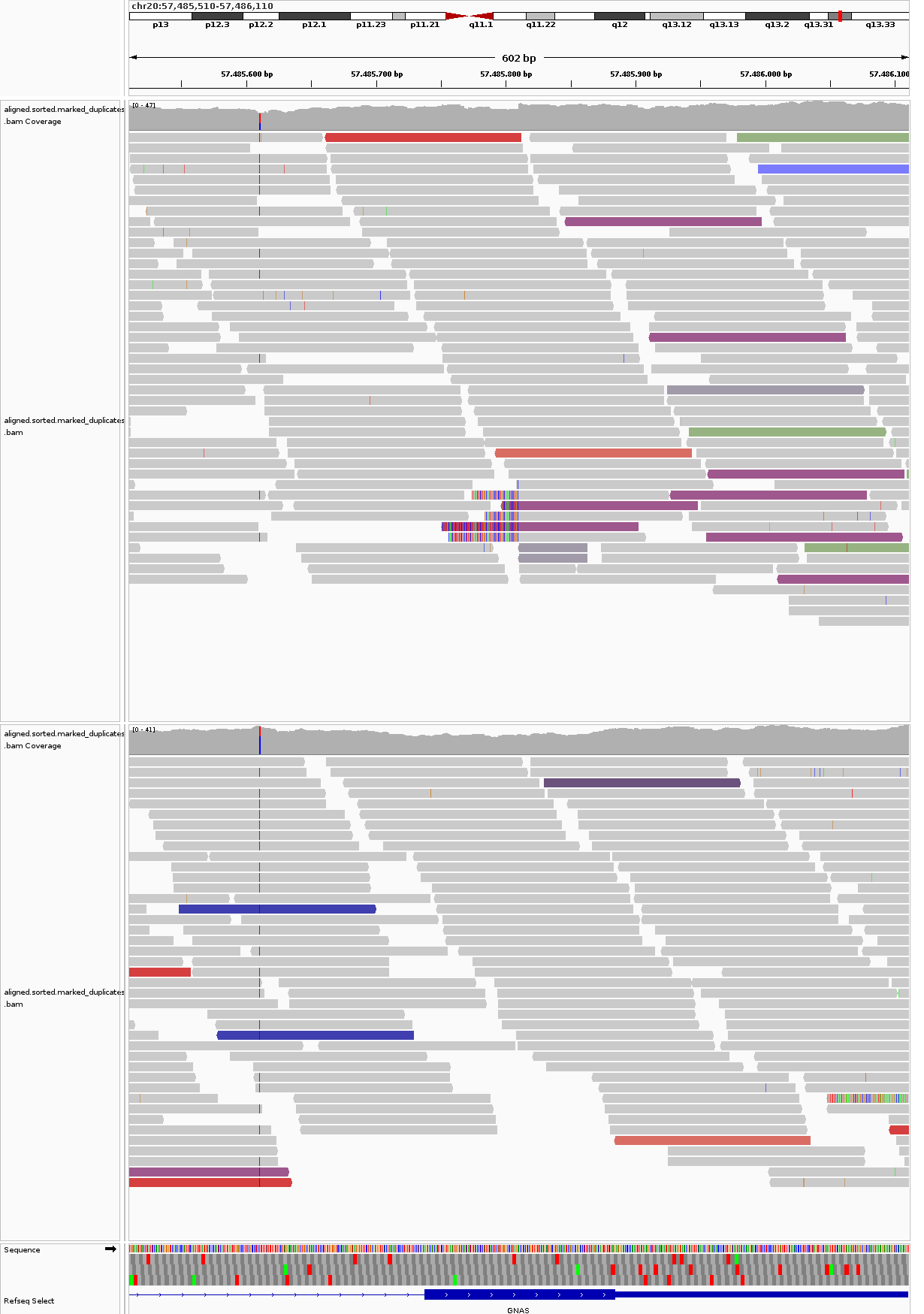

Supplement: Supplementary file 1 — Data S1. Compressed file containing the IGV screenshots for all the RetroTest exclusive insertions inspected in sample_21 and sample_28 WGS data, classified as true positives (TPs), false positives (FPs), and unconclusive. Both the tumor and normal BAM files were included in each screenshot. [file MOL2-19-3769-s003.zip › IGV_screenshots_illuminaWGS_TD2-RetroTest-exclusive_classified/PD0277a_retrotest_exclusive_IlluminaWGS/TPs/chr20_57485510-57486110.png]

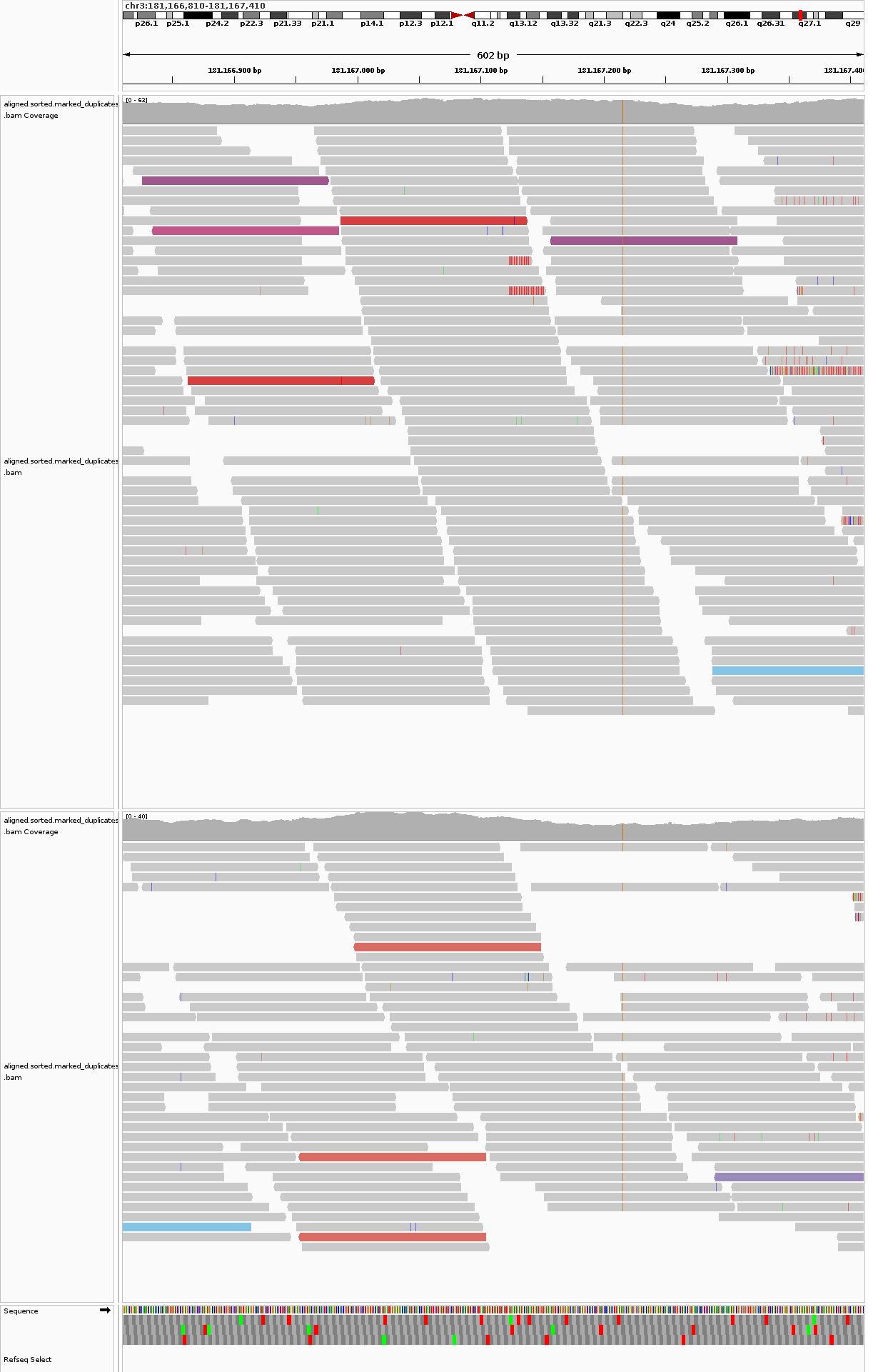

Supplement: Supplementary file 1 — Data S1. Compressed file containing the IGV screenshots for all the RetroTest exclusive insertions inspected in sample_21 and sample_28 WGS data, classified as true positives (TPs), false positives (FPs), and unconclusive. Both the tumor and normal BAM files were included in each screenshot. [file MOL2-19-3769-s003.zip › IGV_screenshots_illuminaWGS_TD2-RetroTest-exclusive_classified/PD0277a_retrotest_exclusive_IlluminaWGS/TPs/chr3_181166810-181167410.png]

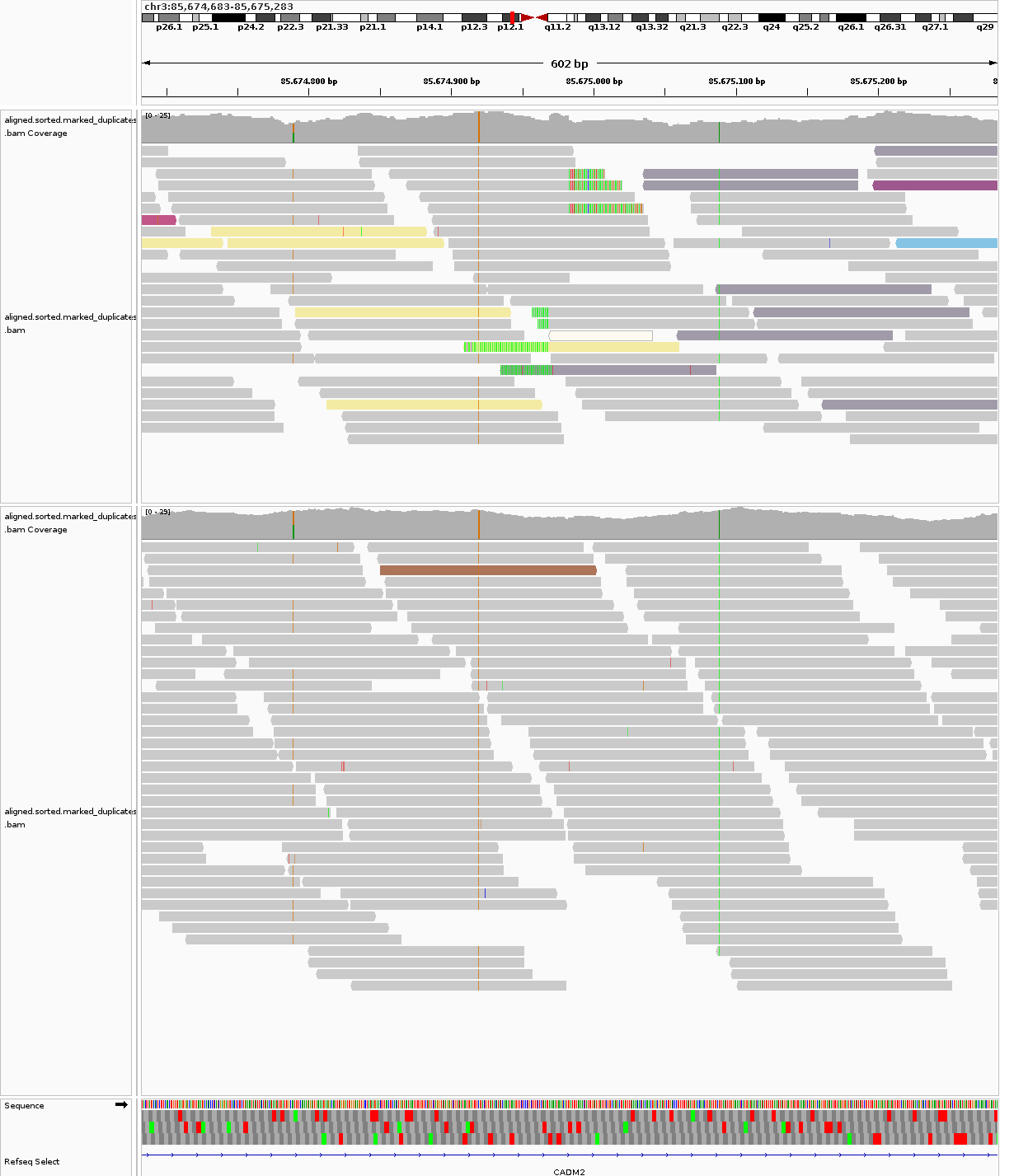

Supplement: Supplementary file 1 — Data S1. Compressed file containing the IGV screenshots for all the RetroTest exclusive insertions inspected in sample_21 and sample_28 WGS data, classified as true positives (TPs), false positives (FPs), and unconclusive. Both the tumor and normal BAM files were included in each screenshot. [file MOL2-19-3769-s003.zip › IGV_screenshots_illuminaWGS_TD2-RetroTest-exclusive_classified/PD0277a_retrotest_exclusive_IlluminaWGS/TPs/chr3_85674683-85675283.png]

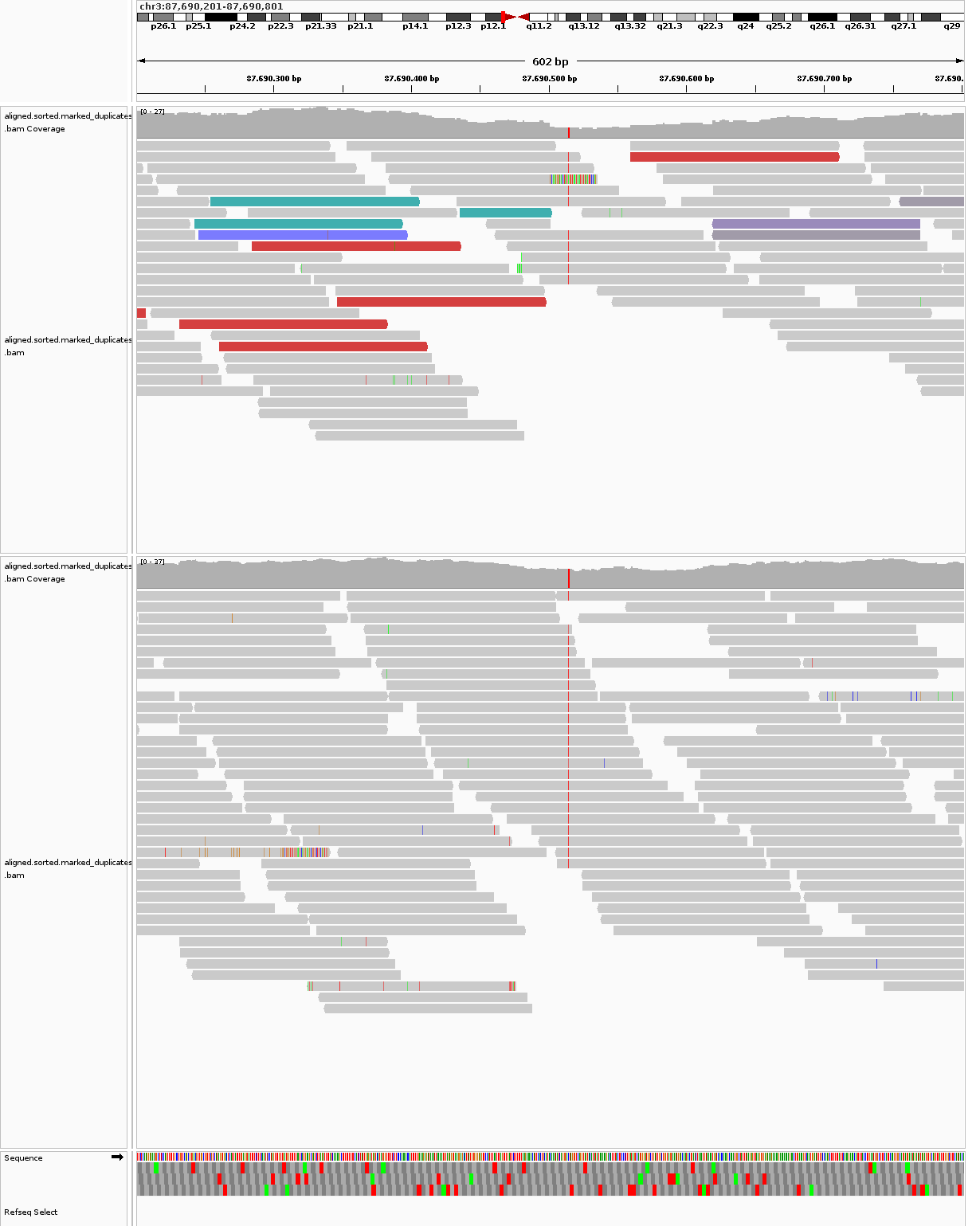

Supplement: Supplementary file 1 — Data S1. Compressed file containing the IGV screenshots for all the RetroTest exclusive insertions inspected in sample_21 and sample_28 WGS data, classified as true positives (TPs), false positives (FPs), and unconclusive. Both the tumor and normal BAM files were included in each screenshot. [file MOL2-19-3769-s003.zip › IGV_screenshots_illuminaWGS_TD2-RetroTest-exclusive_classified/PD0277a_retrotest_exclusive_IlluminaWGS/TPs/chr3_87690201-87690801.png]

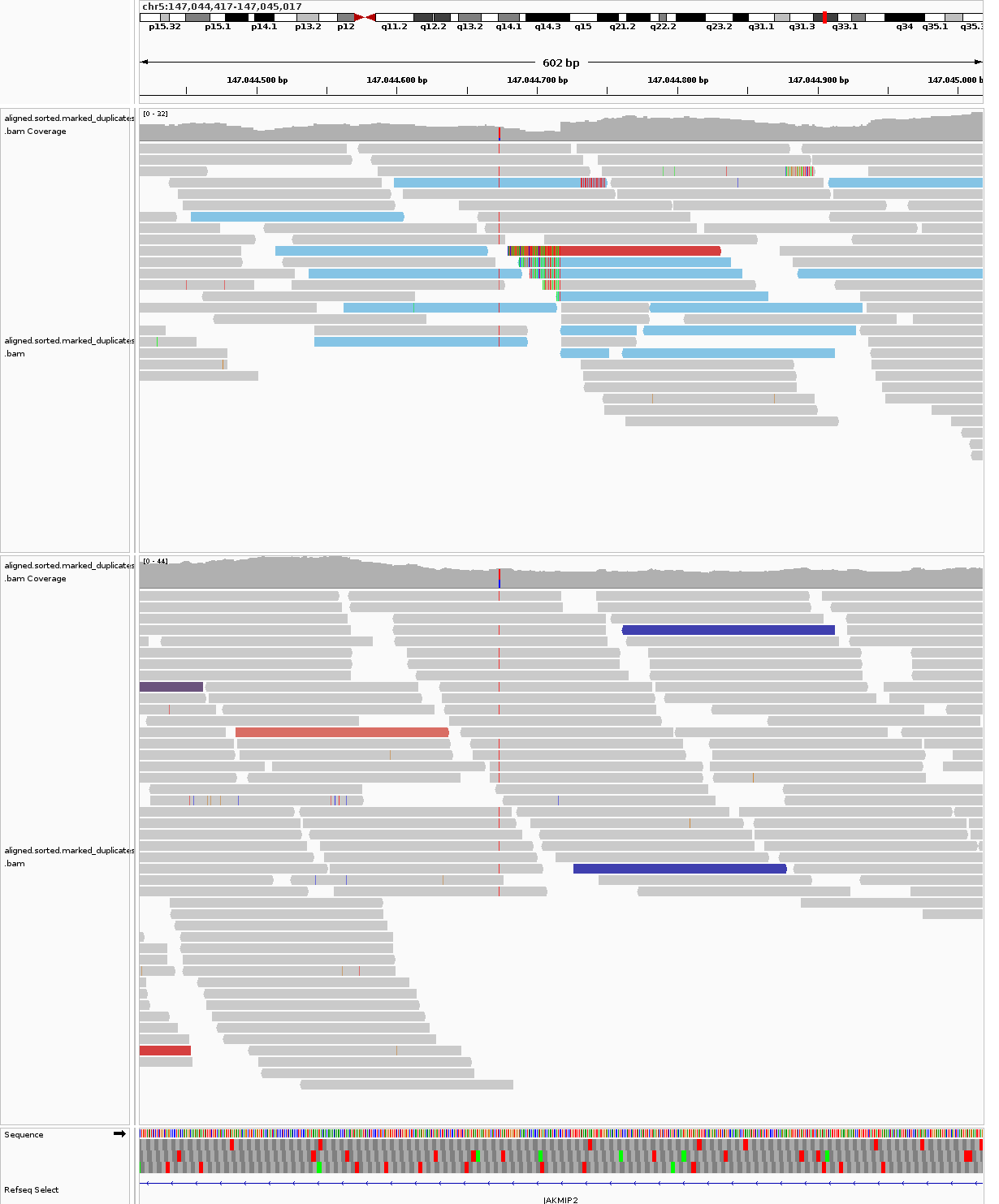

Supplement: Supplementary file 1 — Data S1. Compressed file containing the IGV screenshots for all the RetroTest exclusive insertions inspected in sample_21 and sample_28 WGS data, classified as true positives (TPs), false positives (FPs), and unconclusive. Both the tumor and normal BAM files were included in each screenshot. [file MOL2-19-3769-s003.zip › IGV_screenshots_illuminaWGS_TD2-RetroTest-exclusive_classified/PD0277a_retrotest_exclusive_IlluminaWGS/TPs/chr5_147044417-147045017.png]

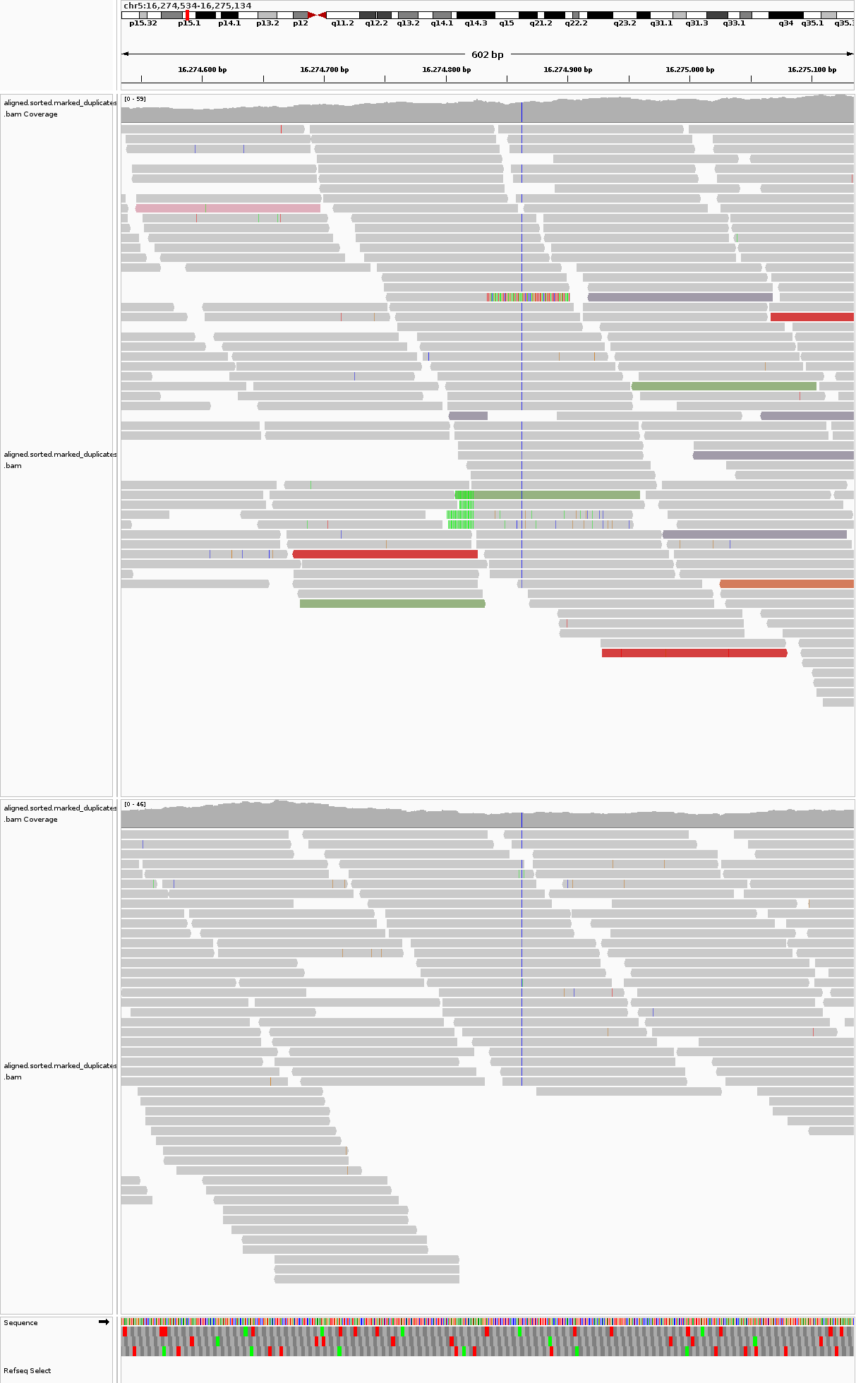

Supplement: Supplementary file 1 — Data S1. Compressed file containing the IGV screenshots for all the RetroTest exclusive insertions inspected in sample_21 and sample_28 WGS data, classified as true positives (TPs), false positives (FPs), and unconclusive. Both the tumor and normal BAM files were included in each screenshot. [file MOL2-19-3769-s003.zip › IGV_screenshots_illuminaWGS_TD2-RetroTest-exclusive_classified/PD0277a_retrotest_exclusive_IlluminaWGS/TPs/chr5_16274534-16275134.png]

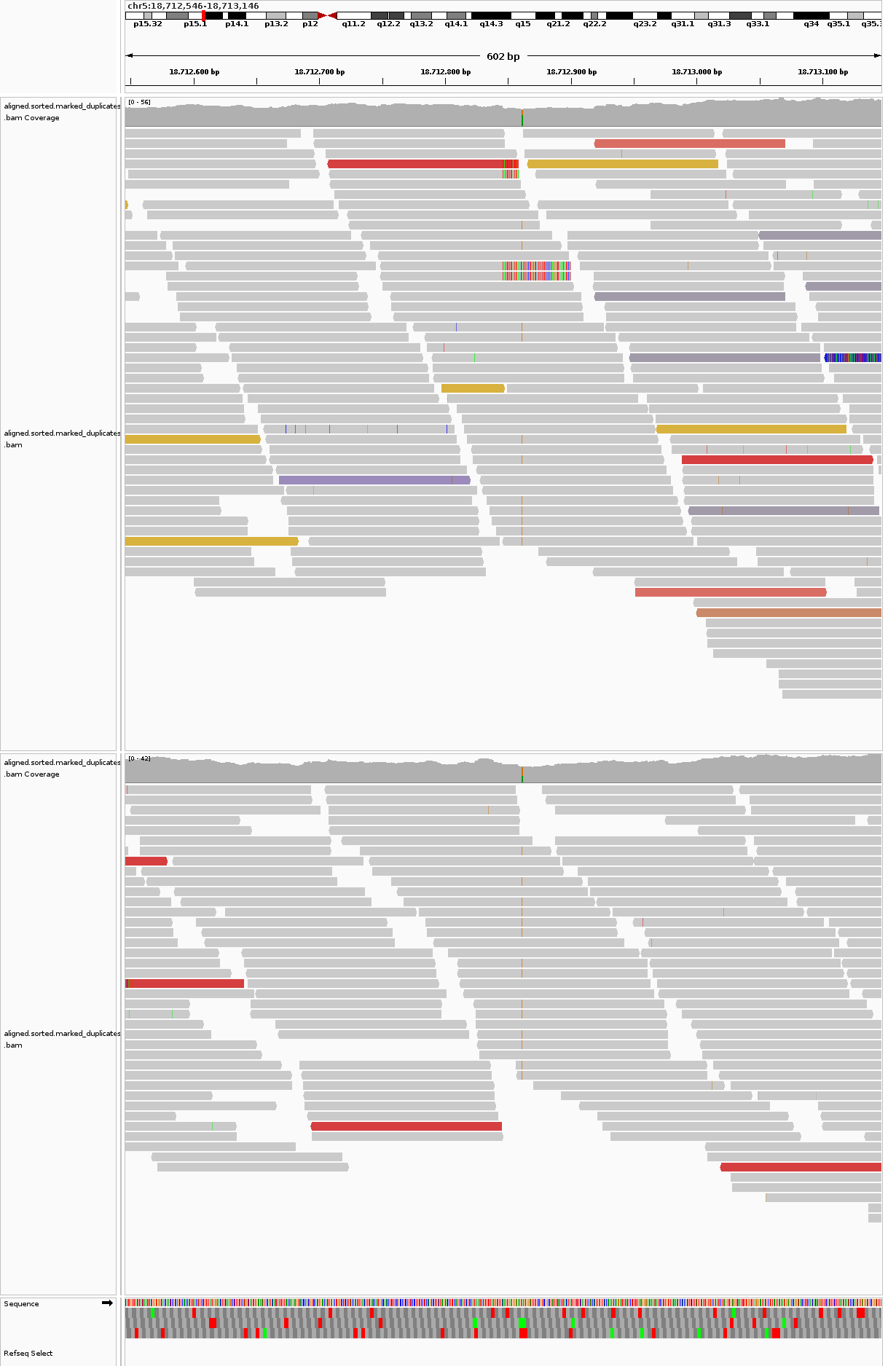

Supplement: Supplementary file 1 — Data S1. Compressed file containing the IGV screenshots for all the RetroTest exclusive insertions inspected in sample_21 and sample_28 WGS data, classified as true positives (TPs), false positives (FPs), and unconclusive. Both the tumor and normal BAM files were included in each screenshot. [file MOL2-19-3769-s003.zip › IGV_screenshots_illuminaWGS_TD2-RetroTest-exclusive_classified/PD0277a_retrotest_exclusive_IlluminaWGS/TPs/chr5_18712546-18713146.png]

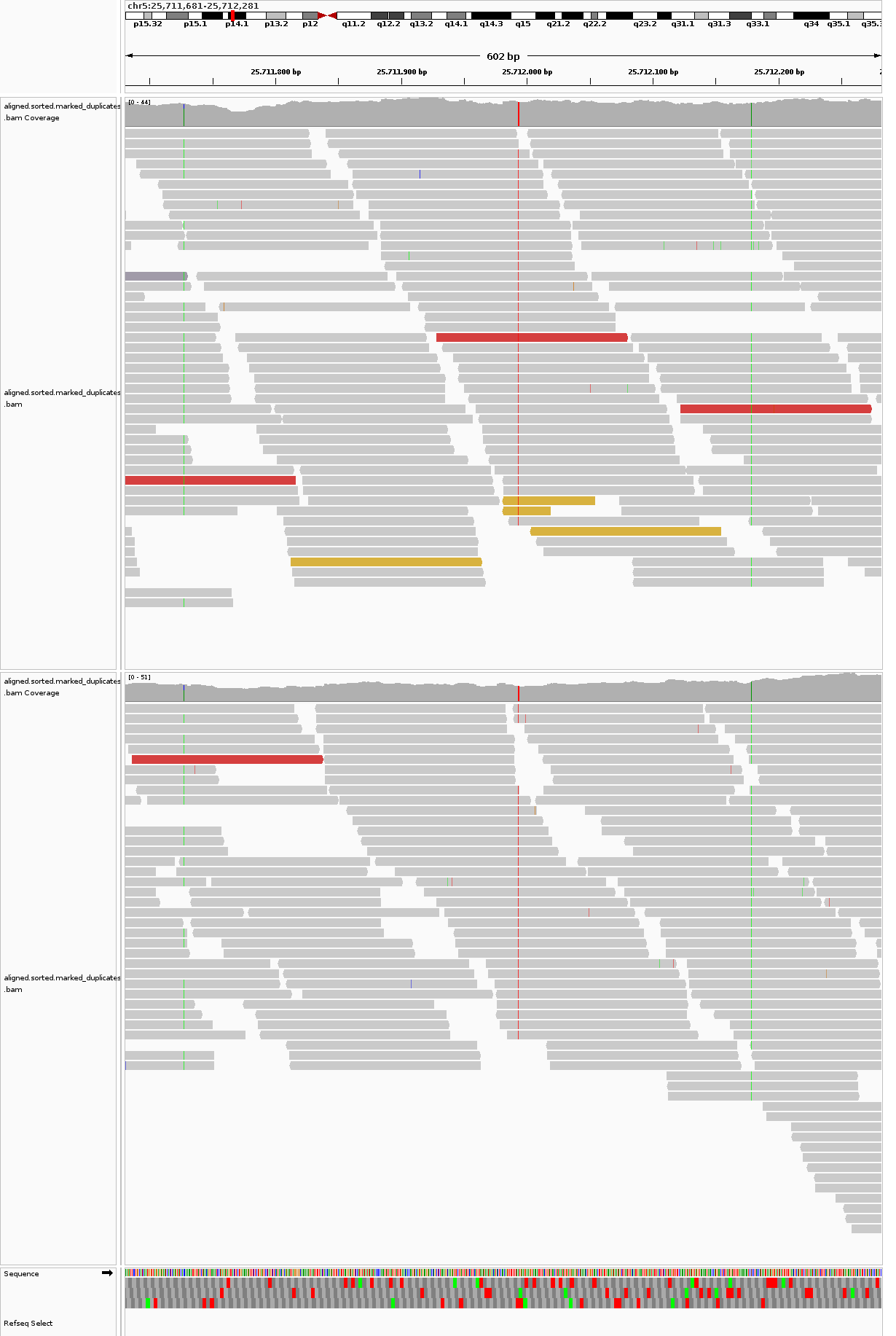

Supplement: Supplementary file 1 — Data S1. Compressed file containing the IGV screenshots for all the RetroTest exclusive insertions inspected in sample_21 and sample_28 WGS data, classified as true positives (TPs), false positives (FPs), and unconclusive. Both the tumor and normal BAM files were included in each screenshot. [file MOL2-19-3769-s003.zip › IGV_screenshots_illuminaWGS_TD2-RetroTest-exclusive_classified/PD0277a_retrotest_exclusive_IlluminaWGS/TPs/chr5_25711681-25712281.png]

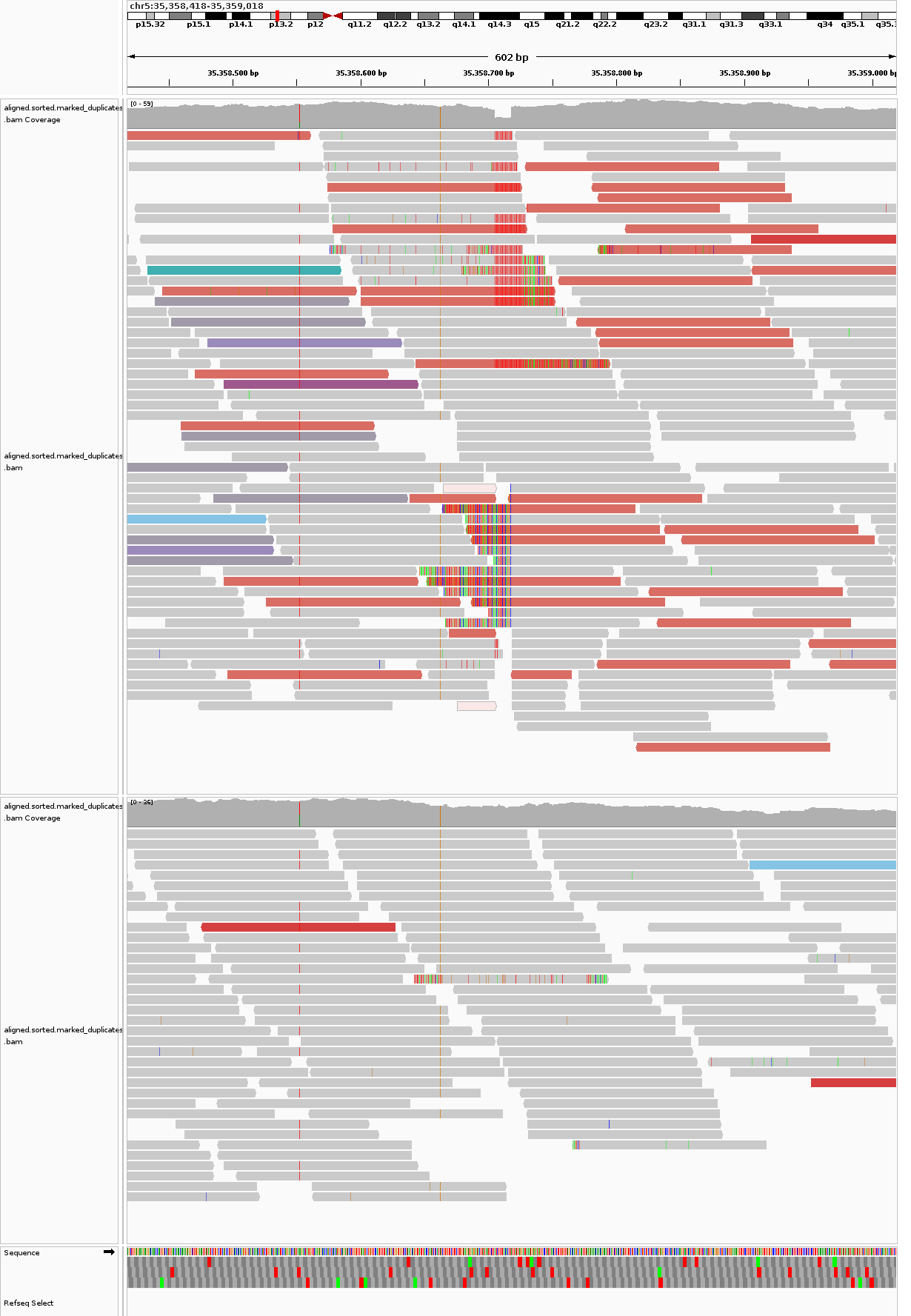

Supplement: Supplementary file 1 — Data S1. Compressed file containing the IGV screenshots for all the RetroTest exclusive insertions inspected in sample_21 and sample_28 WGS data, classified as true positives (TPs), false positives (FPs), and unconclusive. Both the tumor and normal BAM files were included in each screenshot. [file MOL2-19-3769-s003.zip › IGV_screenshots_illuminaWGS_TD2-RetroTest-exclusive_classified/PD0277a_retrotest_exclusive_IlluminaWGS/TPs/chr5_35358418-35359018.png]

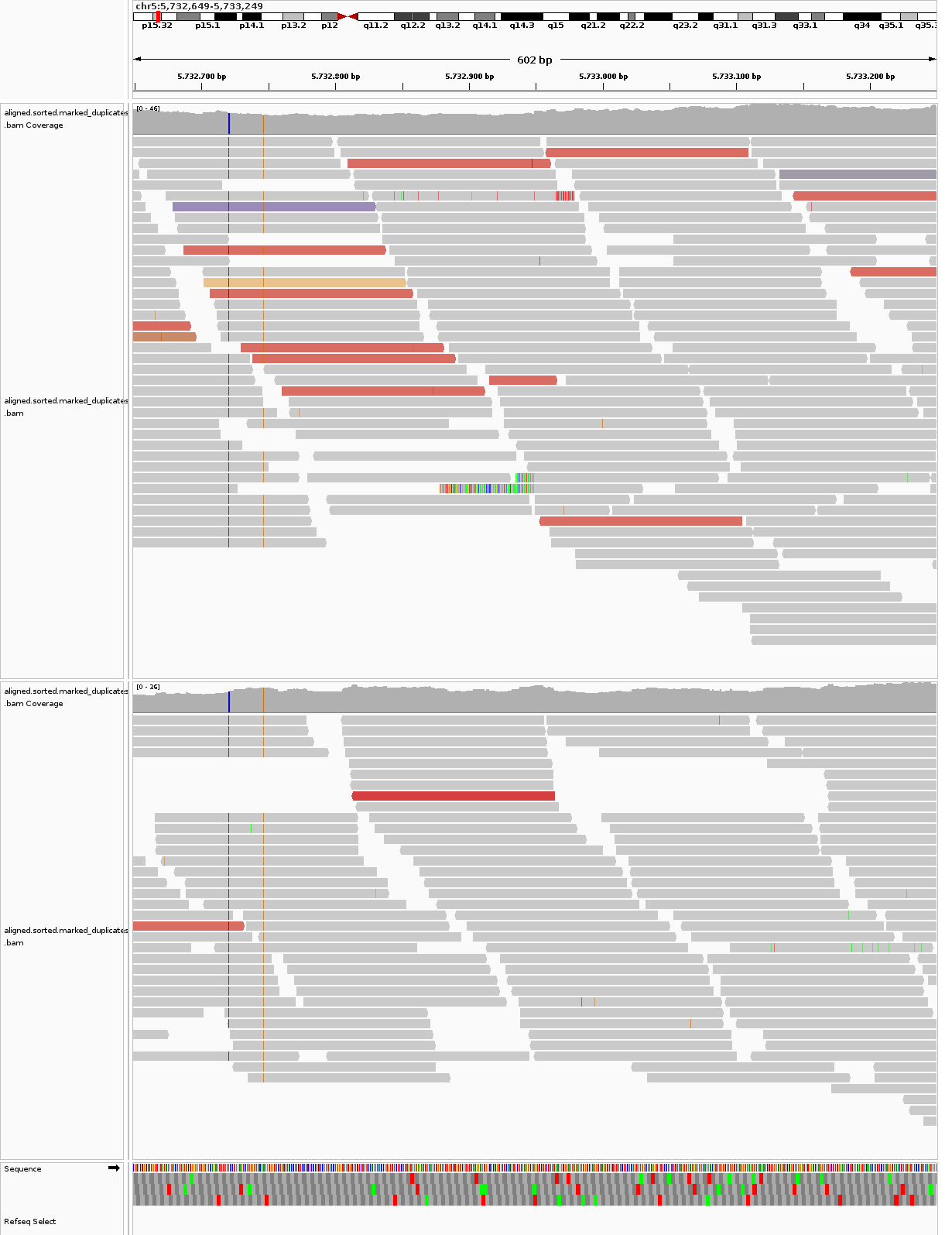

Supplement: Supplementary file 1 — Data S1. Compressed file containing the IGV screenshots for all the RetroTest exclusive insertions inspected in sample_21 and sample_28 WGS data, classified as true positives (TPs), false positives (FPs), and unconclusive. Both the tumor and normal BAM files were included in each screenshot. [file MOL2-19-3769-s003.zip › IGV_screenshots_illuminaWGS_TD2-RetroTest-exclusive_classified/PD0277a_retrotest_exclusive_IlluminaWGS/TPs/chr5_5732649-5733249.png]

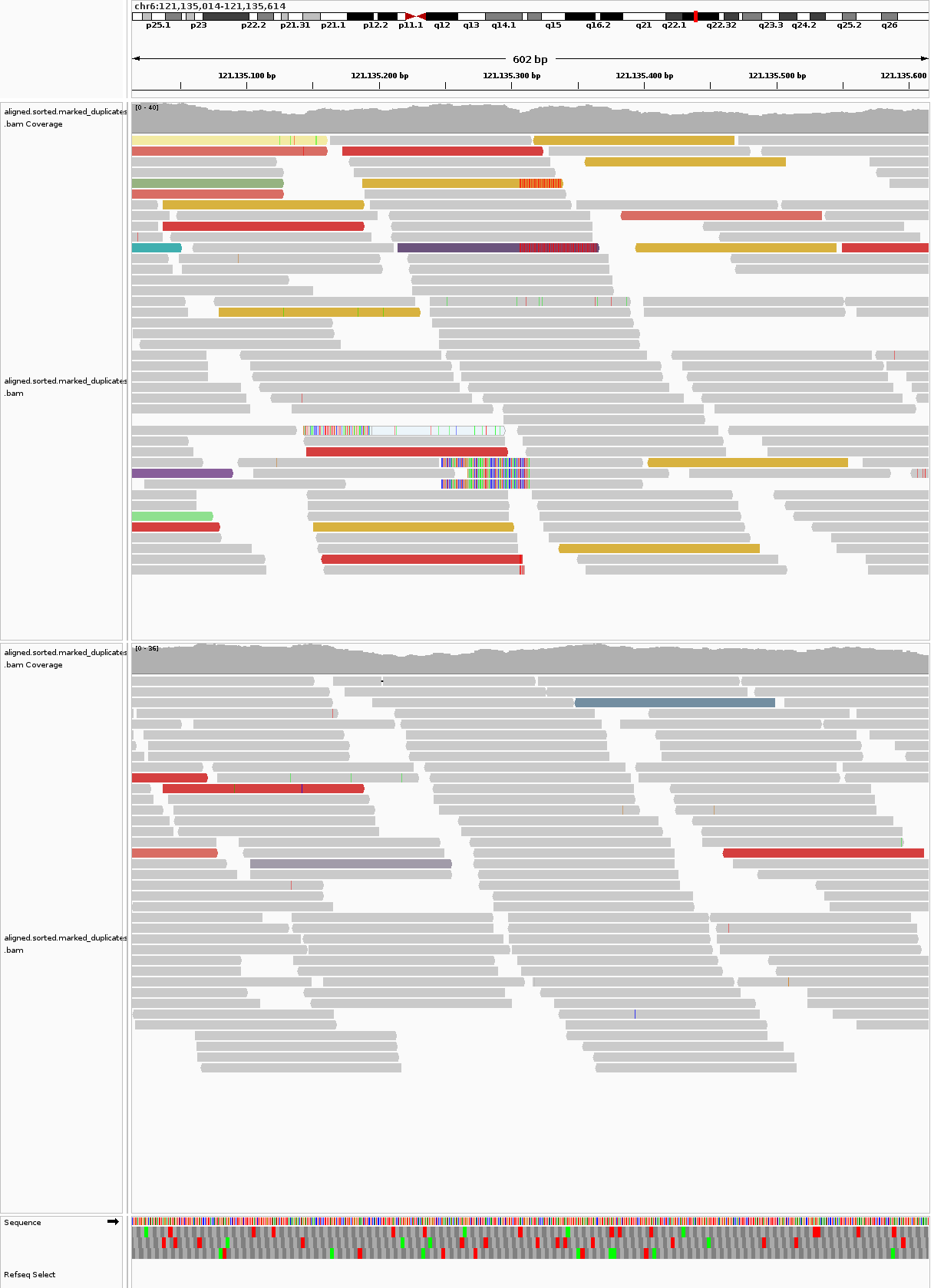

Supplement: Supplementary file 1 — Data S1. Compressed file containing the IGV screenshots for all the RetroTest exclusive insertions inspected in sample_21 and sample_28 WGS data, classified as true positives (TPs), false positives (FPs), and unconclusive. Both the tumor and normal BAM files were included in each screenshot. [file MOL2-19-3769-s003.zip › IGV_screenshots_illuminaWGS_TD2-RetroTest-exclusive_classified/PD0277a_retrotest_exclusive_IlluminaWGS/TPs/chr6_121135014-121135614.png]

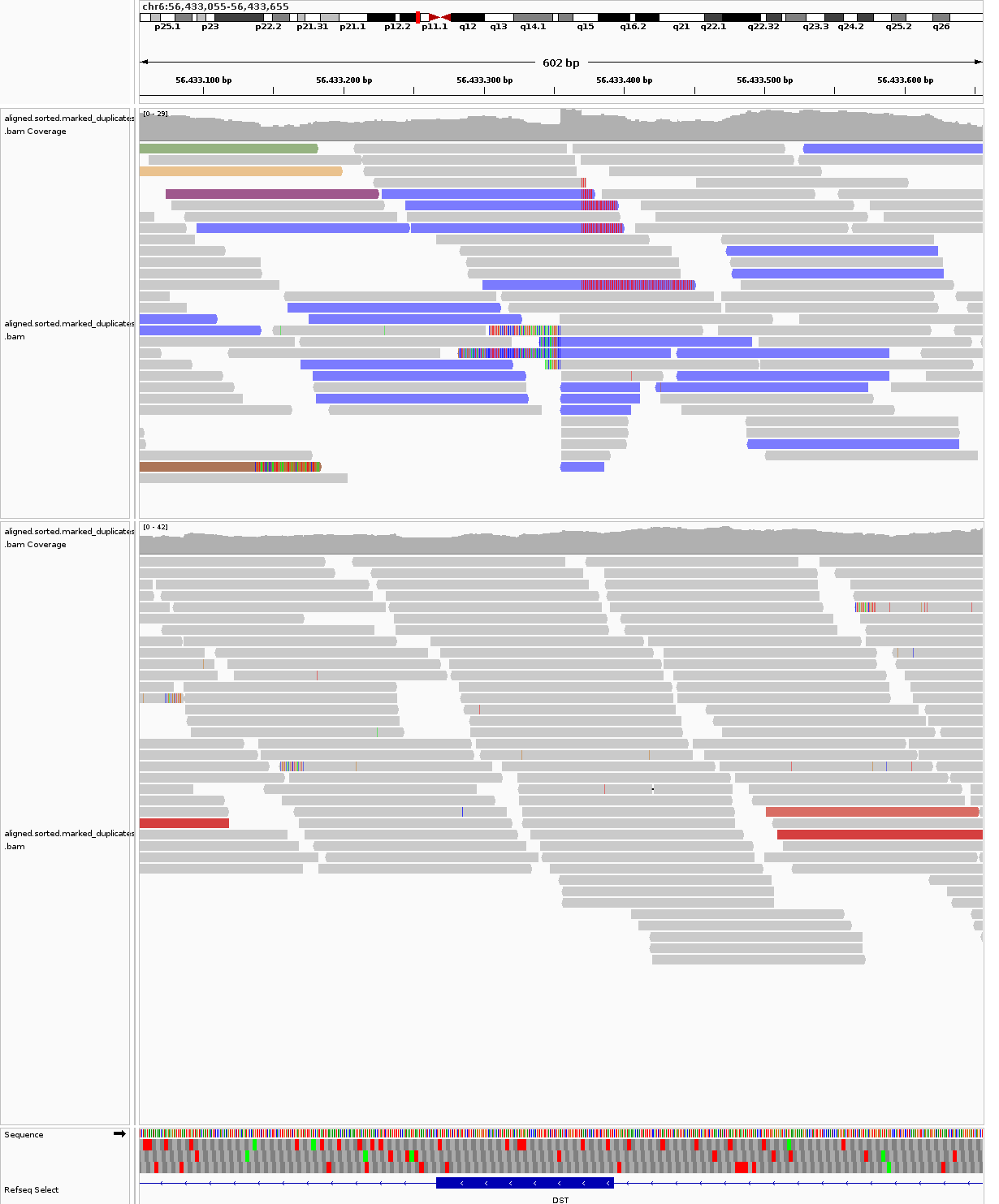

Supplement: Supplementary file 1 — Data S1. Compressed file containing the IGV screenshots for all the RetroTest exclusive insertions inspected in sample_21 and sample_28 WGS data, classified as true positives (TPs), false positives (FPs), and unconclusive. Both the tumor and normal BAM files were included in each screenshot. [file MOL2-19-3769-s003.zip › IGV_screenshots_illuminaWGS_TD2-RetroTest-exclusive_classified/PD0277a_retrotest_exclusive_IlluminaWGS/TPs/chr6_56433055-56433655.png]

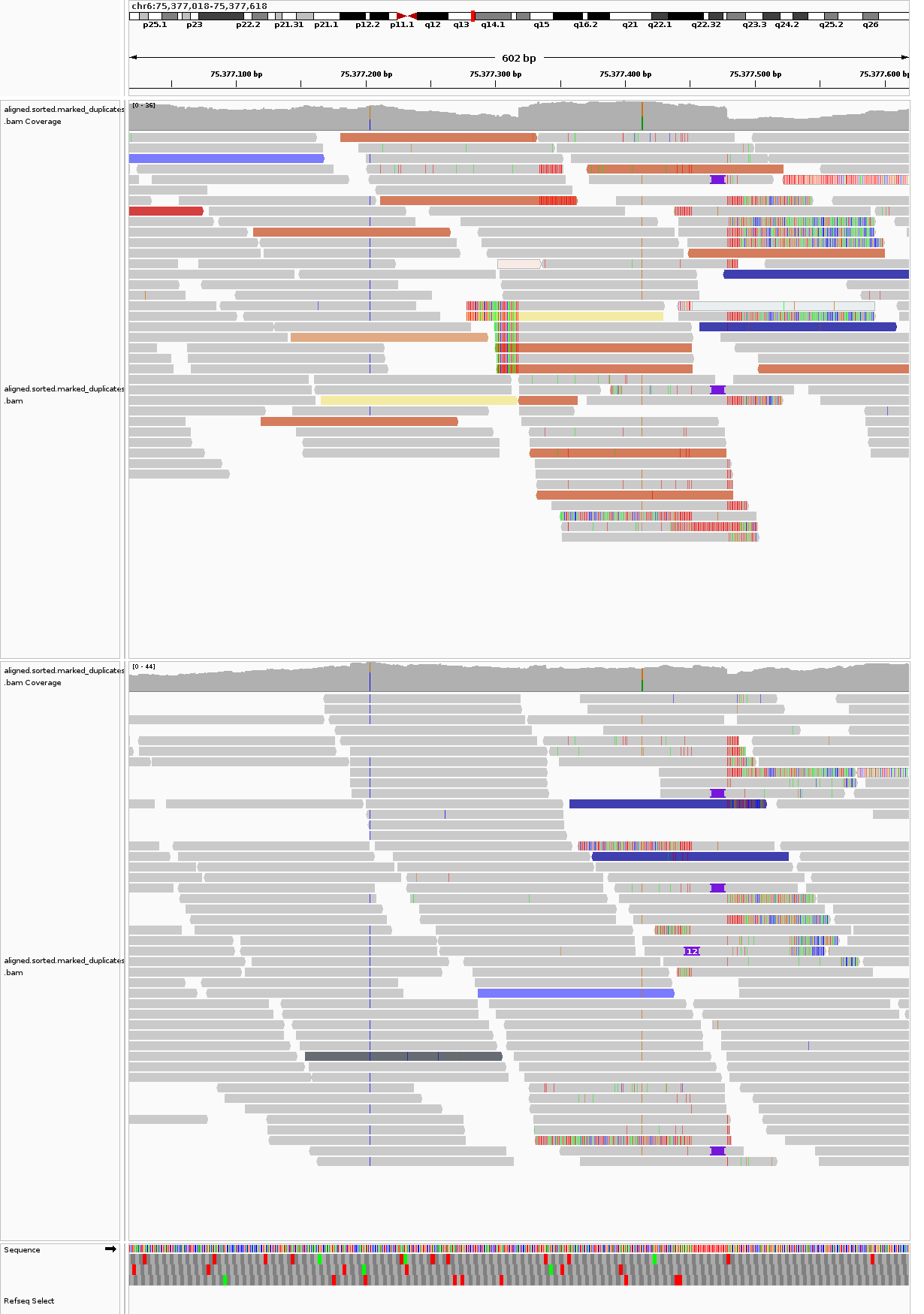

Supplement: Supplementary file 1 — Data S1. Compressed file containing the IGV screenshots for all the RetroTest exclusive insertions inspected in sample_21 and sample_28 WGS data, classified as true positives (TPs), false positives (FPs), and unconclusive. Both the tumor and normal BAM files were included in each screenshot. [file MOL2-19-3769-s003.zip › IGV_screenshots_illuminaWGS_TD2-RetroTest-exclusive_classified/PD0277a_retrotest_exclusive_IlluminaWGS/TPs/chr6_75377018-75377618.png]

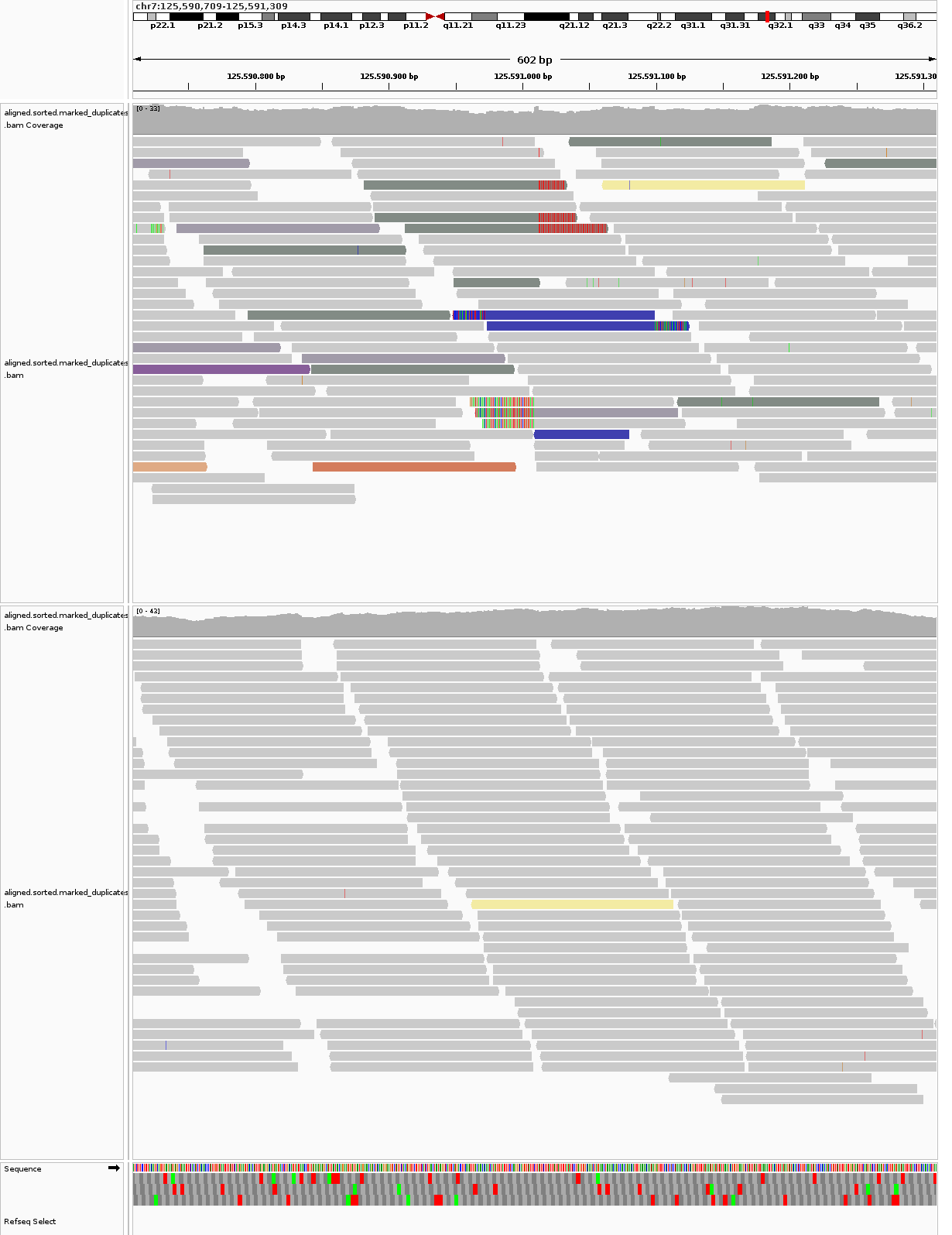

Supplement: Supplementary file 1 — Data S1. Compressed file containing the IGV screenshots for all the RetroTest exclusive insertions inspected in sample_21 and sample_28 WGS data, classified as true positives (TPs), false positives (FPs), and unconclusive. Both the tumor and normal BAM files were included in each screenshot. [file MOL2-19-3769-s003.zip › IGV_screenshots_illuminaWGS_TD2-RetroTest-exclusive_classified/PD0277a_retrotest_exclusive_IlluminaWGS/TPs/chr7_125590709-125591309.png]

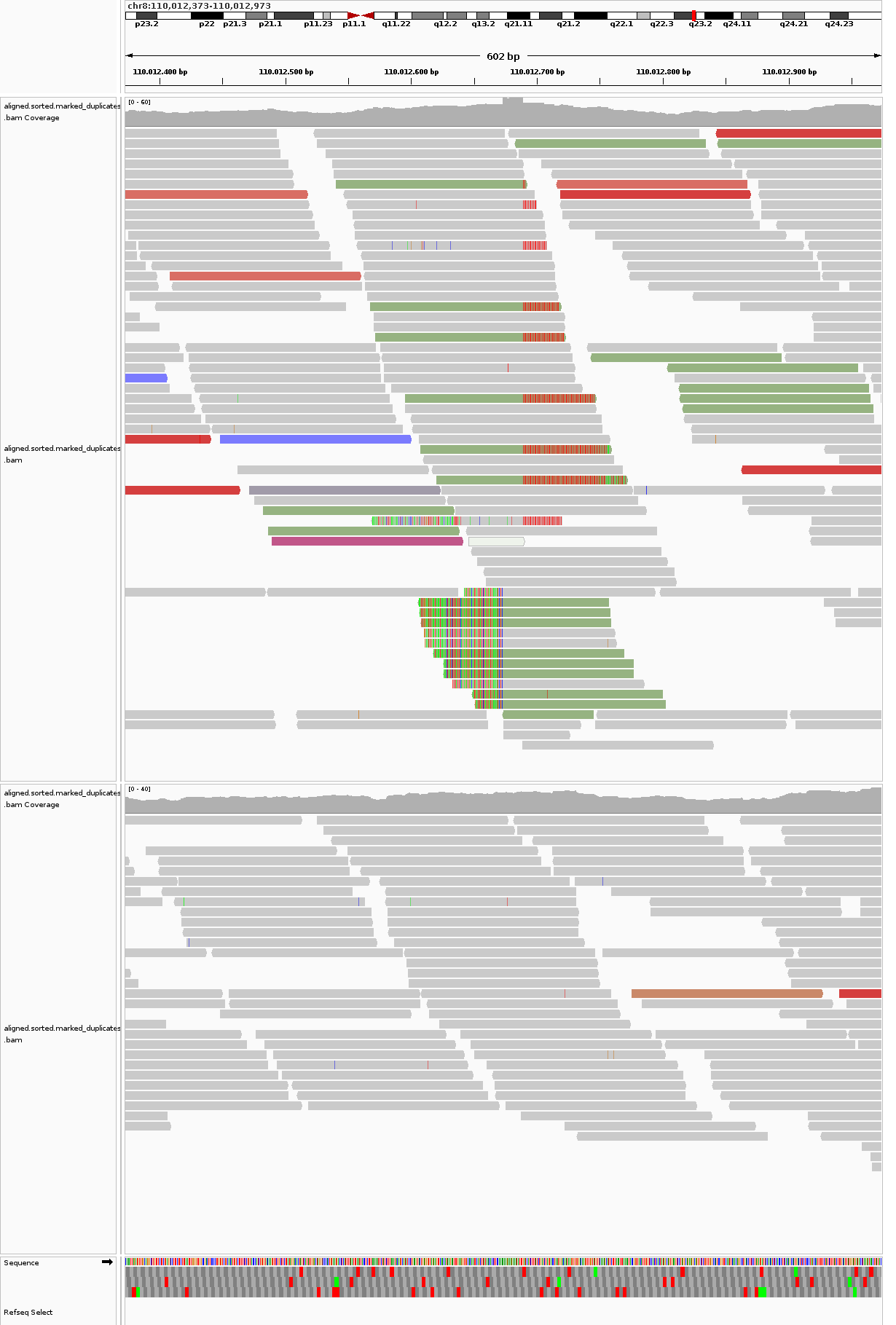

Supplement: Supplementary file 1 — Data S1. Compressed file containing the IGV screenshots for all the RetroTest exclusive insertions inspected in sample_21 and sample_28 WGS data, classified as true positives (TPs), false positives (FPs), and unconclusive. Both the tumor and normal BAM files were included in each screenshot. [file MOL2-19-3769-s003.zip › IGV_screenshots_illuminaWGS_TD2-RetroTest-exclusive_classified/PD0277a_retrotest_exclusive_IlluminaWGS/TPs/chr8_110012373-110012973.png]

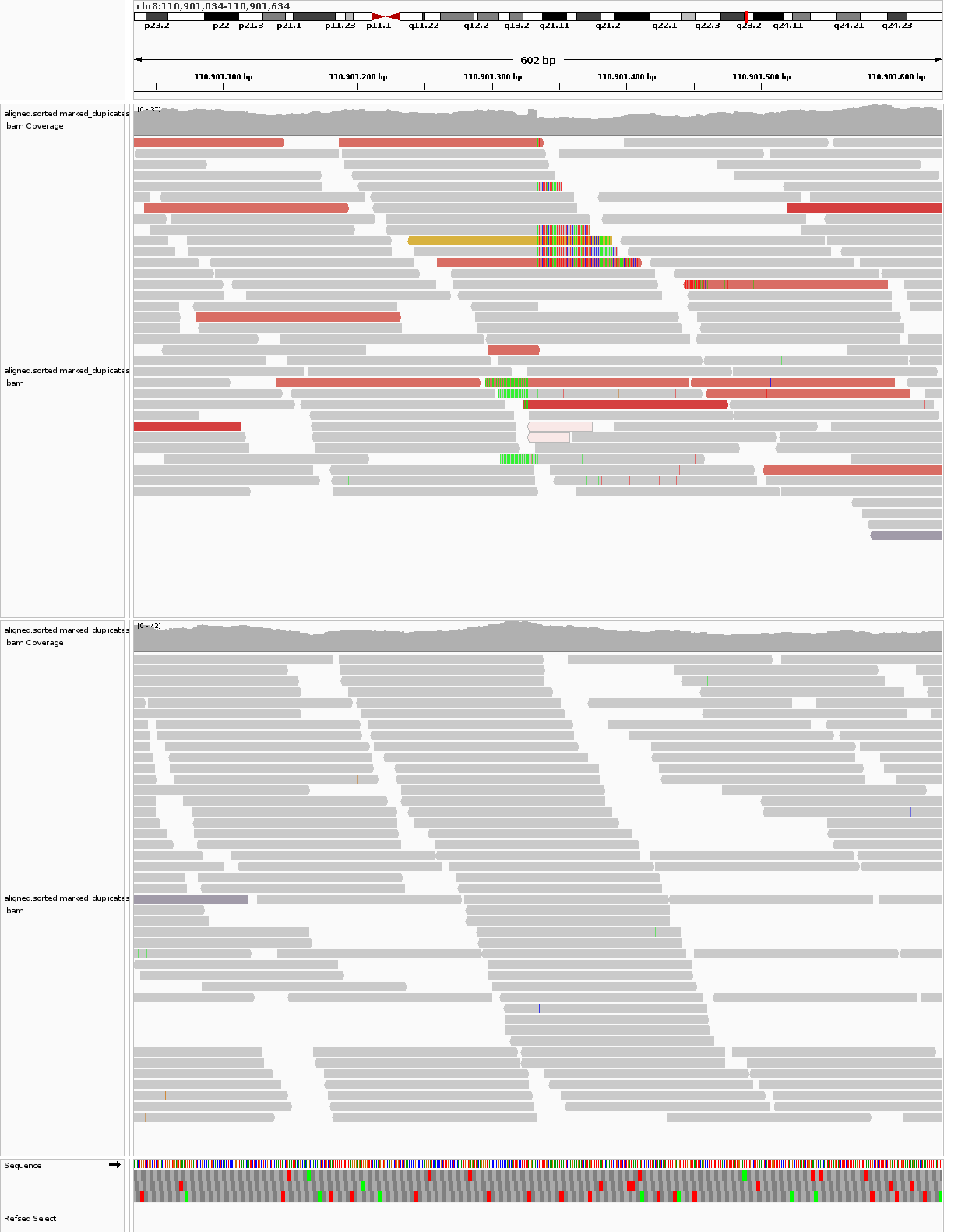

Supplement: Supplementary file 1 — Data S1. Compressed file containing the IGV screenshots for all the RetroTest exclusive insertions inspected in sample_21 and sample_28 WGS data, classified as true positives (TPs), false positives (FPs), and unconclusive. Both the tumor and normal BAM files were included in each screenshot. [file MOL2-19-3769-s003.zip › IGV_screenshots_illuminaWGS_TD2-RetroTest-exclusive_classified/PD0277a_retrotest_exclusive_IlluminaWGS/TPs/chr8_110901034-110901634.png]

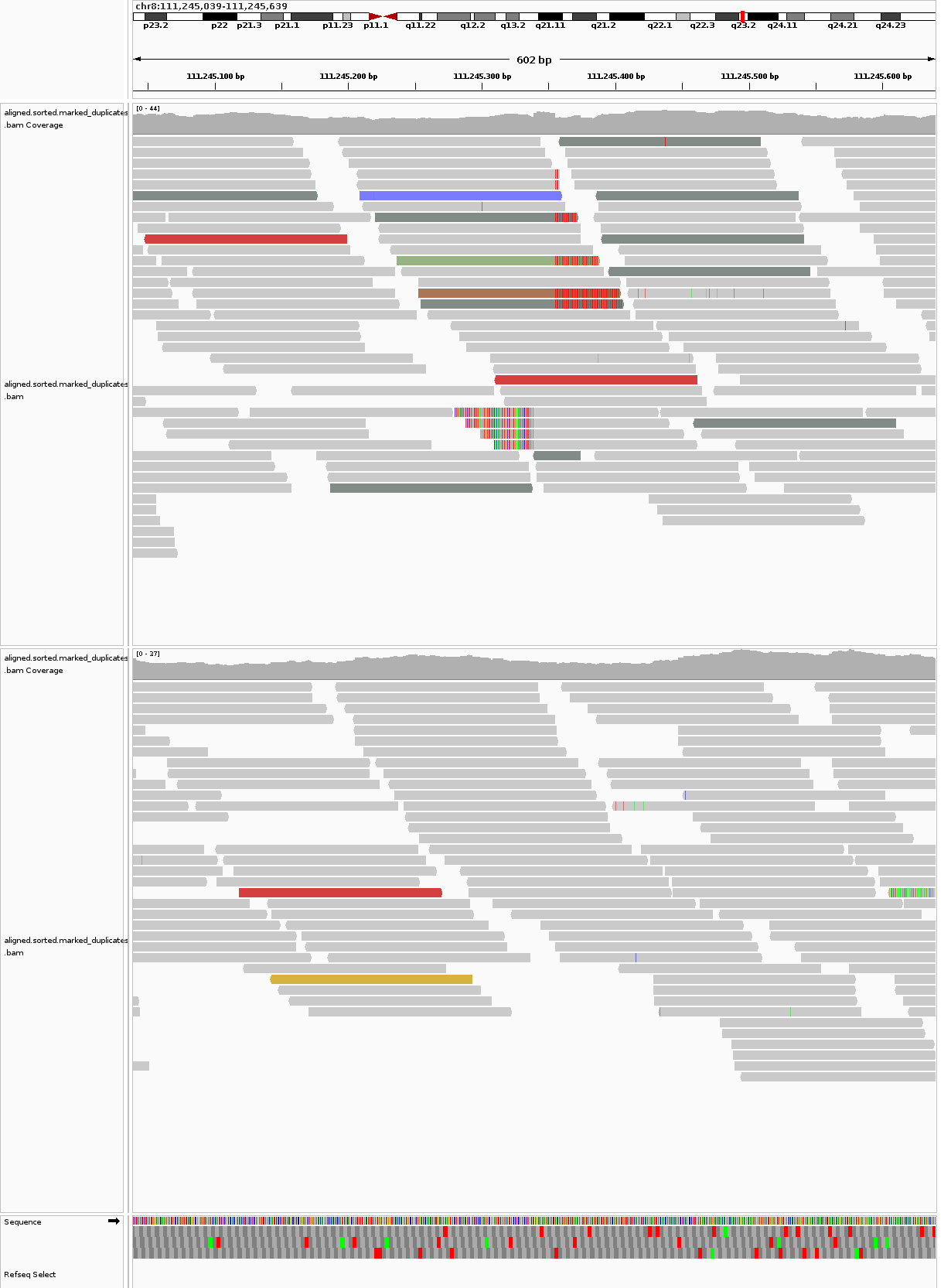

Supplement: Supplementary file 1 — Data S1. Compressed file containing the IGV screenshots for all the RetroTest exclusive insertions inspected in sample_21 and sample_28 WGS data, classified as true positives (TPs), false positives (FPs), and unconclusive. Both the tumor and normal BAM files were included in each screenshot. [file MOL2-19-3769-s003.zip › IGV_screenshots_illuminaWGS_TD2-RetroTest-exclusive_classified/PD0277a_retrotest_exclusive_IlluminaWGS/TPs/chr8_111245039-111245639.png]

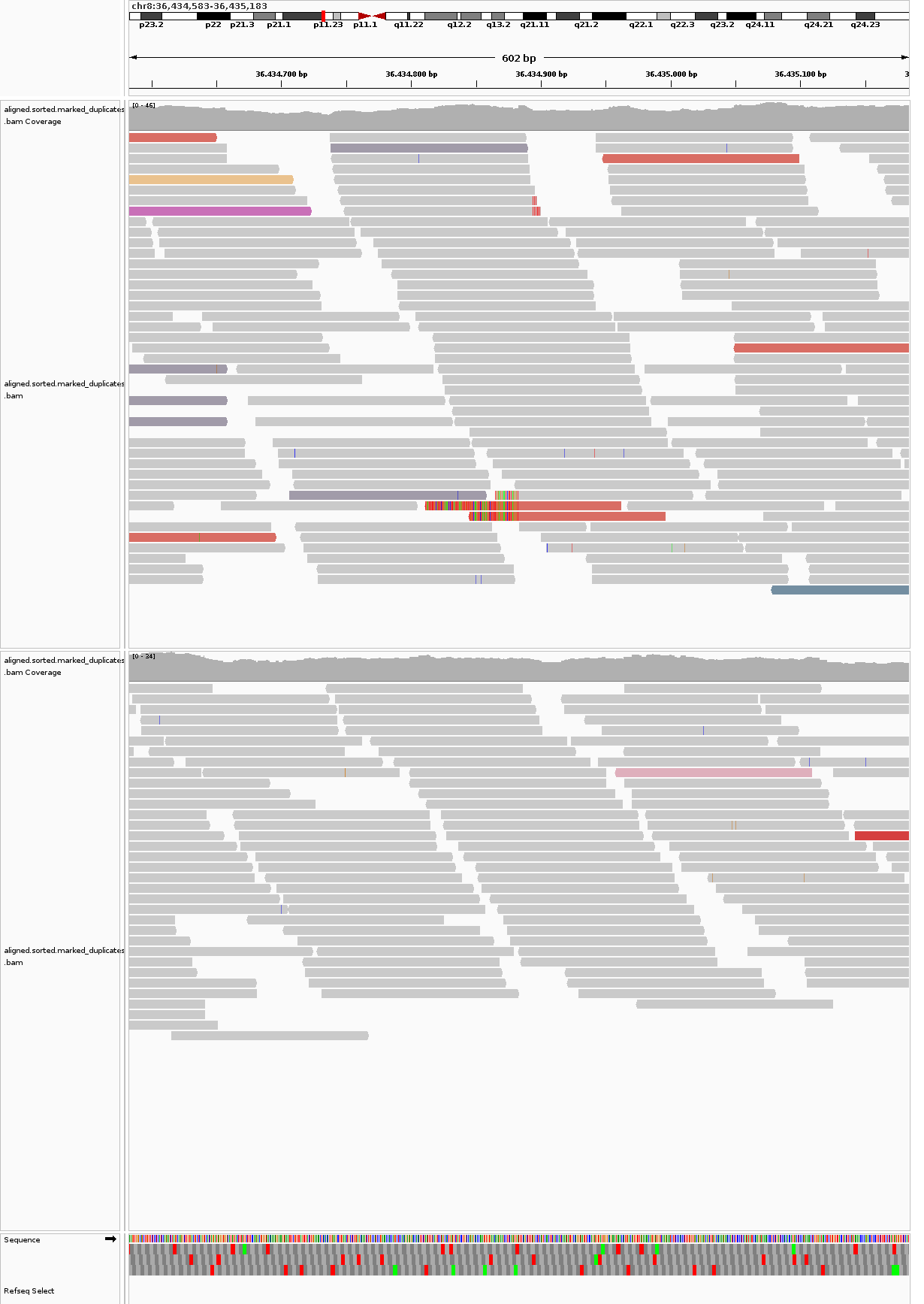

Supplement: Supplementary file 1 — Data S1. Compressed file containing the IGV screenshots for all the RetroTest exclusive insertions inspected in sample_21 and sample_28 WGS data, classified as true positives (TPs), false positives (FPs), and unconclusive. Both the tumor and normal BAM files were included in each screenshot. [file MOL2-19-3769-s003.zip › IGV_screenshots_illuminaWGS_TD2-RetroTest-exclusive_classified/PD0277a_retrotest_exclusive_IlluminaWGS/TPs/chr8_36434583-36435183.png]

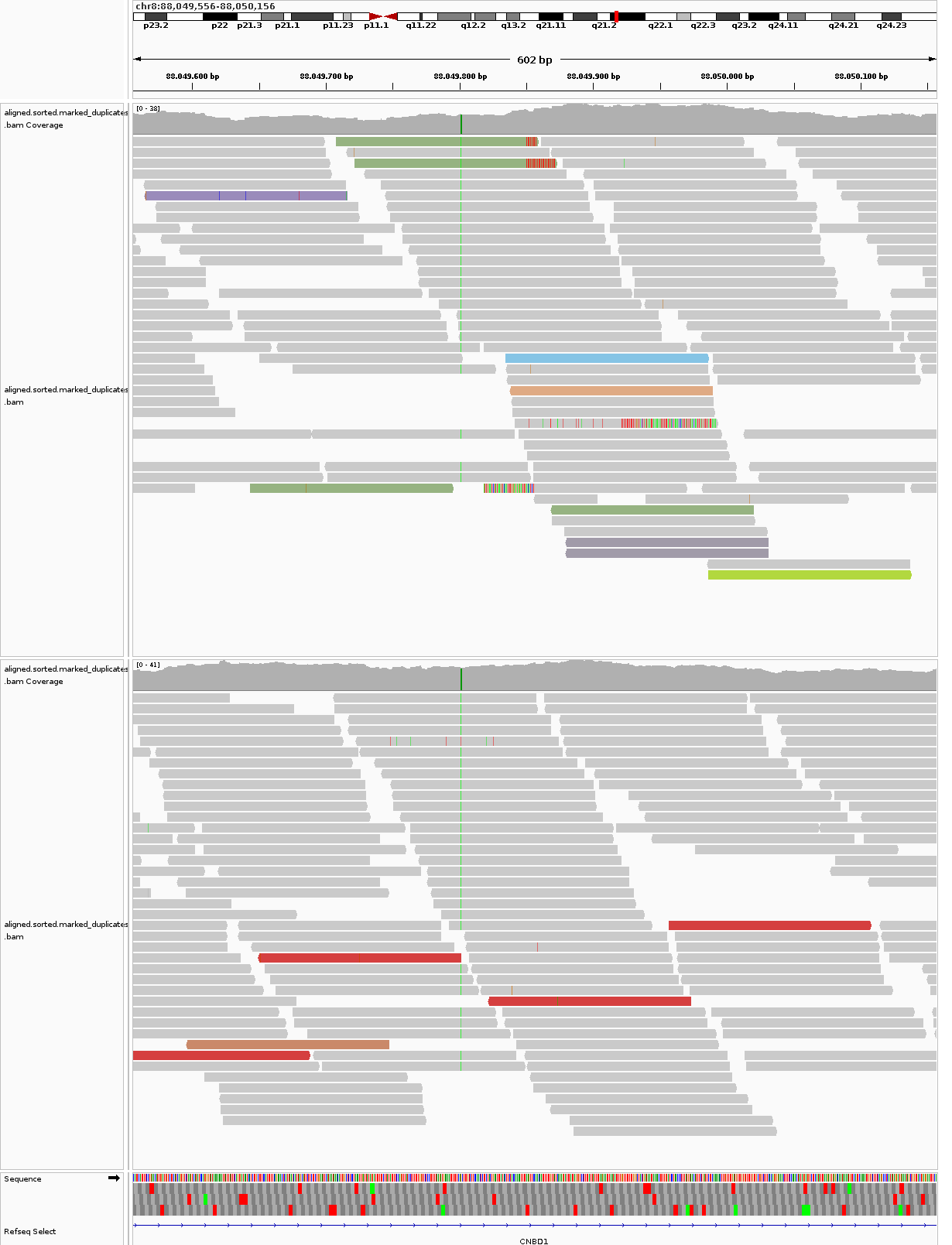

Supplement: Supplementary file 1 — Data S1. Compressed file containing the IGV screenshots for all the RetroTest exclusive insertions inspected in sample_21 and sample_28 WGS data, classified as true positives (TPs), false positives (FPs), and unconclusive. Both the tumor and normal BAM files were included in each screenshot. [file MOL2-19-3769-s003.zip › IGV_screenshots_illuminaWGS_TD2-RetroTest-exclusive_classified/PD0277a_retrotest_exclusive_IlluminaWGS/TPs/chr8_88049556-88050156.png]

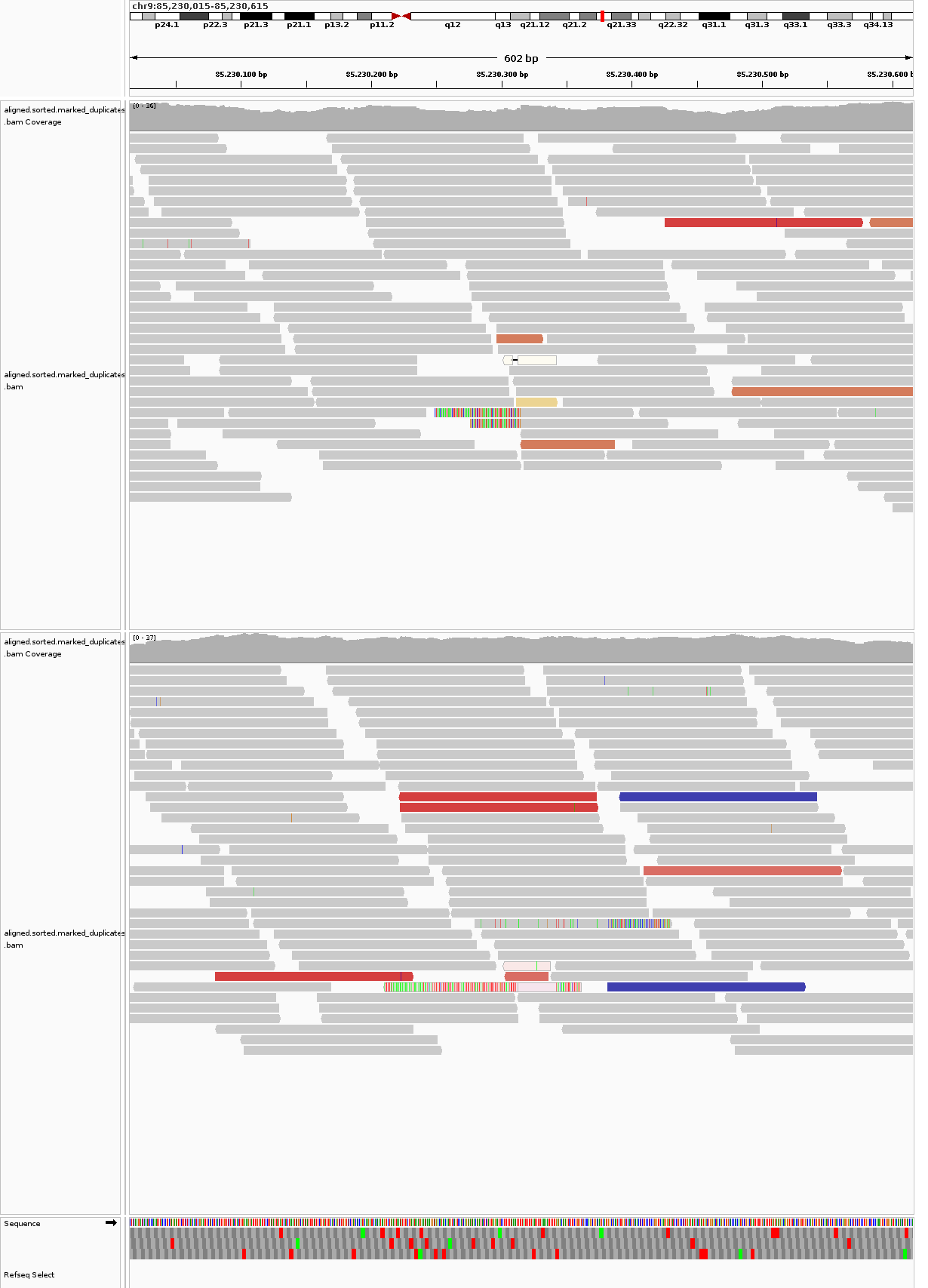

Supplement: Supplementary file 1 — Data S1. Compressed file containing the IGV screenshots for all the RetroTest exclusive insertions inspected in sample_21 and sample_28 WGS data, classified as true positives (TPs), false positives (FPs), and unconclusive. Both the tumor and normal BAM files were included in each screenshot. [file MOL2-19-3769-s003.zip › IGV_screenshots_illuminaWGS_TD2-RetroTest-exclusive_classified/PD0277a_retrotest_exclusive_IlluminaWGS/TPs/chr9_85230015-85230615.png]

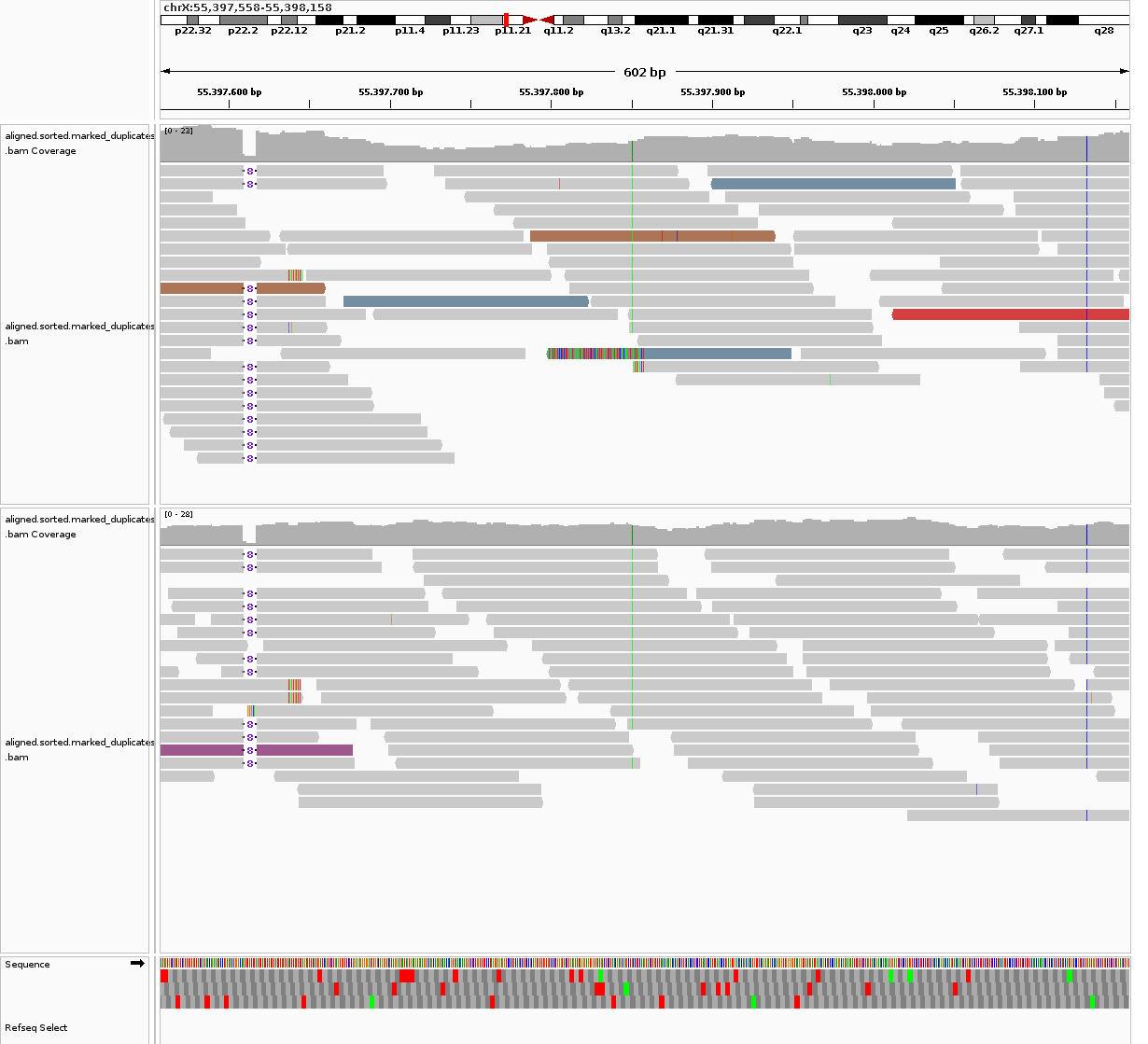

Supplement: Supplementary file 1 — Data S1. Compressed file containing the IGV screenshots for all the RetroTest exclusive insertions inspected in sample_21 and sample_28 WGS data, classified as true positives (TPs), false positives (FPs), and unconclusive. Both the tumor and normal BAM files were included in each screenshot. [file MOL2-19-3769-s003.zip › IGV_screenshots_illuminaWGS_TD2-RetroTest-exclusive_classified/PD0277a_retrotest_exclusive_IlluminaWGS/TPs/chrX_55397558-55398158.png]

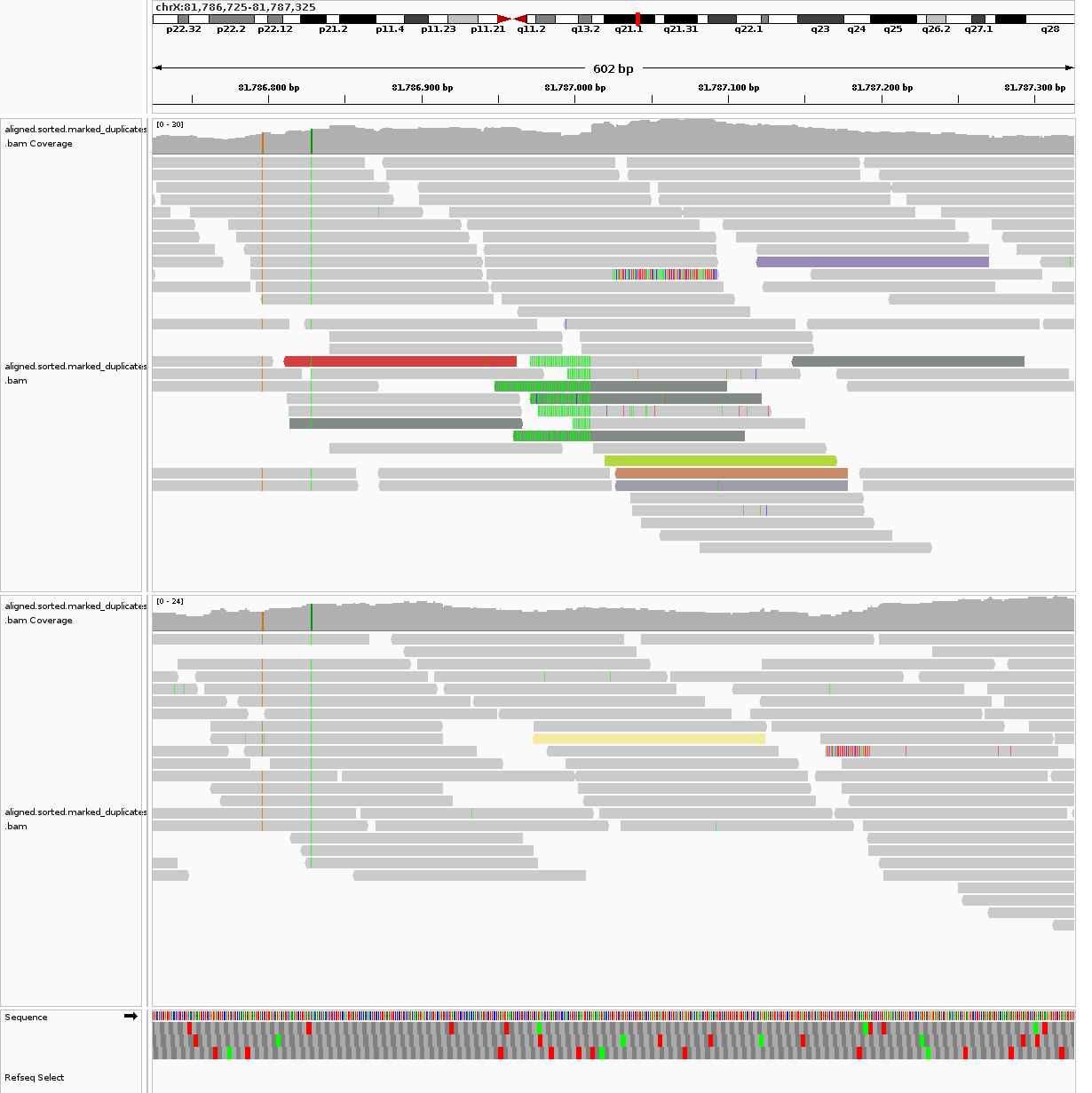

Supplement: Supplementary file 1 — Data S1. Compressed file containing the IGV screenshots for all the RetroTest exclusive insertions inspected in sample_21 and sample_28 WGS data, classified as true positives (TPs), false positives (FPs), and unconclusive. Both the tumor and normal BAM files were included in each screenshot. [file MOL2-19-3769-s003.zip › IGV_screenshots_illuminaWGS_TD2-RetroTest-exclusive_classified/PD0277a_retrotest_exclusive_IlluminaWGS/TPs/chrX_81786725-81787325.png]

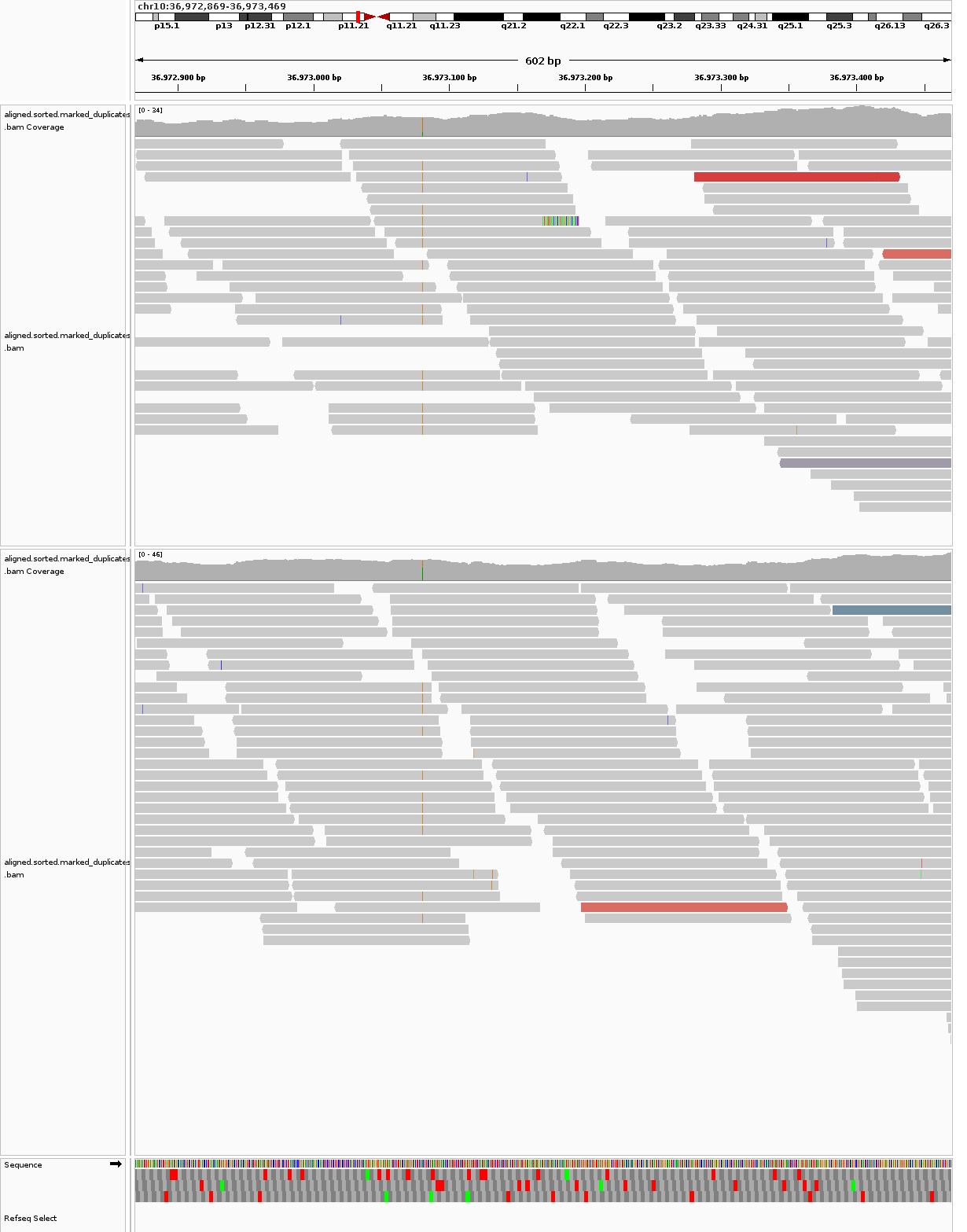

Supplement: Supplementary file 1 — Data S1. Compressed file containing the IGV screenshots for all the RetroTest exclusive insertions inspected in sample_21 and sample_28 WGS data, classified as true positives (TPs), false positives (FPs), and unconclusive. Both the tumor and normal BAM files were included in each screenshot. [file MOL2-19-3769-s003.zip › IGV_screenshots_illuminaWGS_TD2-RetroTest-exclusive_classified/PD0277a_retrotest_exclusive_IlluminaWGS/unconclusive/chr10_36972869-36973469.png]

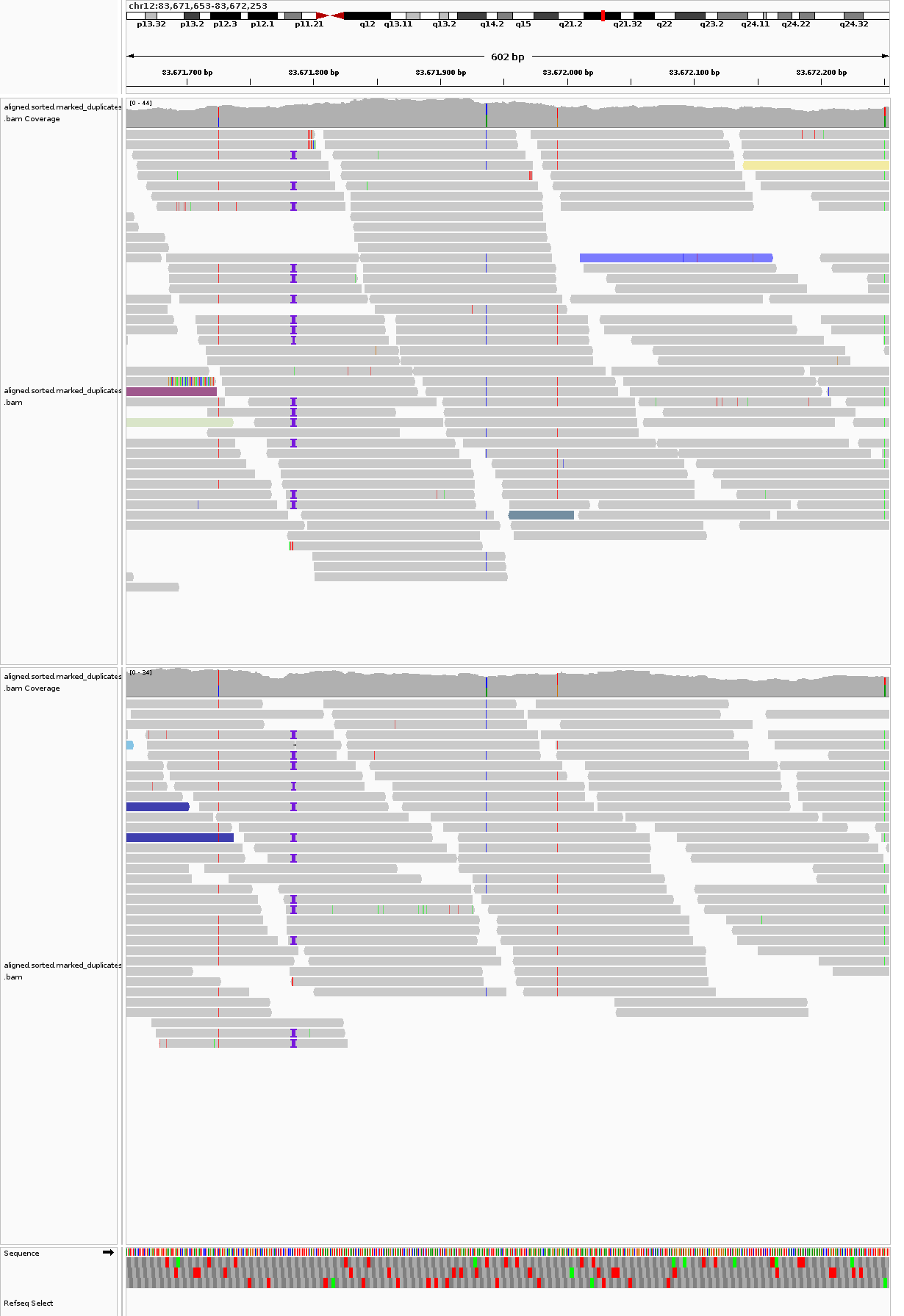

Supplement: Supplementary file 1 — Data S1. Compressed file containing the IGV screenshots for all the RetroTest exclusive insertions inspected in sample_21 and sample_28 WGS data, classified as true positives (TPs), false positives (FPs), and unconclusive. Both the tumor and normal BAM files were included in each screenshot. [file MOL2-19-3769-s003.zip › IGV_screenshots_illuminaWGS_TD2-RetroTest-exclusive_classified/PD0277a_retrotest_exclusive_IlluminaWGS/unconclusive/chr12_83671653-83672253.png]

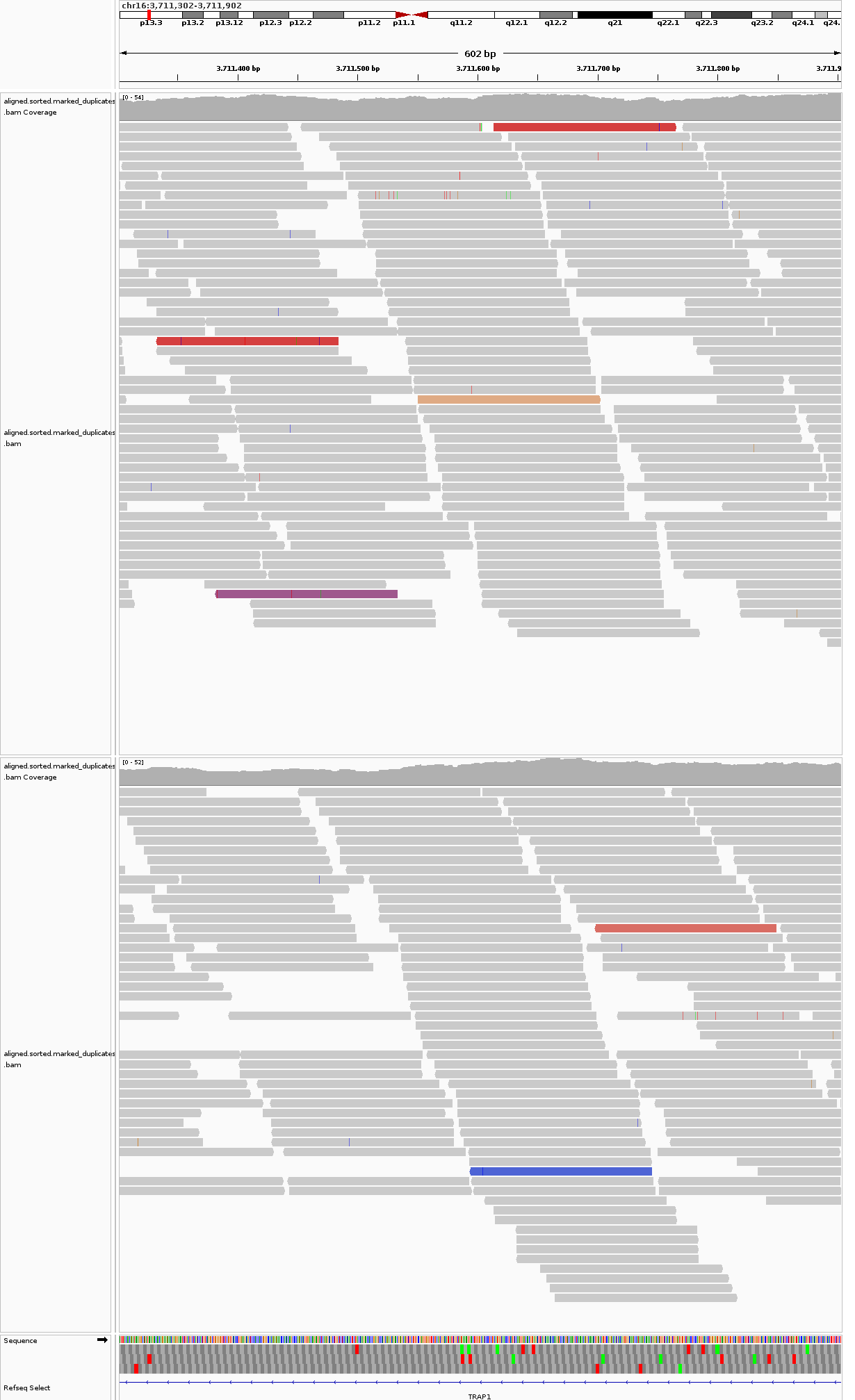

Supplement: Supplementary file 1 — Data S1. Compressed file containing the IGV screenshots for all the RetroTest exclusive insertions inspected in sample_21 and sample_28 WGS data, classified as true positives (TPs), false positives (FPs), and unconclusive. Both the tumor and normal BAM files were included in each screenshot. [file MOL2-19-3769-s003.zip › IGV_screenshots_illuminaWGS_TD2-RetroTest-exclusive_classified/PD0277a_retrotest_exclusive_IlluminaWGS/unconclusive/chr16_3711302-3711902.png]

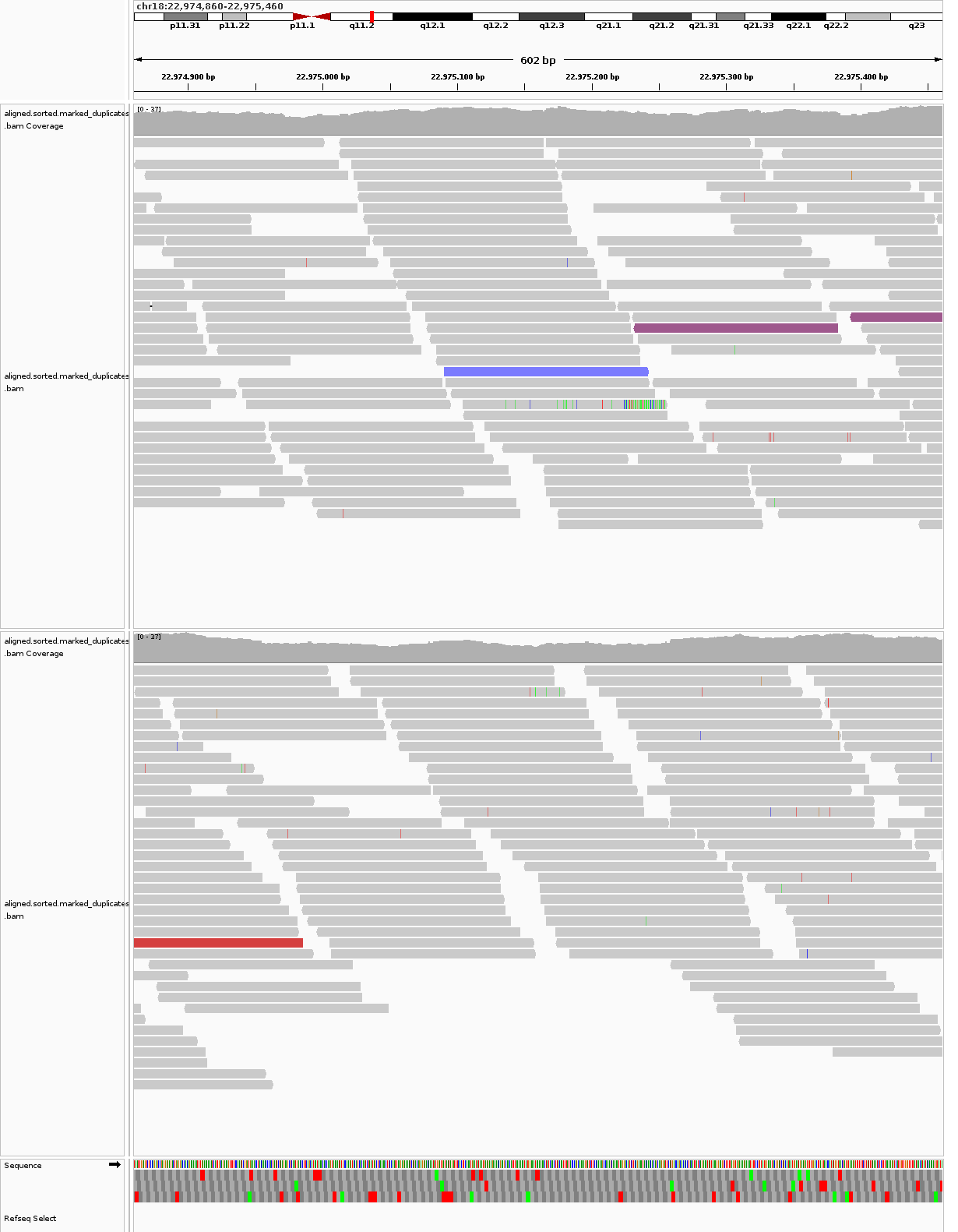

Supplement: Supplementary file 1 — Data S1. Compressed file containing the IGV screenshots for all the RetroTest exclusive insertions inspected in sample_21 and sample_28 WGS data, classified as true positives (TPs), false positives (FPs), and unconclusive. Both the tumor and normal BAM files were included in each screenshot. [file MOL2-19-3769-s003.zip › IGV_screenshots_illuminaWGS_TD2-RetroTest-exclusive_classified/PD0277a_retrotest_exclusive_IlluminaWGS/unconclusive/chr18_22974860-22975460.png]

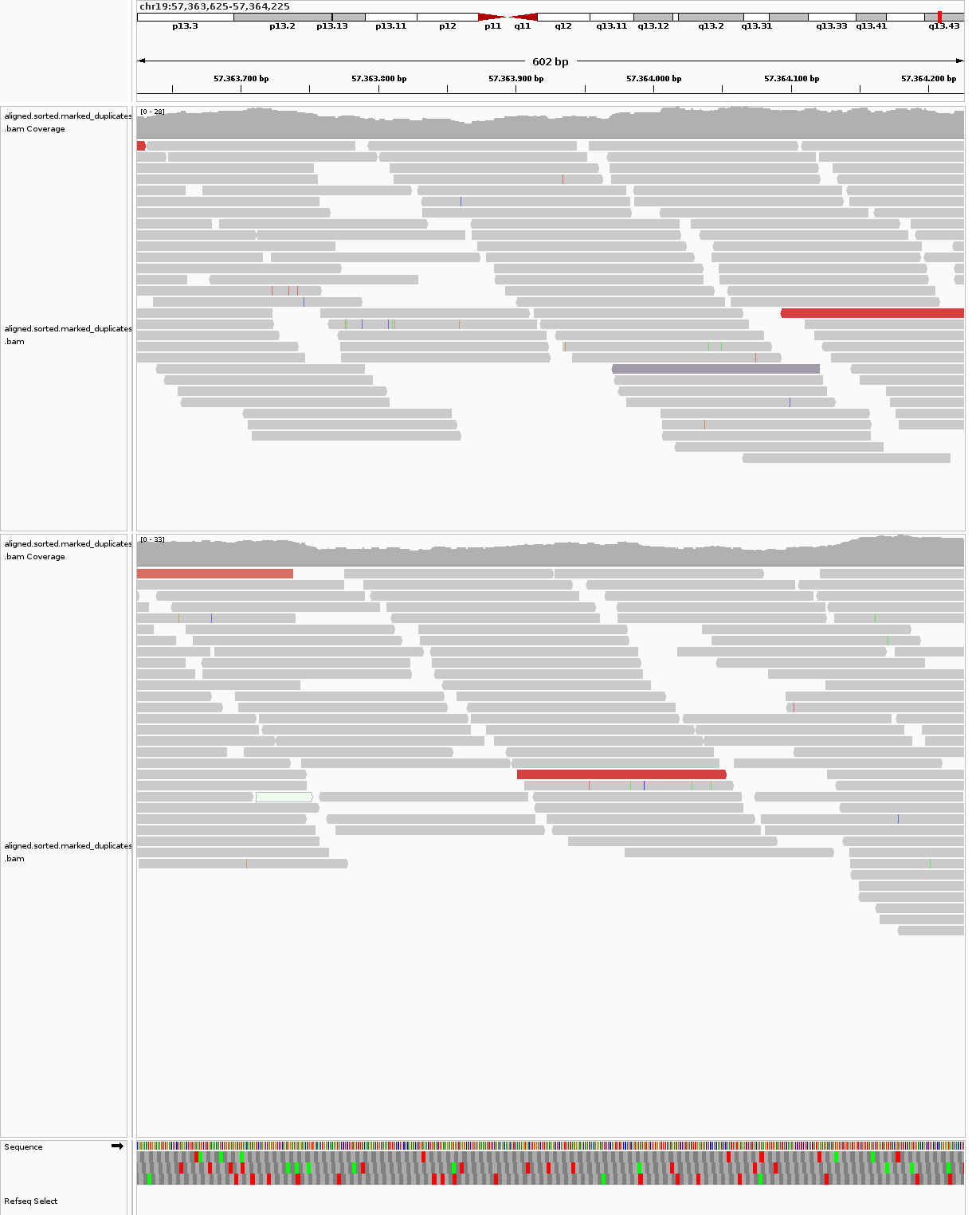

Supplement: Supplementary file 1 — Data S1. Compressed file containing the IGV screenshots for all the RetroTest exclusive insertions inspected in sample_21 and sample_28 WGS data, classified as true positives (TPs), false positives (FPs), and unconclusive. Both the tumor and normal BAM files were included in each screenshot. [file MOL2-19-3769-s003.zip › IGV_screenshots_illuminaWGS_TD2-RetroTest-exclusive_classified/PD0277a_retrotest_exclusive_IlluminaWGS/unconclusive/chr19_57363625-57364225.png]

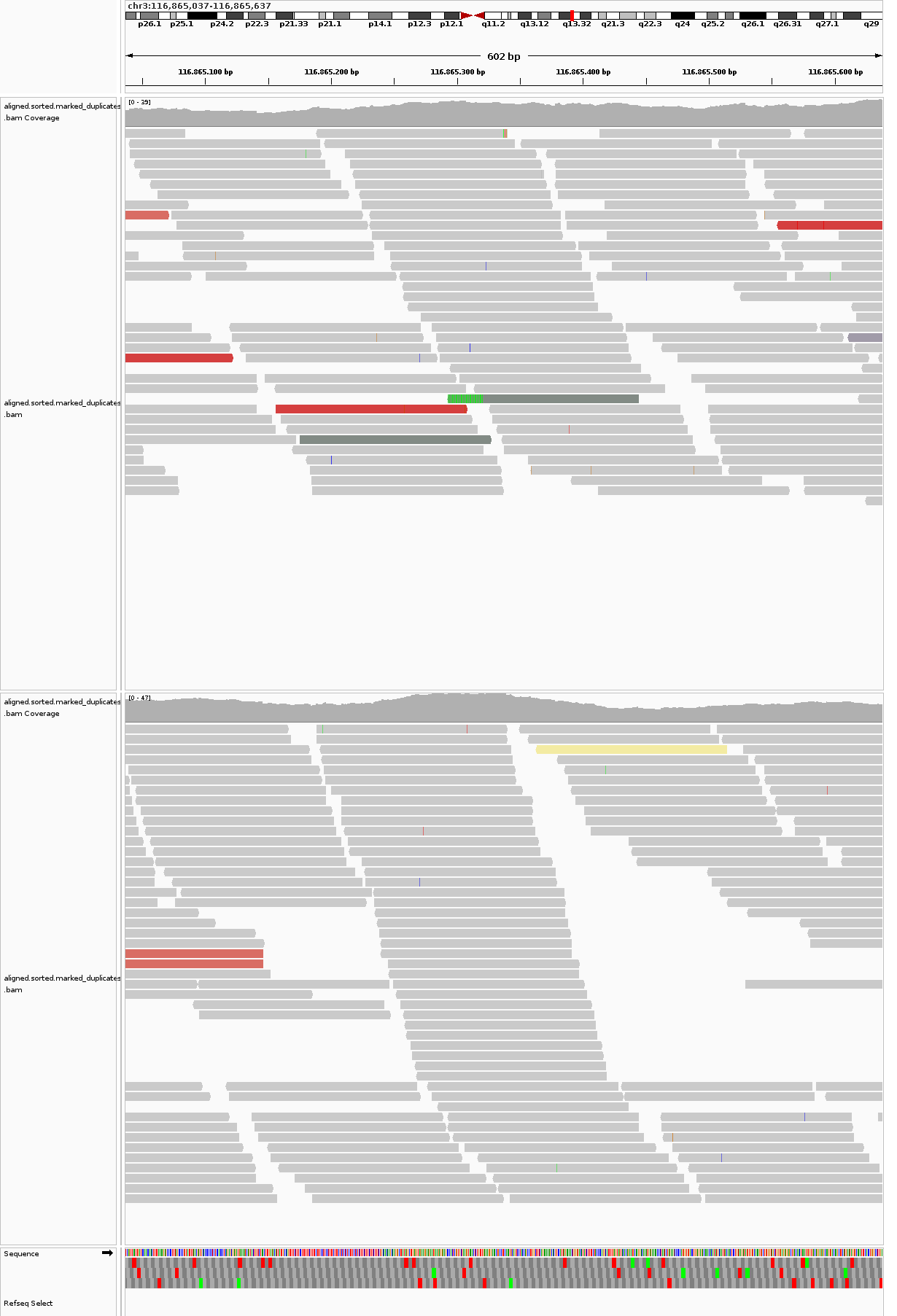

Supplement: Supplementary file 1 — Data S1. Compressed file containing the IGV screenshots for all the RetroTest exclusive insertions inspected in sample_21 and sample_28 WGS data, classified as true positives (TPs), false positives (FPs), and unconclusive. Both the tumor and normal BAM files were included in each screenshot. [file MOL2-19-3769-s003.zip › IGV_screenshots_illuminaWGS_TD2-RetroTest-exclusive_classified/PD0277a_retrotest_exclusive_IlluminaWGS/unconclusive/chr3_116865037-116865637.png]

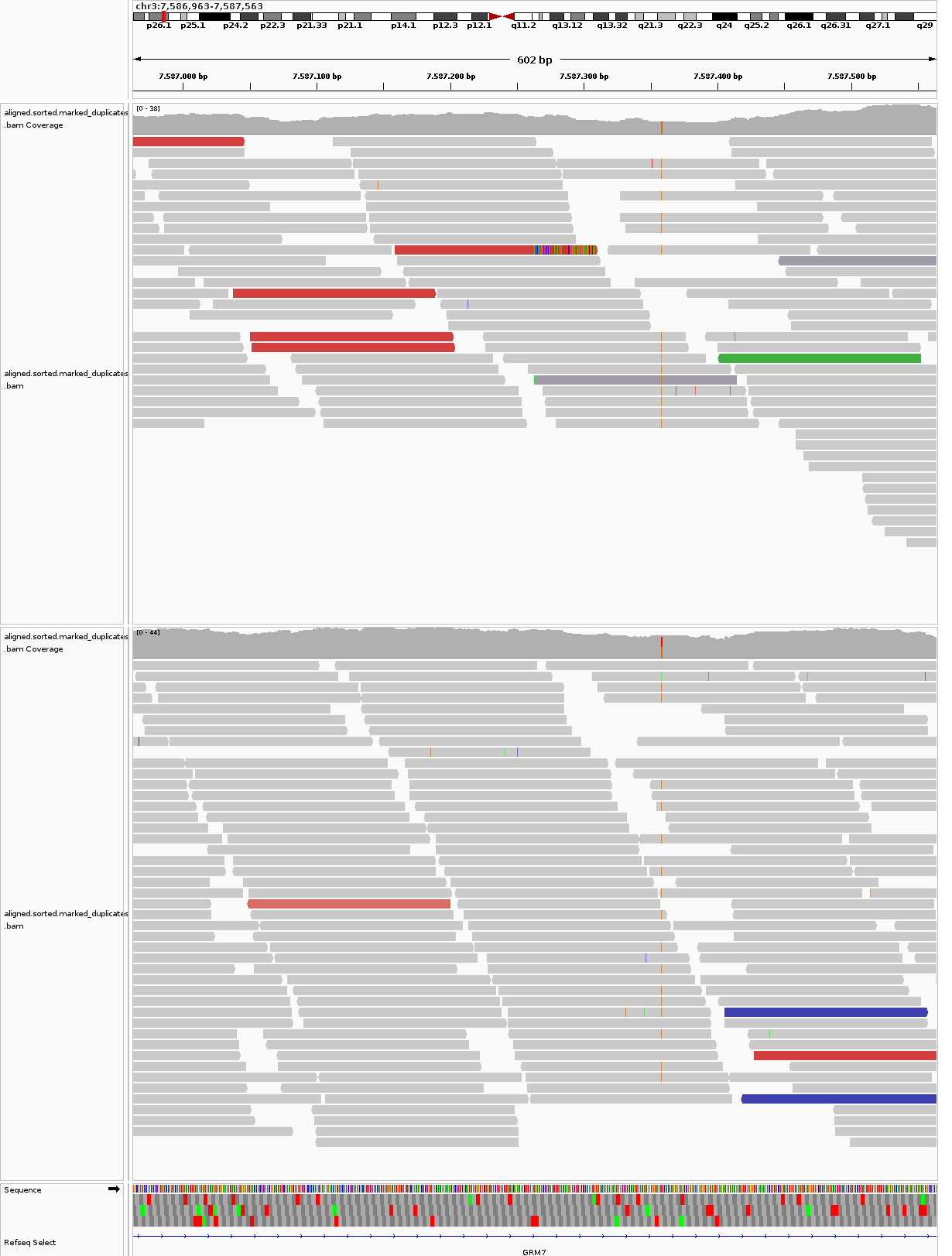

Supplement: Supplementary file 1 — Data S1. Compressed file containing the IGV screenshots for all the RetroTest exclusive insertions inspected in sample_21 and sample_28 WGS data, classified as true positives (TPs), false positives (FPs), and unconclusive. Both the tumor and normal BAM files were included in each screenshot. [file MOL2-19-3769-s003.zip › IGV_screenshots_illuminaWGS_TD2-RetroTest-exclusive_classified/PD0277a_retrotest_exclusive_IlluminaWGS/unconclusive/chr3_7586963-7587563.png]

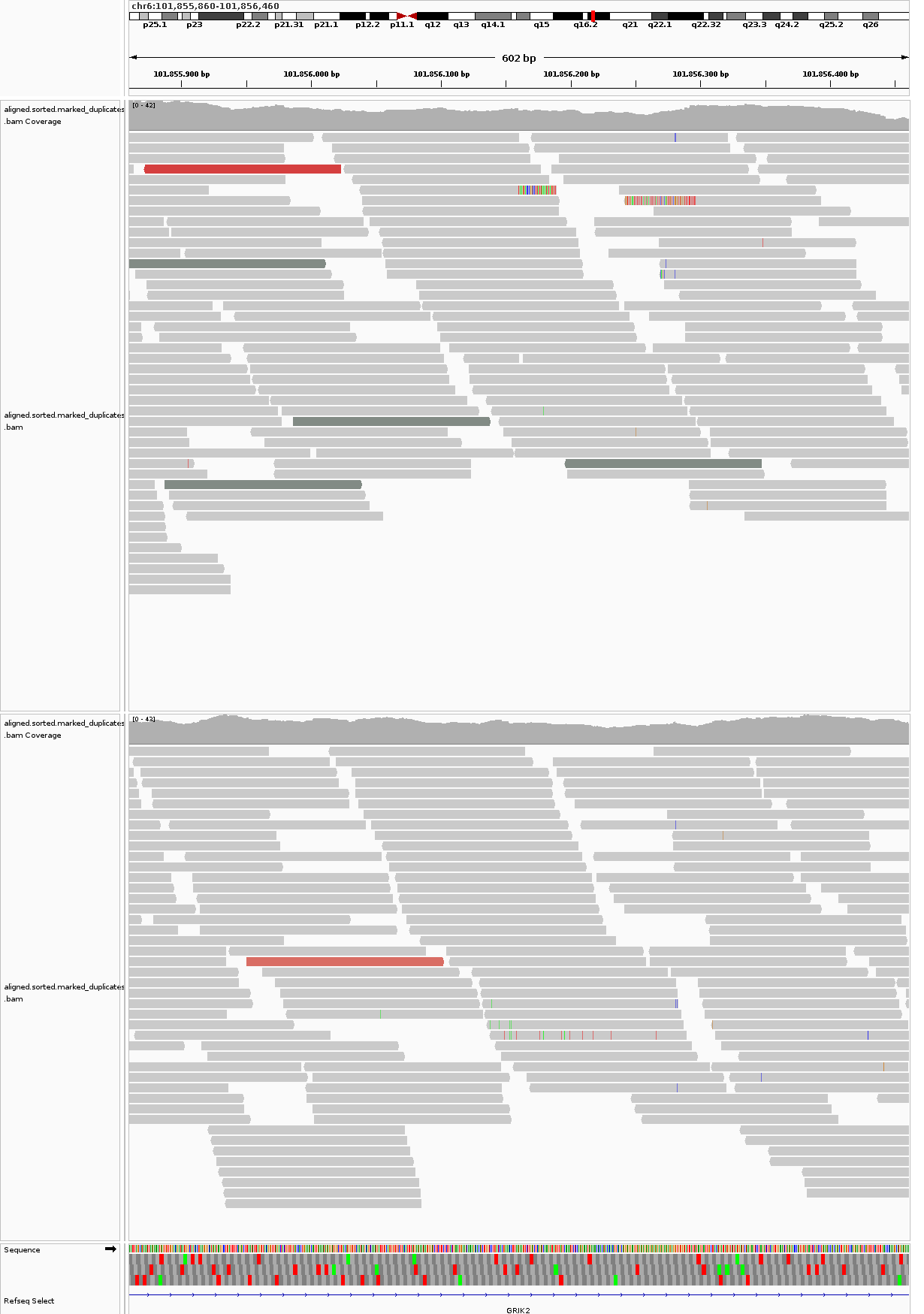

Supplement: Supplementary file 1 — Data S1. Compressed file containing the IGV screenshots for all the RetroTest exclusive insertions inspected in sample_21 and sample_28 WGS data, classified as true positives (TPs), false positives (FPs), and unconclusive. Both the tumor and normal BAM files were included in each screenshot. [file MOL2-19-3769-s003.zip › IGV_screenshots_illuminaWGS_TD2-RetroTest-exclusive_classified/PD0277a_retrotest_exclusive_IlluminaWGS/unconclusive/chr6_101855860-101856460.png]
