# Supplementary material for: Unraveling LINE‐1 retrotransposition in head and neck squamous cell carcinoma
Source: Mol Oncol. 2025 Jun 4;19(12):3769–83. doi: 10.1002/1878-0261.70063 (PMC12688165; doi:10.1002/1878-0261.70063)
Supplement: Supplementary file 2 — Fig. S1. Integrative Genomics Viewer inspection of WGS data of RetroTest private retrotranspositions. Fig. S2. Kaplan–Meier curves for overall survival in HNSCC cohort with respect to (A) L1 activation status (active vs inactive) and (B) L1 activation rate (with respect to the median, being high above vs low as below the median). Log‐rank test was used to calculate the P‐value. [file MOL2-19-3769-s002.pdf]

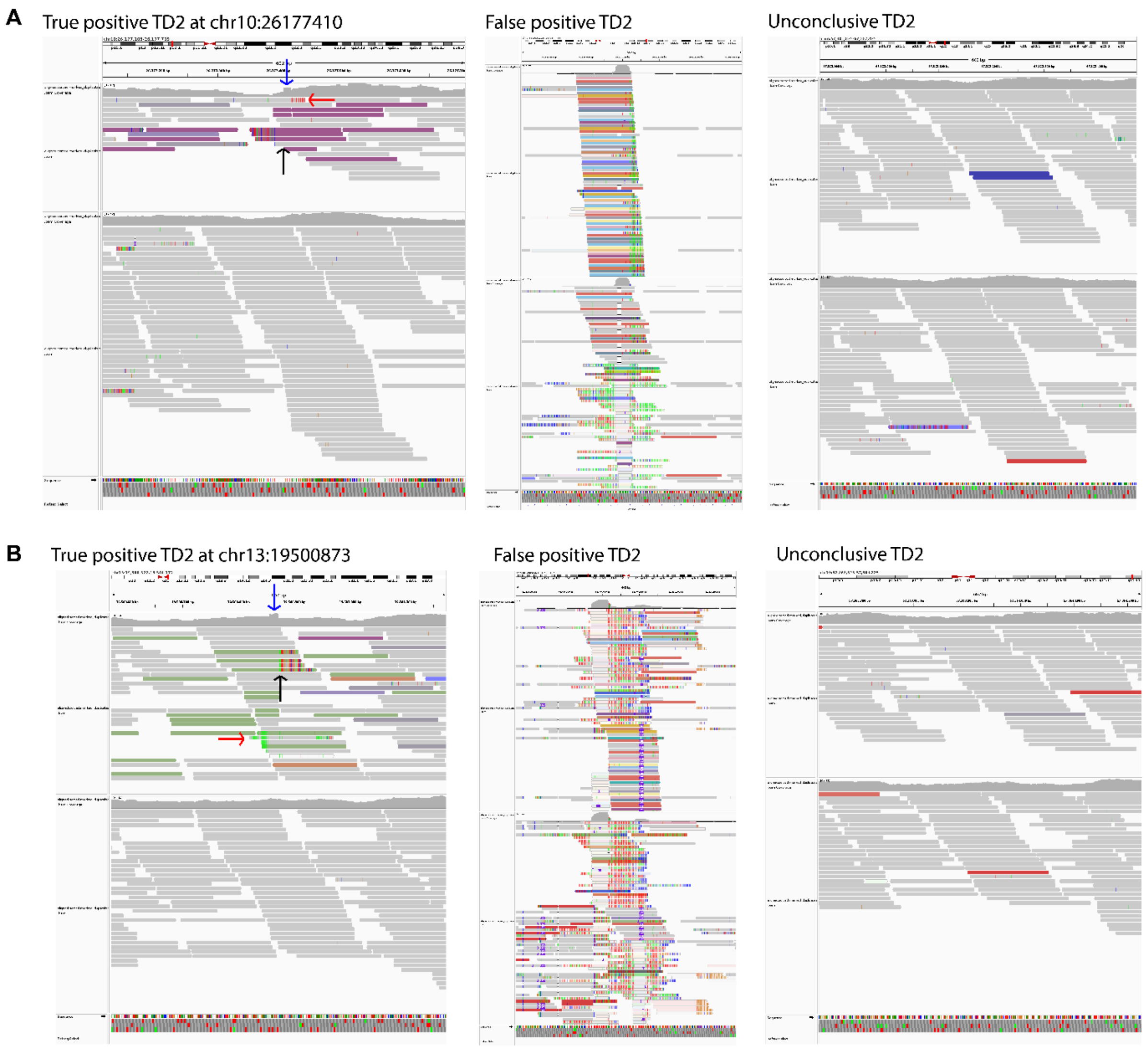

Figure S1. Integrative Genomics Viewer inspection of WGS data of RetroTest private retrotranspositions. **A.** Examples of TD2 insertions for sample_21 WGS data. **B.** Examples of TD2 insertions for sample_28 WGS data. Both the tumor and normal BAM files were included in each screenshot. The hallmarks that confirm the retrotransposition are highlighted with arrows: the presence of TSD (blue arrow), a poly (A/T) tail (red arrow), and a precisely defined breakpoint (black arrow).

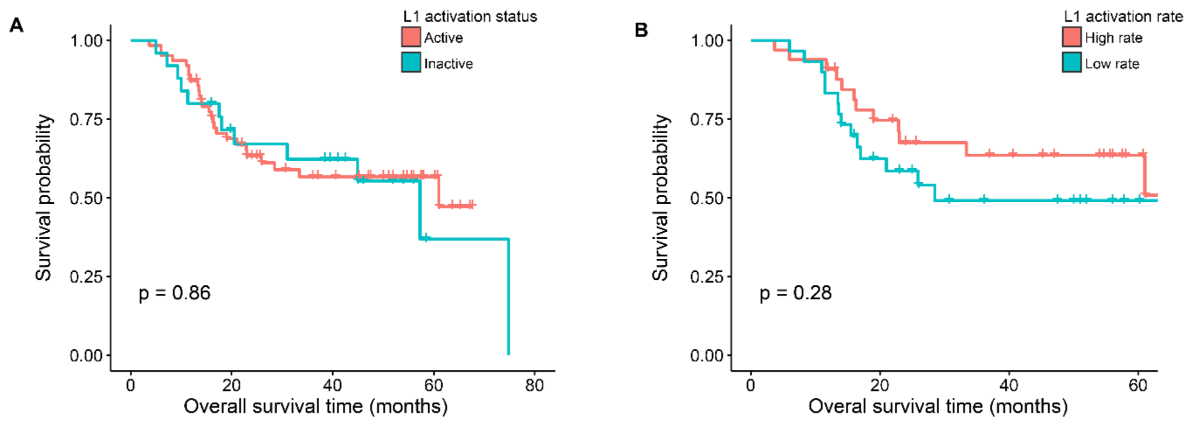

Figure S2. Kaplan-Meier curves for overall survival in HNSCC cohort with respect to (A) L1 activation status (active vs inactive) and (B) L1 activation rate (with respect to the median, being high above vs low as below the median). Log-rank test was used to calculate the p-value.
